# Supplementary material for: The economics of physical activity in low-income and middle-income countries: protocol for a systematic review
Source: BMJ Open. 2019 Jan 17;9(1):e022686. doi: 10.1136/bmjopen-2018-022686 (PMC6340626; doi:10.1136/bmjopen-2018-022686)
Supplement: Supplementary file 2 [file bmjopen-2018-022686supp002.pdf]

Supplementary file 2: Proposed search strategy for Scopus

	Proposed preliminary search strategy for Scopus
#1	Domain 1: Physical activity<br>*“physical activity"* OR *"physical exercise"* OR *"physical inactivity"* OR *"physical fitness"* OR *"Sedentary"* OR “*active lifestyle*” OR “active *living”* OR “active *leisure”* OR “active *play”* OR “active *recreation”* OR “active *commuting"*
#2	Domain 2: Economics<br>*"economics"* OR *"economic evaluation"* OR *"economic burden"* OR *"health care cost"* OR “*cost analyses”* OR “cost *effectiveness”* OR “cost *benefit”* OR “cost *minimization”* OR “cost *utility”* OR “cost *saving”* OR “cost *outcome*” OR “cost *consequence*”<br>OR *"demand"* OR *"supply"* OR *"market"* OR *"price"* OR *"incentives"* OR *"willingness to pay"* OR *"time use"*
#3	#1 AND #2
