# Supplementary material for: The economics of physical activity in low-income and middle-income countries: protocol for a systematic review
Source: BMJ Open. 2019 Jan 17;9(1):e022686. doi: 10.1136/bmjopen-2018-022686 (PMC6340626; doi:10.1136/bmjopen-2018-022686)
Supplement: Supplementary file 3 [file bmjopen-2018-022686supp003.pdf]

Supplementary file 3

DRAFT DATA EXTRACTION FORM FOR REVIEW ON ECONONOMICS AND PYSICAL ACTIVITY IN LOW AND MIDDLE INCOME COUNTRIES (ADAPTED FROM MURTHEY ET AL., 2017, POKHREL ET AL., 2014, PAVEY ET AL., 2011)			
No.	Item category	Items	Notes
1	General information	1.1 Study ID<br>1.2 Review author ID<br>1.3 Date of review<br>1.4 Title, authors(s)<br>1.5 Year of publication<br>1.6 Country studies<br>1.7 Income level of the country	
2	Characteristics of the study	2.1 Aims and objectives/research question<br>2.2. Eligibility decision/Inclusion and exclusion criteria (?)<br>2.3 Study design<br>2.4 Study setting<br>2.5 Total study duration<br>2.6 Sample size<br>2.7 Sample size calculation<br>2.8 Recruitment<br>2.9 Sample selection<br>2.10 Method of allocation<br>2.11 Perspective (i.e. Societal, health care system, service provider etc.)	
3	Characteristics of the population/condition /intervention	**Population/participants**<br>3.1 Type of population<br>(i.e. General population university students, group of employees)<br>3.2 Age<br>3.3 Gender<br>3.4 Ethnicity<br>**Condition**<br>3.5 Definition of physical activity /physical inactivity/sedentary	

		3.6Definition of disease condition<br>**Intervention**<br>3.7 Type of physical activity/programme<br>3.9 Comparator	
4	Data sources/Data analysis/results	**Scope**<br>4.1 Type of analysis<br>(Ie. Costing/economic evaluation/association of economic factors with physical activity/other)<br>4.2 Form of economic evaluation<br>4.3 Time horizon<br>4.4 Study assumptions<br>**Cost**<br>4.5 Reported costs/cost components<br>4.6 Data sources for cost measures<br>4.7 How cost was reported<br>(i.e. marginal cost/ average cost/incremental cost/total cost/other)<br>4.8 Discount rate<br>4.9 Year of costing<br>4.10 Currency unit<br>4.11 Currency conversion rate<br>4.12 Inflation adjustments<br>**Outcomes**<br>4.13 Reported outcomes<br>(i.e. measures of effectiveness/efficacy/measures of effect)<br>4.14 Data sources for the outcome measures<br>4.15 Discount rate<br>**Sensitivity analysis**<br>4.16 Performed/ not<br>4.17 Type of analysis<br>(i.e. deterministic/ stochastic)<br>4.18 Variables used for sensitivity analysis<br>4.19 Results of sensitivity analysis<br>**Main results**	

		4.20 Cost / cost effectiveness<br>4.21 Other	
5	Conclusions and way forward	5.1 Key Conclusion(s)<br>5.2 Suggestions by author for further research	
6	Challenges	6.1 Author stated limitations<br>6.2 Author stated strengths<br>6.3 Miscellaneous comments from author<br>6.4 Funding source<br>6.5 Conflicts of interest	
7.	Quality assessment of studies	7.1 Economic evaluation studies- Drummond checklist<br>7.2 Model based economic evaluations-Phillips check list<br>7.3 Cost of illness/cost of risk factor study/cost estimation- check list by Larg and Moss<br>7.4 Quantitative study reporting correlation or association? NICE check list for quantitative studies reporting correlations and associations	

**Method of rating the quality of studies from each check list (A, B, C, D) in accordance to the quality appraisal by NICE scale (Methods for the development of NICE public health guidance (third edition))**	
• Reviewer is requested to complete in detail the comment section of quality appraisal check lists so that the grade awarded for each study aspect is as transparent as possible<br>• At the end of completion of the check list each study is awarded an overall quality grading for internal validity (IV)and a separate one for external validity (EV)	
All or most of the checklist criteria have been fulfilled, where they have not been fulfilled the conclusions are very unlikely to alter	++
Some of the checklist criteria have been fulfilled, where they have not been fulfilled, or not adequately described, the conclusions are unlikely to alter	+
Few or no checklist criteria have been fulfilled and the conclusions are likely or very likely to alter.	-

CHECKLIST A

QUALITY ASSESSMENT FOR ECONOMIC EVALUATION(CHECKLIST FROM DRUMMOND AND JEFFERSON 1996)	Yes	No	Not clear	Not appropriate
**Study design**				
1) The research question is stated				
2) The economic importance of the research question is stated				
3) The viewpoint(s) of the analysis are clearly stated and justified				
4) The rationale for choosing the alternative programmes or interventions compared is stated				
5) The alternatives being compared are clearly described				
6) The form of economic evaluation used is stated				
7) The choice of form of economic evaluation is justified in relation to the questions addressed				
**Data collection**				
8) The source(s) of effectiveness estimates used are stated				
9) Details of the design and results of effectiveness study are given (if based on a single study)				
10) Details of the method of synthesis or meta-analysis of estimates are given (if based on an overview of a number				

QUALITY ASSESSMENT FOR ECONOMIC EVALUATION(CHECKLIST FROM DRUMMOND AND JEFFERSON 1996)	Yes	No	Not clear	Not appropriate
of effectiveness studies)				
11) The primary outcome measure(s) for the economic evaluation are clearly stated				
12) Methods to value health states and other benefits are stated				
13) Details of the subjects from whom valuations were obtained are given				
14) Productivity changes (if included) are reported separately				
15) The relevance of productivity changes to the study question is discussed				
16) Quantities of resources are reported separately from their unit costs				
17) Methods for the estimation of quantities and unit costs are described				
18) Currency and price data are recorded				
19) Details of currency of price adjustments for inflation or currency conversion are given				
20) Details of any model used are given				
21) The choice of model used and the key parameters on which it is based are justified				
**Analysis and interpretation of results**				
22) Time horizon of costs and benefits is stated				
23) The discount rate(s) is stated				

QUALITY ASSESSMENT FOR ECONOMIC EVALUATION(CHECKLIST FROM DRUMMOND AND JEFFERSON 1996)	Yes	No	Not clear	Not appropriate
24) The choice of rate(s) is justified				
25) An explanation is given if costs or benefits are not discounted				
26) Details of statistical tests and confidence intervals are given for stochastic data				
27) The approach to sensitivity analysis is given				
28) The choice of variables for sensitivity analysis is justified				
29) The ranges over which the variables are varied are stated				
30) Relevant alternatives are compared				
31) Incremental analysis is reported				
32) Major outcomes are presented in a disaggregated as well as aggregated form				
33) The answer to the study question is given				
34) Conclusions follow from the data reported				
35) Conclusions are accompanied by the appropriate caveats				

Did the economic evaluation use a decision-analytic modelling framework?	Response: Yes(✓), No (✗) , Not applicable (NA)<br><br>Instruction: If *Yes*, assess paper using the questions in Check list B, If *No/NA*, skip check list B

CHECKLIST B

QUALITY ASSESSMENT FOR DECISION-ANALYTIC MODELS(CHECKLIST FROM PHILIPS ET AL. 2004)			
**Quality criterion**	**Question(s)**	**Response (✓, ✗, NA)**	**Comments**
S1	Is there a clear statement of the decision problem?		
	Is the objective of the evaluation and model specified and consistent with the stated decision problem?		
	Is the primary decision maker specified?		
S2	Is the perspective of the model stated clearly?		
	Are the model inputs consistent with the stated perspective?		
	Has the scope of the model been stated and justified?		
	Are the outcomes of the model consistent with the perspective, scope and overall objective of the model?		
S3	Is the structure of the model consistent with a coherent theory of the health condition under evaluation?		
	Are the sources of data used to develop the structure of the model specified?		

QUALITY ASSESSMENT FOR DECISION-ANALYTIC MODELS(CHECKLIST FROM PHILIPS ET AL. 2004)			
**Quality criterion**	**Question(s)**	**Response (✓, ✗, NA)**	**Comments**
	Are the causal relationships described by the model structure justified appropriately?		
S4	Are the structural assumptions transparent and justified?		
	Are the structural assumptions reasonable given the overall objective, perspective and scope of the model?		
S5	Is there a clear definition of the options under evaluation?		
	Have all feasible and practical options been evaluated?		
	Is there justification for the exclusion for the exclusion of feasible options?		
S6	Is the chosen model type appropriate given the decision problem and specified causal relationships within the model?		
S7	Is the time horizon of the model sufficient to reflect all important differences between options?		
	Are the time horizon of the model, the duration of treatment and the duration of treatment effect described and justified?		
S8	Do the disease states (state transition model) or the pathways (decision tree model) reflect the underlying biological process of the		

QUALITY ASSESSMENT FOR DECISION-ANALYTIC MODELS(CHECKLIST FROM PHILIPS ET AL. 2004)			
**Quality criterion**	**Question(s)**	**Response (✓, ✗, NA)**	**Comments**
	disease in question and the impact of interventions?		
S9	Is the cycle length defined and justified in terms of natural history of disease?		
D1	Are the data identification methods transparent and appropriate given the objectives of the model?		
	Where choices have been made between data sources, are these justified appropriately?		
	Has particular attention been paid to identifying data for the important parameters in the model?		
	Has the quality of the data been assessed appropriately?		
	Where expert opinion has been used, are the methods described and justified?		
D2	Is the data modelling methodology based on justifiable statistical and epidemiological techniques?		
D2a	Is the choice of baseline data described and justified?		
	Are transition probabilities calculated appropriately?		

QUALITY ASSESSMENT FOR DECISION-ANALYTIC MODELS(CHECKLIST FROM PHILIPS ET AL. 2004)			
**Quality criterion**	**Question(s)**	**Response (✓, ✗, NA)**	**Comments**
	Has a half-cycle correction been applied to both cost and outcome?		
	If not, has this omission been justified?		
D2b	If relative treatment effects have been derived from trail data, have they been synthesised using appropriate techniques?		
	Have the methods and assumptions used to extrapolate short-term results to final outcomes been documented and justified?		
	Have alternative extrapolation assumptions been explored through sensitivity analysis?		
	Have assumptions regarding the continuing effect of treatment once treatment is complete been documented and justified?		
	Have alternative assumptions regarding the continuing effect of treatment been explored through sensitivity analysis?		
D2c	Are the costs incorporated into the model justified?		
	Has the source for all costs been described?		
	Have discount rates been described and justified given the target decision-maker?		

QUALITY ASSESSMENT FOR DECISION-ANALYTIC MODELS(CHECKLIST FROM PHILIPS ET AL. 2004)			
**Quality criterion**	**Question(s)**	**Response (✓, ✗, NA)**	**Comments**
D2d	Are the utilities incorporated into the model appropriate?		
	Is the source for the utility weights referenced?		
	Are the methods of derivation for the utility weights justified?		
D3	Have all data incorporated into the model been described and referenced in sufficient detail?		
	Has the use of mutually inconsistent data been justified (i.e. are assumptions and choices appropriate)?		
	Is the process of data incorporation transparent?		
	If data have been incorporated as distributions, has the choice of distribution for each parameter been described and justified?		
	If data have been incorporated as distributions, is it clear that second order uncertainty is reflected?		
D4	Have the four principal types of uncertainty been addressed?		
	If not, has the omission of particular forms of uncertainty been justified?		
D4a	Have methodological uncertainties been addressed by running alternative versions of the model with different methodological		

QUALITY ASSESSMENT FOR DECISION-ANALYTIC MODELS(CHECKLIST FROM PHILIPS ET AL. 2004)			
**Quality criterion**	**Question(s)**	**Response (✓, ✗, NA)**	**Comments**
	assumptions?		
D4b	Is there evidence that structural uncertainties have been addressed via sensitivity analysis?		
D4c	Has heterogeneity been dealt with by running the model separately for different subgroups?		
D4d	Are the methods of assessment of parameter uncertainty appropriate?		
	If data are incorporated at point estimates, are the ranges used for sensitivity analysis stated clearly and justified?		
C1	Is there evidence that the mathematical logic of the model has been tested thoroughly before use?		
C2	Are any counterintuitive results from the model explained and justified?		
	If the model has been calibrated against independent data, has any differences been explained and justified?		
	Have the results of the model been compared with those of previous models and any differences in results explained?		

CHECKLIST C

**QUALITY ASSESSMNET FOR COST OF ILLNESS STUDIES (ADAPTED FROM LARG AND MOSS, 2011)**			
Responses (marked Similar to NICE check list, write the answer where relevant under the comment section)<br><br>++ Indicates that for that particular aspect of study design, the study has been designed or conducted in such a way as to minimise the risk of bias.<br>+ Indicates that either the answer to the checklist question is not clear from the way the study is reported, or that the study may not have addressed all potential sources of bias for that particular aspect of study design.<br>- Should be reserved for those aspects of the study design in which significant sources of bias may persist.<br>NR (Not Reported) - Should be reserved for those aspects in which the study under review fails to report how they have (or might have) been considered.<br>NA (Not Applicable) Should be reserved for those study design aspects that are not applicable given the study design under review (for example, allocation concealment would not be applicable for case–control studies).			
	Question	Response ***++/+/-NR/NA***	Comment
1.	Analytical framework: what costs should have been measured?<br><br>(a) What was the motivation and perspective of the study?<br><br>(b) Was the appropriate epidemiologic approach taken?<br><br>(c) Was the study question well specified?<br>(i) Were all relevant, non-trivial cost components and their stakeholders identified?<br>(ii) Were necessary timeframes specified?<br>(iii) Was a case of disease or risk factor adequately and appropriately defined?<br>(iv) Was the counterfactual population occurrence plausible and meaningful?		
2.	(2) Methodology and data: how well were resource use and productivity losses measured?		

	(a) Was an appropriate method(s) of quantification used, such that;<br>(i) Additional, or excess, costs were measured?<br>(ii) only costs specific to (caused by) the health problem were included (confounders controlled)?<br>(iii) All important effects were captured?<br>(iv) Important differences across subpopulations were accounted for?<br>(v) The required level of detail could be provided?<br><br>(b) Was the resource quantification method(s) well executed?<br>(i) For population-based studies, were cost allocation methods, data and assumptions valid?<br>(ii) For person-based studies, were appropriate statistical tests performed and reported?<br>(iii) Were data representative of the study population?<br>(iv) Were there any other relevant resource quantification issues?<br><br>(c) Were healthcare resources valued appropriately?<br><br>(d) Was the approach for valuing production losses justified, and assumptions valid?<br><br>(e) Was the inclusion of intangible costs appropriate:<br>(i) Was double counting of mortality-related production losses avoided?<br>(ii) Were losses valued appropriately, given the study's perspective?		
3	Analysis and reporting<br>(a) Did the analysis address the study question?<br><br>(b) Was a range of estimates presented?		

	(c) Were the main uncertainties identified?<br><br>(d) Was a sensitivity analysis performed on:<br><br>(i) important (uncertain) parameter estimates?<br>(ii) key assumptions? (including the counterfactual)<br>(iii) point estimates? (based on confidence or credible intervals)<br><br>(e) Was adequate documentation and justification given for cost components, data and sources, assumptions and methods?<br><br>(f) Was uncertainty around the estimates and its implications adequately discussed?<br><br>(g) Were important limitations discussed regarding the cost components, data, assumptions and methods?<br><br>(h) Were the results presented at the appropriate level of detail to answer the study question (cost components; disease subtypes, severity, stage; subpopulation groups, cost bearers)?		

CHECKLIST D

**QUALITY ASSESSMENT FOR QUANTITATIVE STUDIES REPORTING CORRELATIONS AND ASSOCIATIONS(NICE,2012 BASED ON 'GRAPHICAL APPRAISAL TOOL FOR EPIDEMIOLOGICAL STUDIES(GATE)` BY JACKSON ET AL. (2006)**			
Responses:<br><br>++ Indicates that for that particular aspect of study design, the study has been designed or conducted in such a way as to minimise the risk of bias.<br><br>+ Indicates that either the answer to the checklist question is not clear from the way the study is reported, or that the study may not have addressed all potential sources of bias for that particular aspect of study design.<br><br>- Should be reserved for those aspects of the study design in which significant sources of bias may persist.<br><br>NR (Not Reported) - Should be reserved for those aspects in which the study under review fails to report how they have (or might have) been considered.<br><br>NA (Not Applicable) Should be reserved for those study design aspects that are not applicable given the study design under review (for example, allocation concealment would not be applicable for case–control studies).			
**Section 1: Population**			
**Number**	**Question**	**Response**<br><br>**++/+/-/ NR/NA**	**Comments**
1.1	Is the source population or source area well described?<br><br>Was the country (e.g. developed or non-developed, type of health care system), setting (primary schools, community centres etc), location (urban, rural), population demographics etc adequately described?		

1.2	Is the eligible population or area representative of the source population or area?<br><br>Was the recruitment of individuals, clusters or areas well defined (e.g. advertisement, birth register)?<br><br>Was the eligible population representative of the source? Were important groups underrepresented?		
1.3	Do the selected participants or areas represent the eligible population or area?<br><br>Was the method of selection of participants from the eligible population well described?<br><br>What % of selected individuals or clusters agreed to participate? Were there any sources of bias?<br><br>Were the inclusion or exclusion criteria explicit and appropriate?		
**Section 2: Method of selection of exposure (or comparison) group**			
2.1	Selection of exposure (and comparison) group. How was selection bias minimised?<br><br>How was selection bias minimised?		
2.2	Was the selection of explanatory variables based on a sound theoretical basis?<br><br>How sound was the theoretical basis for selecting the explanatory variables?		
2.3	Was the contamination acceptably low?<br><br>Did any in the comparison group receive the exposure?		

	If so, was it sufficient to cause important bias?		
2.4	How well were likely confounding factors identified and controlled?<br><br>Were there likely to be other confounding factors not considered or appropriately adjusted for?<br><br>Was this sufficient to cause important bias?		
**Section 3: Outcomes**			
3.1	Were the outcome measures and procedures reliable?<br><br>Were outcome measures subjective or objective (e.g. biochemically validated nicotine levels ++ vs self-reported smoking −)?<br><br>How reliable were outcome measures (e.g. inter- or intra-rater reliability scores)?<br><br>Was there any indication that measures had been validated (e.g. validated against a gold standard measure or assessed for content validity)?		
3.2	Were the outcome measurements complete?<br><br>Were all or most of the study participants who met the defined study outcome definitions likely to have been identified?		
3.3	Were all important outcomes assessed?<br><br>Were all benefits and harms assessed?<br><br>Was it possible to determine the overall balance of benefits and harms of		

	intervention versus comparisons?		
3.4	Was there a similar follow-up time in exposure and comparison groups?<br><br>If groups are followed for different lengths of time, then more events are likely to occur in the group followed-up for longer distorting the comparison.<br><br>Analyses can be adjusted to allow for differences in length of follow-up (e.g. using person-years).		
3.5	Was follow-up time meaningful?<br><br>Was follow-up long enough to assess long-term benefits and harms?<br><br>Was it too long, e.g. participants lost to follow-up?		
**Section 4: Analyses**			
4.1	Was the study sufficiently powered to detect an intervention effect (if one exists)?<br><br>A power of 0.8 (i.e. it is likely to see an effect of a given size if one exists, 80% of the time) is the conventionally accepted standard.<br><br>Is a power calculation presented? If not, what is the expected effect size? Is the sample size adequate?		
4.2	Were multiple explanatory variables considered in the analyses?<br><br>Were there sufficient explanatory variables considered in the analysis?		

4.3	Were the analytical methods appropriate?<br><br>Were important differences in follow-up time and likely confounders adjusted for?		
4.4	Was the precision of association given or calculable? Is association meaningful?<br><br>Were confidence intervals or p values for effect estimates given or possible to calculate?<br><br>Were CIs wide or were they sufficiently precise to aid decision-making? If precision is lacking, is this because the study is under-powered?		
**Section 5: Summary**			
5.1	Are the study results internally valid (i.e. unbiased)?<br><br>How well did the study minimise sources of bias (i.e. adjusting for potential confounders)?<br><br>Were there significant flaws in the study design?		
5.2	Are the findings generalizable to the source population (i.e. externally valid)?<br><br>Are there sufficient details given about the study to determine if the findings are generalizable to the source population?<br><br>Consider: participants, interventions and comparisons, outcomes, resource and policy implications		
