# Supplementary material for: The economics of physical activity in low-income and middle-income countries: protocol for a systematic review
Source: BMJ Open. 2019 Jan 17;9(1):e022686. doi: 10.1136/bmjopen-2018-022686 (PMC6340626; doi:10.1136/bmjopen-2018-022686)
Supplement: Supplementary file 4 [file bmjopen-2018-022686supp004.pdf]

Supplementary file 4

Summary of Quality assessment tools used in systematic reviews from literature

	Quality assessment tools	Cochrane collaboration tool	Consensus health economic criteria (CHEC)	NICE	CHEERS statement	Drummond	PHILIPS	Larg & Moss cost of illness check list	Cost validity scale	National Collaborating Centre for Methods and Tools, 2008. Quality Assessment Tool for Quantitative Studies.
Systematic reviews and the type of economic analysis										
1.Murthey et al, 2017[1]										
• Trial l based economic evaluation(EE) studies		+	+		+	+				
• Model based EE				+	+		+			
• Cost benefit analyses									+	
• Cost of illness studies								+		
2.Aminde et al , 2018										
• EE						+	+			
• Model based EE							+			
3.Pavey et al, 2012										
• EE						+	+			
4.Ruifrok et al, 2014										
• EE		+								
5.Abu-Omar, 2017										
• Cost effectiveness(CE)										+
6.Vijay et al, 2015										
• CE						+				
7.Tonmukayakul et al, 2015										
• EE						+				

Conclusions derived based on summary of standard check lists used to assess the quality of economic studies in health research;

- Drummond 1996 was commonly used as the standard check list to assess the quality of studies on economic evaluations across the years (2011- 2018) and had been identified as the most appropriate on this regard [1,2, 3,6,7]
- Phillips checklist has been used as the standard check list to assess the quality of model based economic evaluations[1,2,3]
- Larg & Moss checklist for quality assessment in cost of illness studies has been used in review conducted recently in LMIC [1]. This is more relevant and appropriate to assess the quality of cost of illness studies.
- Quality grading has been allocated to Drummond 1996 checklist using NICE scale [2]

References

1. Murthy, S., John, D., Godinho, I. P., Godinho, M. A., Guddattu, V., & Nair, N. S. (2017). A protocol for a systematic review of economic evaluation studies conducted on neonatal systemic infections in South Asia. *Syst Rev, 6*(1), 252. doi: 10.1186/s13643-017-0648-7

2. Aminde, L. N., & Veerman, L. (2016). Interventions for the prevention of cardiovascular diseases: a protocol for a systematic review of economic evaluations in low-income and middle-income countries. *BMJ Open, 6*(12), e013668. doi: 10.1136/bmjopen-2016-013668

3. Pavey, T. G., Taylor, A. H., Fox, K. R., Hillsdon, M., Anokye, N., Campbell, J. L., Taylor, R. S. (2011). Effect of exercise referral schemes in primary care on physical activity and improving health outcomes: systematic review and meta-analysis. *BMJ, 343*. doi: 10.1136/bmj.d6462

4. Ruifrok, A. E., Rogozinska, E., van Poppel, M. N., Rayanagoudar, G., Kerry, S., de Groot, C. J., Thangaratinam, S. (2014). Study protocol: differential effects of diet and physical activity based interventions in pregnancy on maternal and fetal outcomes—individual patient data (IPD) meta-analysis and health economic evaluation. [journal article]. *Systematic Reviews, 3*(1), 131. doi: 10.1186/2046-4053-3-131

5. Abu-Omar, K., Rütten, A., Burlacu, I., Schätzlein, V., Messing, S., & Suhrcke, M. (2017). The cost-effectiveness of physical activity interventions: A systematic review of reviews. *Preventive Medicine Reports*, *8*, 72–78. http://doi.org/10.1016/j.pmedr.2017.08.006

6. GC, Vijay, Wilson, E. C., Suhrcke, M., Hardeman, W., & Sutton, S. (2015). Are brief interventions to increase physical activity cost-effective? A systematic review. *British Journal of Sports Medicine*. doi: 10.1136/bjsports-2015-094655

7. Tonmukayakul, U., Calache, H.,Clark, R., Wasiak, J., Faggion Jr, C.M. (2015). *Systematic review and quality appraisal of economic evaluation publications in Dentistry.* Journal of Dental research,94(10), 1348-1354 https://doi.org/10.1177/0022034515589958
