# Supplementary material for: The economics of physical activity in low-income and middle-income countries: protocol for a systematic review
Source: BMJ Open. 2019 Jan 17;9(1):e022686. doi: 10.1136/bmjopen-2018-022686 (PMC6340626; doi:10.1136/bmjopen-2018-022686)
Supplement: Author's manuscript [file bmjopen-2018-022686.draft_revisions.pdf]

BMJ Open

BMJ Open is committed to open peer review. As part of this commitment we make the peer review history of every article we publish publicly available.

When an article is published we post the peer reviewers' comments and the authors' responses online. We also post the versions of the paper that were used during peer review. These are the versions that the peer review comments apply to.

The versions of the paper that follow are the versions that were submitted during the peer review process. They are not the versions of record or the final published versions. They should not be cited or distributed as the published version of this manuscript.

BMJ Open is an open access journal and the full, final, typeset and author-corrected version of record of the manuscript is available on our site with no access controls, subscription charges or pay-per-view fees (http://bmjopen.bmj.com).

If you have any questions on BMJ Open's open peer review process please email info.bmjopen@bmj.com

BMJ Open

The economics of physical activity in low and middle income countries: protocol for a systematic review

Journal:	*BMJ Open*
Manuscript ID	bmjopen-2018-022686
Article Type:	Protocol
Date Submitted by the Author:	05-Mar-2018
Complete List of Authors:	Ranasinghe, Priyanga; Ministry of Health, Nutrition and Indigenous Medicine, ; Brunel Univeristy London, Clinical Sciences<br>Pokhrel, Subhash; Brunel University London, Health Economics Research Group<br>Anokye, Nana; Brunel University, Health Economics Research Group
Keywords:	HEALTH ECONOMICS, physical activity, low and middle income countries

SCHOLARONE™
Manuscripts

Title page

The economics of physical activity in low and middle income countries: protocol for a systematic review

Priyanga Diloshini Ranasinghe[2], Subhash Pokhrel[1], Nana Kwame Anokye[1]

[1] Health Economics Theme, Institute of Environment, Health and Societies, Brunel University, London, United Kingdom

[2] Ministry of Health, Nutrition and Indigenous Medicine, Suwasiripaya, Colombo 10, Sri Lanka /Division of Health Sciences, Department of Clinical Sciences, Collage of Health and Life Sciences, Brunel University, London, United Kingdom

Corresponding author: Priyanga Diloshini Ranasinghe

Postal address: Mary Seacole 2nd floor, Division of Health Sciences, Department of Clinical Sciences, Collage of Health and Life Sciences, Brunel University, London, United Kingdom

Email: Priyanga.Ranasinghe@brunel.ac.uk/ priyangaran@yahoo.com

Telephone number: 0044 7424515136/0094 777552794

Subhash Pokhrel Subhash.Pokhrel@brunel.ac.uk

Nana Kwame Anokye Nana.Anokye@brunel.ac.uk

Word count: 2165

For peer review only

Abstract

Introduction: Evidence on the economic costs of physical inactivity, how to mitigate these costs via different interventions, and the cost-effectiveness of physical activity promotion interventions in low- and middle-income countries (LMIC) is sparse, and fragmented where they are available. We aim to conduct a systematic review of the evidence on economics of physical activity in LMIC, with a view to identifying gaps in knowledge and provide recommendations for economic research agenda in LMIC.

Methods and analysis: Peer reviewed journal articles of observational, experimental, quasi experimental studies and mixed method studies on economics of physical activity in LMIC will be identified by an electronic data base search; Scopus, Web of Science and SPORTDiscus. Study selection process will be carried out in a two stage approach; Title and abstract screen for inclusion followed by review of selected full text articles by two independent reviewers. Disagreements will be resolved by consensus and discussion with a third reviewer. Data will be extracted using standardized piloted data extraction forms. Risk of bias will be critically appraised using standardised check lists based on study designs. Descriptive synthesis of obtained data is planned. Where the numbers permit, summaries of studies will be classified according to type of economic analysis, country or region, and intervention type. Meta-analysis will be performed where appropriate. This protocol for systematic review prepared according to Preferred Reporting Items for Systematic review and Meta-analysis for Protocols (PRISMA-P) -2015 statement.

Ethics and dissemination: Ethical approval will not be required as no original data will be collected as part of this review. The completed review will be submitted for publication in a peer-reviewed journal and presentation at conferences.

Systematic review registration number: This protocol will be registered with the International Prospective Register of Systematic Reviews (PROSPERO) following peer review.

For peer review only

Keywords: low and middle income countries; health economics; physical activity

Strengths and limitations of this study:

- This study is the first of its kind to review the evidence base on the economics of physical activity in the context of low and middle income countries.
- This study will identify gaps in knowledge to support future economic studies in the area.
- This study will not include publications that are not peer-reviewed such as unpublished theses and conference presentations.
- Studies published in any language other than English will not be included for this review.

For peer review only

INTRODUCTION

Rationale

Physical inactivity is a global health challenge, the fourth leading cause of mortality worldwide [1] and the key risk factor for non-communicable diseases (NCD) such as cardiovascular diseases, cancer and diabetes [2]. Almost one third of global health burden comprise of NCD which account for 1.4 billion disability adjusted life years (DALYs) [3]. At present most of the global NCD burden is from LMIC (as defined by World Bank, Classification 2017)[4] and this burden is largely attributable to population levels of physical inactivity[5,6]. Prevalence of physical inactivity in 2010 in upper middle income countries was 25.4% (19.1%-33.7%), followed by 16.8% (11.8%-26.4%) in lower middle income countries and 16.6% (11.8%-26.4%) in low income countries showing that large population in

LMIC[7] are affected by physical inactivity and are therefore at risk of developing NCDs. The data available for the year 2004 shows that 3.2 million deaths worldwide were due to physical inactivity of which 2.6 million were reported from LMIC [8]. As LMIC has experienced urbanization, shift of occupations from agriculture to industrial practice and modernization including automation, this structural transition has resulted in increased level of physical inactivity in LMIC populations [9].

Increasing prevalence of physical activity and reducing the burden of NCD is the goal of public health policies in LMIC [10]. It is a personal, cooperate and government responsibility to reduce the economic burden of unhealthy lifestyles in LMIC [10]. Economic analysis could uncover subtle characteristics of individual decision making on lifestyle choices; estimate the opportunity costs of not doing anything to improve population levels of physical activity; and help evaluate what interventions works efficiently to achieve the goal of addressing the physical inactivity pandemic particularly in LMIC [11]. Current focus to promote physical activity has shifted from traditional educational approach to environment and policy approaches. This trend has given more importance to economic perspectives of physical activity to complement public health perspective [12].

Economic perspectives inform the design of effective physical activity interventions (identifying economic barriers to physical activity) and the efficient allocation of resources – critical to the economies of LMIC [12]. However, the economic evidence on physical activity in LMIC is limited and scattered. Our scoping exercise identified no published reviews that address the economics of physical activity in LMIC. Providing an up to date synthesis of the economic evidence base is an efficient way of highlighting current research practices and new findings to inform research practice and the formulation of cost effective physical activity programmes and policies.

Objectives

The review will:

- Describe the methods underpinning the economic evidence base on physical activity in LMIC

For peer review only

- Appraise how these methods are operationalised
- Identify research gaps and provide recommendations for economic research agenda of physical activity
- Identify potential target variables and cost effective interventions for policy formulation

METHODS

The methods for this review were informed by previous reviews of economic analyses of physical activity [10,13-19] and are in line with recommendations on review of economic evidence[20-22]. This will be based on "Preferred Reporting Items for Systematic Review and Meta Analyses Protocols (PRISMA-P)" Statement [23] (supplementary file 1). The protocol will be registered with the PROSPERO International Prospective Register of Systematic Reviews following peer review.

Eligibility criteria

Economic studies on physical activity in LMIC will be included. Specifically, the following study types will be included: (a) economic evaluations of physical activity interventions, (b) economic burden of physical inactivity; (c) cost of physical activity participation, (d) demand for physical activity; (e) and economic correlates of physical (in) activity. Additional selection criteria are:

Inclusion criteria

1. Study design: Observational studies (cohort, case control, cross sectional);experimental studies including randomised controlled trials (RCTs); quasi experimental studies including natural experiments; and economic evaluation studies
2. Study setting: Any setting of LMIC in accordance with the definition of LMIC by World Bank, Classification 2017[4].

For peer review only

3. Population: Any age group across the life course
4. Studies reported only in English language

Exclusion criteria

1. Case reports, case series, letters to the editor, editorials, reviews, qualitative studies unpublished theses, conference abstracts, and any unobtainable texts.
2. Studies published in a language other than English

Search strategy

We will search the following electronic data bases to identify studies: Scopus (covers 100% MEDLINE coverage, 100% of EMBASE coverage and 100% of Compendex coverage [24]), Web of Science; and SPORTDiscus. Websites of WHO, NICE international, World Bank will be searched for relevant studies. The reference lists of included studies will be searched for any relevant articles. Searches will not be restricted by date of the publication to maximize the sensitivity.

The search strategy was informed by a scoping review that covered relevant reviews on economics studies on physical activity [10,13-19],reviews on physical activity[25-27] and reviews on economic evaluations[28-30]. The draft search strategy was then reviewed by subject experts and a subject liaison librarian to optimize the sensitivity and the specificity of the search. Supplementary file 2 shows a sample search strategy.

Study Selection

A two stage approach involving three independent reviewers will be used to select relevant papers.

- At the first stage, two reviewers (PR and NA) will independently screen the titles and abstracts of identified papers. Disagreement will be resolved through discussions with a

For peer review only

third reviewer (SP). We will include a study if in doubt about its inclusion. For example, if the country setting of a study is unclear in the title and abstract, we will take it forward to the next stage.

- At the second stage, the full text of papers of papers selected from stage 1 will be reviewed independently by two reviewers (PR and NA). Selected and excluded papers with reasons will be discussed by the two reviewers and a third reviewer (SP) at a consensus meeting and disagreement will be resolved by real time consensus. Corresponding authors will be contacted via email for clarification if the need be. Study selection process will be presented in a PRISMA flow chart [31] along with the reasons for exclusion of studies.

Data management and extraction

Endnote X5 software will be used to manage the search results. Data extraction will be performed using standardized pilot-tested data extraction form developed based on relevant data extraction forms from relevant reviews [17,32,33]. The adaptation process has involved matching the review objectives with the available data extraction forms. Data from the final selected full text articles will be extracted by one reviewer (PR). To ensure quality of data extraction, a second reviewer (NA) will independently extract data from a random selection of the final articles. Disagreements between two reviewers will be discussed and resolved through discussions with the third reviewer (SP). Any unresolved disagreements will be reported in the final report. Supplementary file 3 shows the draft data extraction form. Data will be extracted for the following items: general information, characteristic of the study, characteristics of population/condition/intervention, data sources/data analysis/results, conclusions and way forward suggested by author, challenges, and quality assessment.

For peer review only

Risk of Bias and quality assessment

Risk of bias will be assessed by a one reviewer (PR) for all selected articles. A second reviewer (NA) will also independently assess the risk of bias on randomly selected articles. The risk of bias assessment questions are designed based on relevant literature in this area [14,17,33-37] (supplementary file 4). Risk of bias will be assessed using following tools;

- For economic evaluation studies, the Drummond checklist [38] which consists of 35 items will be used. If an economic evaluation study is based on a decision analytical model, the Phillips [39] check list will be used in addition.
- For cost of illness studies, the Larg & Moss checklist [40] will be used.
- For studies on correlation or association, we will use the check lists recommended by "The National Institute for Health and Care Excellence" (NICE) [41].

Quality grading will be given to each included study based on overall quality of the study. The grading process will follow the NICE recommendations [34] (Figure 1):

- Good quality (++): all most all check list criteria have been fulfilled, where they have not been fulfilled the conclusions are very unlikely to alter
- Moderate quality (+) : some of the check list criteria are fulfilled, where they have not been fulfilled or not adequately described, conclusions are unlikely to alter
- Low quality (-): few or no checklist criteria are fulfilled, and conclusions are likely or very likely to alter

Data synthesis and reporting

A descriptive synthesis of data will be performed by evaluating characteristics, quality and the results of the studies and this will be presented in tabular format. Where the numbers permit, summaries of studies will be classified according to type of economic analysis, country or region, and

For peer review only

intervention type. Where appropriate, we will conduct a meta-analysis to quantify the effect sizes (e.g. that of economic variables on the demand for physical activity). Such analyses will adjust for between-study heterogeneity using random effects models. The I-squared (I^2) statistic will be used to evaluate the heterogeneity related to the findings of papers. Quantitative analysis will be conducted using STATA 13 software.

We will report the number of studies where data extraction items were not applicable, as an indicator of the quality of reporting. Recommendations for future research and policies will be presented. This review will be reported in accordance with PRISMA 2009 statement [31]. Study selection process will be illustrated by flow diagram including the reasons for exclusion of studies at each stage. Search strategy, Data extraction form and quality assessment tools will be published as supplementary files. This protocol will not be amended and any changes will be described and discussed in final report.

Patient and Public Involvement

Patents and public are not directly involved in this study as original data will not be collected

ETHICS AND DISSEMINATION

Ethical clearance is not required as original data will not be collected. Best practice guidelines to conduct systematic reviews [31] will be followed. Results will be disseminated by peer reviewed publications and conference presentations.

Discussion

For peer review only

Population level of physical inactivity in LMIC is likely to increase giving rise to multitude of public health and economic consequences, including rising healthcare costs due to increased NCD related treatments, increased disability-adjusted life years (DALYs) and productivity losses. Economic research on physical activity from the LMIC perspective should therefore be considered as a priority area. This is because increased physical inactivity could lead to uncontrolled healthcare needs in LMICs where resources (including that for healthcare) are limited and often shrinking in real terms. Pooling of the available research evidence on economics of physical activity in LMIC will thus reveal the current knowledge on economics of physical activity in LMIC, thereby guiding the future of national, regional and local policies around physical activity.

This will be the first study to review the evidence base on economics of physical activity in the context of low and middle income countries which can be considered as the main strength of this study. This will identify gaps in knowledge to support future studies in the area. However, we will include the studies published as peer reviewed articles and published in English language only. Thus, possibility of publication bias cannot be excluded.

World health organization is in the process of drafting "Global action plan on physical activity (GAPPA) to be implemented from 2018-2030" [42]. This is timely as the evidence from this research will provide useful information for implementation and prioritize the actions of GAPPA in LMIC.

The evidence from this research will be useful to policy makers and stakeholders dealing with physical activity promotion at individual, local, national, regional and global level with special reference to LMIC. Further, this review will be an important base for a research

For peer review only

agenda on economics of physical activity in LMIC. This will be a useful guide for researchers to design research on economic and physical activity with sound methodology based on the research need of the LMIC. Further, information gathered from this research will guide funding agencies for effective allocation of resources.

Authors Contributors: All authors conceived the research idea and reviewed the manuscript. NA and SP provided overall technical guidance. PR is the guarantor. PR, NA and SP developed search strategies, methods and data extraction forms. PR conducted the preliminary search and developed the initial draft of the protocol. All authors critically revised and approved the final version of the manuscript.

Funding: This research received no specific grant from any funding agency in the public, commercial or not-for-profit sectors. PR is a post-doctoral fellow supported by Ministry of Health, Sri Lanka. NA and SP are employees of Brunel University London.

Conflicts of interest: None

Data sharing statement: All data for this manuscript included with the submission

For peer review only

Supplementary files

1. Comparing the study protocol with PRISMA-P 2015 check list and location in the manuscript
2. Proposed search strategy for the review (sample)
3. Data extraction form illustrating the items for which the data will be extracted and check lists for risk of bias assessment

4. Summary of tools used for risk of bias assessment of relevant literature and basis for the plan of risk of bias assessment in this review

References

1. Global Recommendations on Physical Activity for Health. Geneva: World Health Organization; 2010.Available from: https://www.ncbi.nlm.nih.gov/ books/NBK 305057/

2. World Health Organization 2017, *Fact Sheet – Media centre: Physical activity, viewed on* 6 February 2018, <http://www.who.int/mediacentre/factsheets/fs385/en/ >

3. Murray, C. J., Barber, R. M., Foreman, K. J., Abbasoglu Ozgoren, A., Abd-Allah, F., Abera, S. F., Vos, T. (2015). Global, regional, and national disability-adjusted life years (DALYs) for 306 diseases and injuries and healthy life expectancy (HALE) for 188 countries, 1990-2013: quantifying the epidemiological transition. [Research Support, Non-U S Gov't]. *Lancet, 386*(10009), 2145-2191.

4. World Bank Country and Lending Groups: Country classification (June 2017). https://datahelpdesk.worldbank.org/knowledgebase/articles/906519-world-bank-country-and-lending-groups>

5. Abegunde, D. O., Mathers, C. D., Adam, T., Ortegon, M., & Strong, K. (2007). The burden and costs of chronic diseases in low-income and middle-income countries. [Review]. *Lancet, 370*(9603), 1929-1938.

For peer review only

6. Hallal, P. C., Andersen, L. B., Bull, F. C., Guthold, R., Haskell, W., & Ekelund, U. (2012). Global physical activity levels: surveillance progress, pitfalls, and prospects. *The Lancet, 380*(9838), 247-257. doi: 10.1016/s0140-6736(12)60646-1

7. WHO. 2015. Prevalence of insufficient physical activity among adults Data by World Bank income groups. Accessed at http://apps.who.int/gho/data/view.main.2487?lang=en

8. WHO.2009.Global health risks: mortality and burden of disease attributable to selected major risks. ISBN 978 92 4 156387 1. Accessed at http://www.who.int/healthinfo/global_burden_disease/GlobalHealthRisks_report_full.pdf

9. Atkinson, K., Lowe, S., & Moore, S. (2016). Human development, occupational structure and physical inactivity among 47 low and middle income countries. *Preventive Medicine Reports, 3*, 40-45. doi: https://doi.org/10.1016/j.pmedr.2015.11.009

10. Oldridge, N. B. (2008). Economic burden of physical inactivity: healthcare costs associated with cardiovascular disease. *European Journal of Cardiovascular Prevention & Rehabilitation, 15*(2), 130-139. doi: 10.1097/HJR.0b013e3282f19d42

11. Islam, S. M. S., Purnat, T. D., Phuong, N. T. A., Mwingira, U., Schacht, K., & Fröschl, G. (2014). Non-Communicable Diseases (NCDs) in developing countries: a symposium report. *Globalization and Health*, *10*, 81. http://doi.org/10.1186/s12992-014-0081-9

12. Sturm, R. (2005). Economics and physical activity - A research agenda. *American Journal of Preventive Medicine, 28*(2), 141-149. doi: 10.1016/j.amepre.2004.10.021

13. Jeroen C. M. Barte & G. C. Wanda Wendel-Vos (2017) A Systematic Review of Financial Incentives for Physical Activity: The Effects on Physical Activity and Related Outcomes, Behavioral Medicine, 43:2, 79-90, DOI: 10.1080/08964289.2015.1074880

14. Abu-Omar, K., Rütten, A., Burlacu, I., Schätzlein, V., Messing, S., & Suhrcke, M. (2017). The cost-effectiveness of physical activity interventions: A systematic review of reviews. *Preventive Medicine Reports*, *8*, 72–78. http://doi.org/10.1016/j.pmedr.2017.08.006

15. Brown, V., Zapata Diomedi, B., Moodie, M., Veerman, J., & Carter, R. (2016). *A systematic review of economic analyses of active transport interventions that include physical activity benefits* (Vol. 45).

16. Ding, D., Kolbe-Alexander, T., Nguyen, B., Katzmarzyk, P. T., Pratt, M., & Lawson, K. D. (2017). The economic burden of physical inactivity: A systematic review and critical appraisal. [Review]. British Journal of Sports Medicine, 51(19), 1392-1409. doi: 10.1136/bjsports-2016-097385

17. Pavey, T. G., Taylor, A. H., Fox, K. R., Hillsdon, M., Anokye, N., Campbell, J. L., Taylor, R. S. (2011). Effect of exercise referral schemes in primary care on physical activity and improving health outcomes: systematic review and meta-analysis. *BMJ, 343*. doi: 10.1136/bmj.d6462

18. Campbell, F., Holmes, M., Everson-Hock, E., Davis, S., Woods, H. B., Anokye, N., Kaltenthaler, E. (2015). A systematic review and economic evaluation of exercise referral schemes in primary care: a short report. *Health Technology Assessment, 19*(60), 1-+. doi: 10.3310/hta19600

For peer review only

19. Suhrcke, M., Boluarte, T. A., & Niessen, L. (2012). A systematic review of economic evaluations of interventions to tackle cardiovascular disease in low- and middle-income countries. [journal article]. *BMC Public Health, 12*(1), 2. doi: 10.1186/1471-2458-12-2

20. Kaltenthaler, E., Tappenden, P., & Paisley, S. (2013). Reviewing the evidence to inform the population of cost-effectiveness models within health technology assessments. [Research Support, Non-U S Gov't]. *Value Health, 16*(5), 830-836.

21. Zechmeister-Koss, I., Schnell-Inderst, P., & Zauner, G. (2014). Appropriate evidence sources for populating decision analytic models within health technology assessment (HTA): a systematic review of HTA manuals and health economic guidelines. [Review]. *Med Decis Making, 34*(3), 288-299.

22. NICE (2012). The guideline manual Process and methods (PMG60) Published date: November 2012

23. Moher, D., Shamseer, L., Clarke, M., Ghersi, D., Liberati, A., Petticrew, M., Stewart, L. A. (2015). Preferred reporting items for systematic review and meta-analysis protocols (PRISMA-P) 2015 statement. [Research Support, Non-U S Gov't Research Support, U S Gov't, P H S]. *Syst Rev, 4*(1), 2046-4053.

24. Burnham, J. F. (2006). Scopus database: a review. *Biomed Digit Libr, 3*(1), 1742-5581.

25. Milton, K., Macniven, R., & Bauman, A. (2014). Review of the epidemiological evidence for physical activity and health from low- and middle-income countries. [Review]. *Glob Public Health, 9*(4), 369-381.

For peer review only

26. Lachat, C., Otchere, S., Roberfroid, D., Abdulai, A., Seret, F. M., Milesevic, J., Kolsteren, P. (2013). Diet and physical activity for the prevention of noncommunicable diseases in low- and middle-income countries: a systematic policy review. [Meta-Analysis Review]. *PLoS Med, 10*(6), 11.

27. Hunter, R. F., Christian, H., Veitch, J., Astell-Burt, T., Hipp, J. A., & Schipperijn, J. (2015). The impact of interventions to promote physical activity in urban green space: a systematic review and recommendations for future research. [Research Support, Non-U S Gov't Review]. *Soc Sci Med, 124*, 246-256.

28. Sutton, L., Karan, A., & Mahal, A. (2014). Evidence for cost-effectiveness of lifestyle primary preventions for cardiovascular disease in the Asia-Pacific Region: a systematic review. *Globalization and Health*, *10*, 79. http://doi.org/10.1186/s12992-014-0079-3

29. Santatiwongchai, B., Chantarastapornchit, V., Wilkinson, T., Thiboonboon, K., Rattanavipapong, W., Walker, D. G., Teerawattananon, Y. (2015). Methodological Variation in Economic Evaluations Conducted in Low- and Middle-Income Countries: Information for Reference Case Development. *PLoS ONE, 10*(5), e0123853. doi: 10.1371/journal.pone.0123853

30. Nosratnejad, S., Rashidian, A., & Dror, D. M. (2016). Systematic Review of Willingness to Pay for Health Insurance in Low and Middle Income Countries. [Review]. *PLoS One, 11*(6).

31. Moher, D., Liberati, A., Tetzlaff, J., & Altman, D. G. (2009). Preferred reporting items for systematic reviews and meta-analyses: the PRISMA statement. [Guideline Research Support, Non-U S Gov't]. *J Clin Epidemiol, 62*(10), 1006-1012.

For peer review only

32. Pokhrel, S., Quigley, M. A., Fox-Rushby, J., McCormick, F., Williams, A., Trueman, P., Renfrew, M. J. (2015). Potential economic impacts from improving breastfeeding rates in the UK. [Research Support, Non-U.S. Gov't]. *Arch Dis Child, 100*(4), 334-340. doi: 10.1136/archdischild-2014-306701

33. Murthy, S., John, D., Godinho, I. P., Godinho, M. A., Guddattu, V., & Nair, N. S. (2017). A protocol for a systematic review of economic evaluation studies conducted on neonatal systemic infections in South Asia. *Syst Rev, 6*(1), 252. doi: 10.1186/s13643-017-0648-7

34. Aminde, L. N., & Veerman, L. (2016). Interventions for the prevention of cardiovascular diseases: a protocol for a systematic review of economic evaluations in low-income and middle-income countries. *BMJ Open, 6*(12), e013668. doi: 10.1136/bmjopen-2016-013668

35. Ruifrok, A. E., Rogozinska, E., van Poppel, M. N., Rayanagoudar, G., Kerry, S., de Groot, C. J., Thangaratinam, S. (2014). Study protocol: differential effects of diet and physical activity based interventions in pregnancy on maternal and fetal outcomes—individual patient data (IPD) meta-analysis and health economic evaluation. [journal article]. *Systematic Reviews, 3*(1), 131. doi: 10.1186/2046-4053-3-131

36. GC, V., Wilson, E. C., Suhrcke, M., Hardeman, W., & Sutton, S. (2015). Are brief interventions to increase physical activity cost-effective? A systematic review. *British Journal of Sports Medicine*. doi: 10.1136/bjsports-2015-094655

For peer review only

37. Tonmukayakul, U., Calache, H.,Clark, R., Wasiak, J., Faggion Jr, C.M. (2015). *Systematic review and quality appraisal of economic evaluation publications in Dentistry.* Journal of Dental research,94(10), 1348-1354 <https://doi.org/10.1177/0022034515589958>

38. Drummond, M. F., & Jefferson, T. O. (1996). Guidelines for authors and peer reviewers of economic submissions to the BMJ. *BMJ, 313*(7052), 275-283. doi: 10.1136/bmj.313.7052.275

39. Philips, Z., Ginnelly, L., Sculpher, M., Claxton, K., Golder, S., Riemsma, R., Glanville, J. (2004). Review of guidelines for good practice in decision-analytic modelling in health technology assessment. [Review]. *Health Technol Assess, 8*(36), 1-158.

40. Larg, A., & Moss, J. R. (2011). Cost-of-illness studies: a guide to critical evaluation. [Research Support, Non-U S Gov't]. *Pharmacoeconomics, 29*(8), 653-671.

41. National Institute for Health and Excellence (NICE), 2012. Methods for the development of NICE public health guidance (third edition), Appendix G Quality appraisal checklist-quantitative studies reporting correlations and associations.< https://www.nice.org.uk/process/pmg4/chapter/appendix-g-quality-appraisal-checklist-quantitative-studies-reporting-correlations-and >

42. World Health Organization (WHO), 2018. Non communicable diseases and their risk factors, Governance: Development of draft global action plan to promote physical activity. Accessed at < http://www.who.int/ncds/governance/physical_activity_plan/en/>

For peer review only

Figure 1: Economics of PA in LMIC- Plan for data extraction and quality assessment process

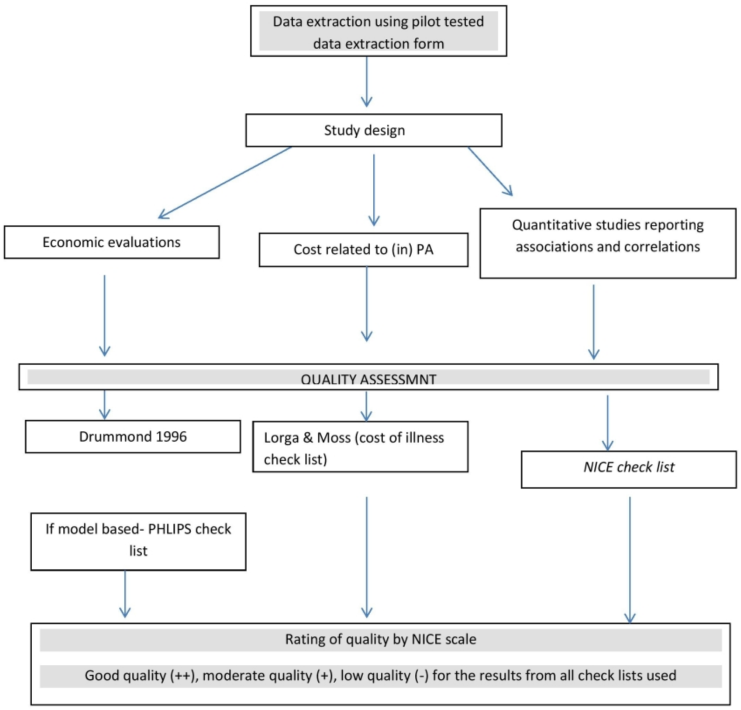

Figure 1: Economics of PA in LMIC- Plan for data extraction and quality assessment process

210x297mm (300 x 300 DPI)

PRISMA-P 2015 Checklist

This checklist has been adapted for use with systematic review protocol submissions to BioMed Central journals from Table 3 in Moher D et al: Preferred reporting items for systematic review and meta-analysis protocols (PRISMA-P) 2015 statement. *Systematic Reviews* 2015 **4**:1

Section/topic	#	Checklist item	Information reported		Line number(s)
			Yes	No	
**ADMINISTRATIVE INFORMATION**					
**Title**					
Identification	1a	Identify the report as a protocol of a systematic review	☒	☐	2
Update	1b	If the protocol is for an update of a previous systematic review, identify as such	☐	☒	
**Registration**	2	If registered, provide the name of the registry (e.g., PROSPERO) and registration number in the Abstract	☐	☒	40-41, 95-96
**Authors**					
Contact	3a	Provide name, institutional affiliation, and e-mail address of all protocol authors; provide physical mailing address of corresponding author	☒	☐	4-16
Contributions	3b	Describe contributions of protocol authors and identify the guarantor of the review	☒	☐	219-223
**Amendments**	4	If the protocol represents an amendment of a previously completed or published protocol, identify as such and list changes; otherwise, state plan for documenting important protocol amendments	☒	☐	184-185
**Support**					
Sources	5a	Indicate sources of financial or other support for the review	☒	☐	224-226
Sponsor	5b	Provide name for the review funder and/or sponsor	☐	☒	
Role of sponsor/funder	5c	Describe roles of funder(s), sponsor(s), and/or institution(s), if any, in developing the protocol	☐	☒	
**INTRODUCTION**					
**Rationale**	6	Describe the rationale for the review in the context of what is already known	☒	☐	54-83
**Objectives**	7	Provide an explicit statement of the question(s) the review will address with reference to participants, interventions, comparators, and outcomes (PICO)	☒	☐	84-90

For peer review only

BioMed Central The Open Access Publisher

Section/topic	#	Checklist item	Information reported		Line number(s)
			Yes	No	
**METHODS**					
**Eligibility criteria**	8	Specify the study characteristics (e.g., PICO, study design, setting, time frame) and report characteristics (e.g., years considered, language, publication status) to be used as criteria for eligibility for the review	☒	☐	97-113
**Information sources**	9	Describe all intended information sources (e.g., electronic databases, contact with study authors, trial registers, or other grey literature sources) with planned dates of coverage	☒	☐	114-124
**Search strategy**	10	Present draft of search strategy to be used for at least one electronic database, including planned limits, such that it could be repeated	☐	☒	122-124 Supplementary file 2
STUDY RECORDS					
Data management	11a	Describe the mechanism(s) that will be used to manage records and data throughout the review	☒	☐	138-139
Selection process	11b	State the process that will be used for selecting studies (e.g., two independent reviewers) through each phase of the review (i.e., screening, eligibility, and inclusion in meta-analysis)	☒	☐	125-137
Data collection process	11c	Describe planned method of extracting data from reports (e.g., piloting forms, done independently, in duplicate), any processes for obtaining and confirming data from investigators	☒	☐	138-150
**Data items**	12	List and define all variables for which data will be sought (e.g., PICO items, funding sources), any pre-planned data assumptions and simplifications	☒	☐	146-150, Supplementary file 3
**Outcomes and prioritization**	13	List and define all outcomes for which data will be sought, including prioritization of main and additional outcomes, with rationale	☒	☐	Supplementary file 3
**Risk of bias in individual studies**	14	Describe anticipated methods for assessing risk of bias of individual studies, including whether this will be done at the outcome or study level, or both; state how this information will be used in data synthesis	☒	☐	151-169 Supplementary file 3
DATA					
**Synthesis**	15a	Describe criteria under which study data will be quantitatively synthesized	☐	☒	
	15b	If data are appropriate for quantitative synthesis, describe planned summary measures, methods of handling data, and methods of combining data from studies, including any planned exploration of consistency (e.g., I^2, Kendall's tau)	☒	☐	174-178
	15c	Describe any proposed additional analyses (e.g., sensitivity or subgroup analyses, meta-regression)	☒	☐	175-177

For peer review only

Section/topic	#	Checklist item	Information reported		Line number(s)
			Yes	No	
	15d	If quantitative synthesis is not appropriate, describe the type of summary planned	☒	☐	171-172
**Meta-bias(es)**	16	Specify any planned assessment of meta-bias(es) (e.g., publication bias across studies, selective reporting within studies)	☐	☒	
**Confidence in cumulative evidence**	17	Describe how the strength of the body of evidence will be assessed (e.g., GRADE)	☒	☐	162-169

For peer review only

BioMed Central
The Open Access Publisher

Supplementary file 2: Proposed search strategy for Scopus

	Proposed preliminary search strategy for Scopus
#1	Domain 1: Physical activity<br><br>*“physical activity"* OR *"physical exercise"* OR *"physical inactivity"* OR *"physical fitness"* OR *"Sedentary"* OR “*active lifestyle*” OR “active *living*” OR “active *leisure*” OR “active *play*” OR “active *recreation*” OR “active *commuting"*
#2	Domain 2: Economics<br><br>*"economics"* OR *"economic evaluation"* OR *"economic burden"* OR *"health care cost"* OR “*cost analyses*” OR “cost *effectiveness*” OR “cost *benefit*” OR “cost *minimization*” OR “cost *utility*” OR “cost *saving*” OR “cost *outcome*” OR “cost *consequence*”<br><br>OR *"demand"* OR *"supply"* OR *"market"* OR *"price"* OR *"incentives"* OR *"willingness to pay"* OR *"time use"*
#3	#1 AND #2

For peer review only

Supplementary file 3

DRAFT DATA EXTRACTION FORM FOR REVIEW ON ECONONOMICS AND PYSICAL ACTIVITY IN LOW AND MIDDLE INCOME COUNTRIES (ADAPTED FROM MURTHEY ET AL., 2017, POKHREL ET AL., 2014, PAVEY ET AL., 2011)			
No.	Item category	Items	Notes
1	General information	1.1 Study ID<br>1.2 Review author ID<br>1.3 Date of review<br>1.4 Title, authors(s)<br>1.5 Year of publication<br>1.6 Country studies<br>1.7 Income level of the country	
2	Characteristics of the study	2.1 Aims and objectives/research question<br>2.2. Eligibility decision/Inclusion and exclusion criteria (?)<br>2.3 Study design<br>2.4 Study setting<br>2.5 Total study duration<br>2.6 Sample size<br>2.7 Sample size calculation<br>2.8 Recruitment<br>2.9 Sample selection<br>2.10 Method of allocation<br>2.11 Perspective (i.e. Societal, health care system, service provider etc.)	
3	Characteristics of the population/condition /intervention	**Population/participants**<br>3.1 Type of population<br>(i.e. General population university students, group of employees)<br>3.2 Age<br>3.3 Gender<br>3.4 Ethnicity<br>**Condition**<br>3.5 Definition of physical activity /physical inactivity/sedentary	

		3.6Definition of disease condition<br>**Intervention**<br>3.7 Type of physical activity/programme<br>3.9 Comparator	
4	Data sources/Data analysis/results	**Scope**<br>4.1 Type of analysis<br>(Ie. Costing/economic evaluation/association of economic factors with physical activity/other)<br>4.2 Form of economic evaluation<br>4.3 Time horizon<br>4.4 Study assumptions<br>**Cost**<br>4.5 Reported costs/cost components<br>4.6 Data sources for cost measures<br>4.7 How cost was reported<br>(i.e. marginal cost/ average cost/incremental cost/total cost/other)<br>4.8 Discount rate<br>4.9 Year of costing<br>4.10 Currency unit<br>4.11 Currency conversion rate<br>4.12 Inflation adjustments<br>**Outcomes**<br>4.13 Reported outcomes<br>(i.e. measures of effectiveness/efficacy/measures of effect)<br>4.14 Data sources for the outcome measures<br>4.15 Discount rate<br>**Sensitivity analysis**<br>4.16 Performed/ not<br>4.17 Type of analysis<br>(i.e. deterministic/ stochastic)<br>4.18 Variables used for sensitivity analysis<br>4.19 Results of sensitivity analysis<br>**Main results**	

For peer review only

		4.20 Cost / cost effectiveness<br>4.21 Other	
5	Conclusions and way forward	5.1 Key Conclusion(s)<br>5.2 Suggestions by author for further research	
6	Challenges	6.1 Author stated limitations<br>6.2 Author stated strengths<br>6.3 Miscellaneous comments from author<br>6.4 Funding source<br>6.5 Conflicts of interest	
7.	Quality assessment of studies	7.1 Economic evaluation studies- Drummond checklist<br>7.2 Model based economic evaluations-Phillips check list<br>7.3 Cost of illness/cost of risk factor study/cost estimation- check list by Larg and Moss<br>7.4 Quantitative study reporting correlation or association? NICE check list for quantitative studies reporting correlations and associations	

**Method of rating the quality of studies from each check list (A, B, C, D) in accordance to the quality appraisal by NICE scale (Methods for the development of NICE public health guidance (third edition))**	
• Reviewer is requested to complete in detail the comment section of quality appraisal check lists so that the grade awarded for each study aspect is as transparent as possible<br>• At the end of completion of the check list each study is awarded an overall quality grading for internal validity (IV)and a separate one for external validity (EV)	
All or most of the checklist criteria have been fulfilled, where they have not been fulfilled the conclusions are very unlikely to alter	++
Some of the checklist criteria have been fulfilled, where they have not been fulfilled, or not adequately described, the conclusions are unlikely to alter	+
Few or no checklist criteria have been fulfilled and the conclusions are likely or very likely to alter.	-

For peer review only

CHECKLIST A

QUALITY ASSESSMENT FOR ECONOMIC EVALUATION(CHECKLIST FROM DRUMMOND AND JEFFERSON 1996)	Yes	No	Not clear	Not appropriate
**Study design**				
1) The research question is stated				
2) The economic importance of the research question is stated				
3) The viewpoint(s) of the analysis are clearly stated and justified				
4) The rationale for choosing the alternative programmes orb interventions compared is stated				
5) The alternatives being compared are clearly described				
6) The form of economic evaluation used is stated				
7) The choice of form of economic evaluation is justified in relation to the questions addressed				
**Data collection**				
8) The source(s) of effectiveness estimates used are stated				
9) Details of the design and results of effectiveness study are given (if based on a single study)				
10) Details of the method of synthesis or meta-analysis of estimates are given (if based on an overview of a number				

For peer review only

QUALITY ASSESSMENT FOR ECONOMIC EVALUATION(CHECKLIST FROM DRUMMOND AND JEFFERSON 1996)	Yes	No	Not clear	Not appropriate
of effectiveness studies)				
11) The primary outcome measure(s) for the economic evaluation are clearly stated				
12) Methods to value health states and other benefits are stated				
13) Details of the subjects from whom valuations were obtained are given				
14) Productivity changes (if included) are reported separately				
15) The relevance of productivity changes to the study question is discussed				
16) Quantities of resources are reported separately from their unit costs				
17) Methods for the estimation of quantities and unit costs are described				
18) Currency and price data are recorded				
19) Details of currency of price adjustments for inflation or currency conversion are given				
20) Details of any model used are given				
21) The choice of model used and the key parameters on which it is based are justified				
**Analysis and interpretation of results**				
22) Time horizon of costs and benefits is stated				
23) The discount rate(s) is stated				

For peer review only

QUALITY ASSESSMENT FOR ECONOMIC EVALUATION(CHECKLIST FROM DRUMMOND AND JEFFERSON 1996)	Yes	No	Not clear	Not appropriate
24) The choice of rate(s) is justified				
25) An explanation is given if costs or benefits are not discounted				
26) Details of statistical tests and confidence intervals are given for stochastic data				
27) The approach to sensitivity analysis is given				
28) The choice of variables for sensitivity analysis is justified				
29) The ranges over which the variables are varied are stated				
30) Relevant alternatives are compared				
31) Incremental analysis is reported				
32) Major outcomes are presented in a disaggregated as well as aggregated form				
33) The answer to the study question is given				
34) Conclusions follow from the data reported				
35) Conclusions are accompanied by the appropriate caveats				

Did the economic evaluation use a decision-analytic modelling framework?	Response: Yes(✓), No (×) , Not applicable (NA)<br><br>Instruction: If *Yes*, assess paper using the questions in Check list B, If *No/NA*, skip check list B

For peer review only

CHECKLIST B

QUALITY ASSESSMENT FOR DECISION-ANALYTIC MODELS(CHECKLIST FROM PHILIPS ET AL. 2004)			
**Quality criterion**	**Question(s)**	**Response (✓, ✗, NA)**	**Comments**
S1	Is there a clear statement of the decision problem?		
	Is the objective of the evaluation and model specified and consistent with the stated decision problem?		
	Is the primary decision maker specified?		
S2	Is the perspective of the model stated clearly?		
	Are the model inputs consistent with the stated perspective?		
	Has the scope of the model been stated and justified?		
	Are the outcomes of the model consistent with the perspective, scope and overall objective of the model?		
S3	Is the structure of the model consistent with a coherent theory of the health condition under evaluation?		
	Are the sources of data used to develop the structure of the model specified?		

For peer review only

QUALITY ASSESSMENT FOR DECISION-ANALYTIC MODELS(CHECKLIST FROM PHILIPS ET AL. 2004)			
**Quality criterion**	**Question(s)**	**Response (✓, ✗, NA)**	**Comments**
	Are the causal relationships described by the model structure justified appropriately?		
S4	Are the structural assumptions transparent and justified?		
	Are the structural assumptions reasonable given the overall objective, perspective and scope of the model?		
S5	Is there a clear definition of the options under evaluation?		
	Have all feasible and practical options been evaluated?		
	Is there justification for the exclusion for the exclusion of feasible options?		
S6	Is the chosen model type appropriate given the decision problem and specified causal relationships within the model?		
S7	Is the time horizon of the model sufficient to reflect all important differences between options?		
	Are the time horizon of the model, the duration of treatment and the duration of treatment effect described and justified?		
S8	Do the disease states (state transition model) or the pathways (decision tree model) reflect the underlying biological process of the		

For peer review only

**QUALITY ASSESSMENT FOR DECISION-ANALYTIC MODELS(CHECKLIST FROM PHILIPS ET AL. 2004)**			
**Quality criterion**	**Question(s)**	**Response (✓, ✗, NA)**	**Comments**
	disease in question and the impact of interventions?		
S9	Is the cycle length defined and justified in terms of natural history of disease?		
D1	Are the data identification methods transparent and appropriate given the objectives of the model?		
	Where choices have been made between data sources, are these justified appropriately?		
	Has particular attention been paid to identifying data for the important parameters in the model?		
	Has the quality of the data been assessed appropriately?		
	Where expert opinion has been used, are the methods described and justified?		
D2	Is the data modelling methodology based on justifiable statistical and epidemiological techniques?		
D2a	Is the choice of baseline data described and justified?		
	Are transition probabilities calculated appropriately?		

For peer review only

QUALITY ASSESSMENT FOR DECISION-ANALYTIC MODELS(CHECKLIST FROM PHILIPS ET AL. 2004)			
**Quality criterion**	**Question(s)**	**Response (✓, ✗, NA)**	**Comments**
	Has a half-cycle correction been applied to both cost and outcome?		
	If not, has this omission been justified?		
D2b	If relative treatment effects have been derived from trail data, have they been synthesised using appropriate techniques?		
	Have the methods and assumptions used to extrapolate short-term results to final outcomes been documented and justified?		
	Have alternative extrapolation assumptions been explored through sensitivity analysis?		
	Have assumptions regarding the continuing effect of treatment once treatment is complete been documented and justified?		
	Have alternative assumptions regarding the continuing effect of treatment been explored through sensitivity analysis?		
D2c	Are the costs incorporated into the model justified?		
	Has the source for all costs been described?		
	Have discount rates been described and justified given the target decision-maker?		

For peer review only

QUALITY ASSESSMENT FOR DECISION-ANALYTIC MODELS(CHECKLIST FROM PHILIPS ET AL. 2004)			
**Quality criterion**	**Question(s)**	**Response (✓, ✗, NA)**	**Comments**
D2d	Are the utilities incorporated into the model appropriate?		
	Is the source for the utility weights referenced?		
	Are the methods of derivation for the utility weights justified?		
D3	Have all data incorporated into the model been described and referenced in sufficient detail?		
	Has the use of mutually inconsistent data been justified (i.e. are assumptions and choices appropriate)?		
	Is the process of data incorporation transparent?		
	If data have been incorporated as distributions, has the choice of distribution for each parameter been described and justified?		
	If data have been incorporated as distributions, is it clear that second order uncertainty is reflected?		
D4	Have the four principal types of uncertainty been addressed?		
	If not, has the omission of particular forms of uncertainty been justified?		
D4a	Have methodological uncertainties been addressed by running alternative versions of the model with different methodological		

For peer review only

**QUALITY ASSESSMENT FOR DECISION-ANALYTIC MODELS(CHECKLIST FROM PHILIPS ET AL. 2004)**			
**Quality criterion**	**Question(s)**	**Response (✓, ✗, NA)**	**Comments**
	assumptions?		
D4b	Is there evidence that structural uncertainties have been addressed via sensitivity analysis?		
D4c	Has heterogeneity been dealt with by running the model separately for different subgroups?		
D4d	Are the methods of assessment of parameter uncertainty appropriate?		
	If data are incorporated at point estimates, are the ranges used for sensitivity analysis stated clearly and justified?		
C1	Is there evidence that the mathematical logic of the model has been tested thoroughly before use?		
C2	Are any counterintuitive results from the model explained and justified?		
	If the model has been calibrated against independent data, has any differences been explained and justified?		
	Have the results of the model been compared with those of previous models and any differences in results explained?		

For peer review only

CHECKLIST C

QUALITY ASSESSMNET FOR COST OF ILLNESS STUDIES (ADAPTED FROM LARG AND MOSS, 2011)			
Responses (marked Similar to NICE check list, write the answer where relevant under the comment section)<br><br>++ Indicates that for that particular aspect of study design, the study has been designed or conducted in such a way as to minimise the risk of bias.<br>+ Indicates that either the answer to the checklist question is not clear from the way the study is reported, or that the study may not have addressed all potential sources of bias for that particular aspect of study design.<br>- Should be reserved for those aspects of the study design in which significant sources of bias may persist.<br>NR (Not Reported) - Should be reserved for those aspects in which the study under review fails to report how they have (or might have) been considered.<br>NA (Not Applicable) Should be reserved for those study design aspects that are not applicable given the study design under review (for example, allocation concealment would not be applicable for case–control studies).			
	Question	Response<br>***++/+/-NR/NA***	Comment
1.	Analytical framework: what costs should have been measured?<br><br>(a) What was the motivation and perspective of the study?<br><br>(b) Was the appropriate epidemiologic approach taken?<br><br>(c) Was the study question well specified?<br>(i) Were all relevant, non-trivial cost components and their stakeholders identified?<br>(ii) Were necessary timeframes specified?<br>(iii) Was a case of disease or risk factor adequately and appropriately defined?<br>(iv) Was the counterfactual population occurrence plausible and meaningful?		
2.	(2) Methodology and data: how well were resource use and productivity losses measured?		

For peer review only

	(a) Was an appropriate method(s) of quantification used, such that;<br>(i) Additional, or excess, costs were measured?<br>(ii) only costs specific to (caused by) the health problem were included (confounders controlled)?<br>(iii) All important effects were captured?<br>(iv) Important differences across subpopulations were accounted for?<br>(v) The required level of detail could be provided?<br><br>(b) Was the resource quantification method(s) well executed?<br>(i) For population-based studies, were cost allocation methods, data and assumptions valid?<br>(ii) For person-based studies, were appropriate statistical tests performed and reported?<br>(iii) Were data representative of the study population?<br>(iv) Were there any other relevant resource quantification issues?<br><br>(c) Were healthcare resources valued appropriately?<br><br>(d) Was the approach for valuing production losses justified, and assumptions valid?<br><br>(e) Was the inclusion of intangible costs appropriate:<br>(i) Was double counting of mortality-related production losses avoided?<br>(ii) Were losses valued appropriately, given the study's perspective?		
3	Analysis and reporting<br>(a) Did the analysis address the study question?<br><br>(b) Was a range of estimates presented?		

For peer review only

For peer review only - http://bmjopen.bmj.com/site/about/guidelines.xhtml

	(c) Were the main uncertainties identified?<br><br>(d) Was a sensitivity analysis performed on:<br><br>(i) important (uncertain) parameter estimates?<br>(ii) key assumptions? (including the counterfactual)<br>(iii) point estimates? (based on confidence or credible intervals)<br><br>(e) Was adequate documentation and justification given for cost components, data and sources, assumptions and methods?<br><br>(f) Was uncertainty around the estimates and its implications adequately discussed?<br><br>(g) Were important limitations discussed regarding the cost components, data, assumptions and methods?<br><br>(h) Were the results presented at the appropriate level of detail to answer the study question (cost components; disease subtypes, severity, stage; subpopulation groups, cost bearers)?		

For peer review only

CHECKLIST D

QUALITY ASSESSMENT FOR QUANTITATIVE STUDIES REPORTING CORRELATIONS AND ASSOCIATIONS(NICE,2012 BASED ON 'GRAPHICAL APPRAISAL TOOL FOR EPIDEMIOLOGICAL STUDIES(GATE)` BY JACKSON ET AL. (2006)			
Responses:<br><br>++ Indicates that for that particular aspect of study design, the study has been designed or conducted in such a way as to minimise the risk of bias.<br><br>+ Indicates that either the answer to the checklist question is not clear from the way the study is reported, or that the study may not have addressed all potential sources of bias for that particular aspect of study design.<br><br>- Should be reserved for those aspects of the study design in which significant sources of bias may persist.<br><br>NR (Not Reported) - Should be reserved for those aspects in which the study under review fails to report how they have (or might have) been considered.<br><br>NA (Not Applicable) Should be reserved for those study design aspects that are not applicable given the study design under review (for example, allocation concealment would not be applicable for case–control studies).			
**Section 1: Population**			
**Number**	**Question**	**Response**<br><br>**++/+/-/ NR/NA**	**Comments**
1.1	Is the source population or source area well described?<br><br>Was the country (e.g. developed or non-developed, type of health care system), setting (primary schools, community centres etc), location (urban, rural), population demographics etc adequately described?		

For peer review only

1.2	Is the eligible population or area representative of the source population or area?<br><br>Was the recruitment of individuals, clusters or areas well defined (e.g. advertisement, birth register)?<br><br>Was the eligible population representative of the source? Were important groups underrepresented?		
1.3	Do the selected participants or areas represent the eligible population or area?<br><br>Was the method of selection of participants from the eligible population well described?<br><br>What % of selected individuals or clusters agreed to participate? Were there any sources of bias?<br><br>Were the inclusion or exclusion criteria explicit and appropriate?		
**Section 2: Method of selection of exposure (or comparison) group**			
2.1	Selection of exposure (and comparison) group. How was selection bias minimised?<br><br>How was selection bias minimised?		
2.2	Was the selection of explanatory variables based on a sound theoretical basis?<br><br>How sound was the theoretical basis for selecting the explanatory variables?		
2.3	Was the contamination acceptably low?<br><br>Did any in the comparison group receive the exposure?		

For peer review only

	If so, was it sufficient to cause important bias?		
2.4	How well were likely confounding factors identified and controlled?<br><br>Were there likely to be other confounding factors not considered or appropriately adjusted for?<br><br>Was this sufficient to cause important bias?		
**Section 3: Outcomes**			
3.1	Were the outcome measures and procedures reliable?<br><br>Were outcome measures subjective or objective (e.g. biochemically validated nicotine levels ++ vs self-reported smoking −)?<br><br>How reliable were outcome measures (e.g. inter- or intra-rater reliability scores)?<br><br>Was there any indication that measures had been validated (e.g. validated against a gold standard measure or assessed for content validity)?		
3.2	Were the outcome measurements complete?<br><br>Were all or most of the study participants who met the defined study outcome definitions likely to have been identified?		
3.3	Were the outcome measurements complete?<br><br>Were all or most of the study participants who met the defined study outcome definitions likely to have been identified?		

For peer review only

3.4	Was there a similar follow-up time in exposure and comparison groups?<br><br>If groups are followed for different lengths of time, then more events are likely to occur in the group followed-up for longer distorting the comparison.<br><br>Analyses can be adjusted to allow for differences in length of follow-up (e.g. using person-years).		
3.5	Was follow-up time meaningful?<br><br>Was follow-up long enough to assess long-term benefits and harms?<br><br>Was it too long, e.g. participants lost to follow-up?		
**Section 4: Analyses**			
4.1	Was the study sufficiently powered to detect an intervention effect (if one exists)?<br><br>A power of 0.8 (i.e. it is likely to see an effect of a given size if one exists, 80% of the time) is the conventionally accepted standard.<br><br>Is a power calculation presented? If not, what is the expected effect size? Is the sample size adequate?		
4.2	Were multiple explanatory variables considered in the analyses?<br><br>Were there sufficient explanatory variables considered in the analysis?		
4.3	Were the analytical methods appropriate?<br><br>Were important differences in follow-up time and likely confounders adjusted		

For peer review only

	for?		
4.4	Was the precision of association given or calculable? Is association meaningful?<br><br>Were confidence intervals or p values for effect estimates given or possible to calculate?<br><br>Were CIs wide or were they sufficiently precise to aid decision-making? If precision is lacking, is this because the study is under-powered?		
**Section 5: Summary**			
5.1	Are the study results internally valid (i.e. unbiased)?<br><br>How well did the study minimise sources of bias (i.e. adjusting for potential confounders)?<br><br>Were there significant flaws in the study design?		
5.2	Are the findings generalizable to the source population (i.e. externally valid)?<br><br>Are there sufficient details given about the study to determine if the findings are generalizable to the source population?<br><br>Consider: participants, interventions and comparisons, outcomes, resource and policy implications		

For peer review only

Supplementary file 4

Summary of Quality assessment tools used in systematic reviews from literature

	Cochrane collaboration tool	Consensus health economic criteria (CHEC)	NICE	CHEERS statement	Drummond	PHILIPS	Lorga & Moss cost of illness check list	Cost validity scale	National Collaborating Centre for Methods and Tools, 2008. Quality Assessment Tool for Quantitative Studies.
Murthey et al, 2017	A protocol for systematic review of economic evaluation studies conducted on neonatal systematic infections in South East Asia								
Trail based economic evaluation(EE) studies	Y	Y		Y	Y				
Model based EE			Y	Y		Y			
Cost benefit analyses								Y	
Cost of illness studies							Y		
Aminde et al , 2018	Interventions for prevention of cardiovascular disease: a protocol for systematic review of economic evaluations in low and middle income countries								
EE					Y (ranked using NICE scale)	Y			
Model based EE						Y			
Pavey et al, 2012	Effect of exercise referral schemes in primary care on physical activity and improving health outcomes: systematic review and meta-analysis								
EE					Y	Y			
Ruifrok et al, 2014	Study protocol: differential effects of diet and physical activity based interventions in pregnancy on maternal and fetal outcomes—individual patient data (IPD) meta-analysis and health economic evaluation								
EE	Y								
Abu-Omar, 2017	The cost-effectiveness of physical activity interventions: A systematic review of reviews								
Cost effectiveness(CE)									Y
Vijay et al, 2015	Are brief interventions to increase physical activity cost effective? A systematic review								
CE					Y				
Tonmukayakul et al, 2015	Systematic review and quality appraisal of Economic Evaluation Publications in dentistry								
EE					Y				

Conclusions

- Drummond 1996 has been commonly used across the years (2011- 2018) (Pavey et al, 2011, Tonmukayakul et al, 2015, Vijay et al, 2015, Murthey et al, 2017, Aminde et al, 2018)
- Phillips check list has been used across the years to assess the quality of model based economic evaluations(Pavey et al,2011, Murthey et al, 2017, Aminde et al, 2018)
- Lorga & Moss check list for quality assessment in cost of illness studies has been used in review conducted recently in LMIC (Murthey et al, 2017)
- Quality grading has been allocated to Drummond 1996 checklist using NICE scale (Aminde et al, 2018)

The economics of physical activity in low and middle income countries: protocol for a systematic review

Journal:	*BMJ Open*
Manuscript ID	bmjopen-2018-022686.R1
Article Type:	Protocol
Date Submitted by the Author:	05-Jul-2018
Complete List of Authors:	Ranasinghe, Priyanga; Ministry of Health, Nutrition and Indigenous Medicine, ; Brunel Univeristy London, Clinical Sciences<br>Pokhrel, Subhash; Brunel University London, Health Economics Research Group<br>Anokye, Nana; Brunel University, Health Economics Research Group
**Primary Subject Heading**:	Health economics
Secondary Subject Heading:	Public health
Keywords:	HEALTH ECONOMICS, physical activity, low and middle income countries

Title page

The economics of physical activity in low and middle income countries: protocol for a systematic review

Priyanga Diloshini Ranasinghe[2], Subhash Pokhrel[1], Nana Kwame Anokye[1]

[1] Health Economics Theme, Institute of Environment, Health and Societies, Brunel University, London, United Kingdom

[2] Ministry of Health, Nutrition and Indigenous Medicine, Suwasiripaya, Colombo 10, Sri Lanka /Division of Health Sciences, Department of Clinical Sciences, Collage of Health and Life Sciences, Brunel University, London, United Kingdom

Corresponding author: Priyanga Diloshini Ranasinghe

Postal address: Mary Seacole 2nd floor, Division of Health Sciences, Department of Clinical Sciences, Collage of Health and Life Sciences, Brunel University, London, United Kingdom

Email: Priyanga.Ranasinghe@brunel.ac.uk/ priyangaran@yahoo.com

Telephone number: 0044 7424515136/0094 777552794

Subhash Pokhrel Subhash.Pokhrel@brunel.ac.uk

Nana Kwame Anokye Nana.Anokye@brunel.ac.uk

Word count: 3006

For peer review only

Abstract

Introduction: Evidence on the economic costs of physical inactivity, and the cost-effectiveness of physical activity interventions in low- and middle-income countries (LMIC) is sparse, and fragmented where they are available. This is the first review aimed to summarize available evidence on economics of physical activity in LMIC, describe focus and methods underpinning research on economics of physical activity in LMIC, identify potential target variables for policy, identify and report gaps in current knowledge on economics of physical activity in LMIC.

Methods and analysis: Peer reviewed journal articles of observational, experimental, quasi experimental studies and mixed method studies on economics of physical activity in LMIC will be identified by electronic data base search; Scopus, Web of Science and SPORTDiscus. Websites of WHO, NICE international, World Bank and reference lists of included studies will be searched for relevant studies. Study selection process will be a two stage approach; title and abstract screen for inclusion, followed by review of selected full text articles by two independent reviewers. Disagreements will be resolved by consensus and discussion with a third reviewer. Data will be extracted using standardized piloted data extraction forms. Risk of bias will be critically appraised using standard checklists based on study designs. Descriptive synthesis of data is planned. Where relevant, summaries of studies will be classified according to type of economic analysis, country or country category, population, intervention, comparator, outcome and study design. . Meta-analysis will be performed where appropriate. This protocol for systematic review prepared according to Preferred Reporting Items for Systematic review and Meta-analysis for Protocols (PRISMA-P) -2015 statement.

Ethics and dissemination: Ethical approval not obtained as original data will not be collected as part of this review. The completed review will be submitted for publication in a peer-reviewed journal and presentation at conferences.

Systematic review registration: PROSPERO CRD42018099856

For peer review only

Keywords: low and middle income countries; health economics; physical activity

Strengths and limitations of this study:

Strengths

- A review of this kind aimed to summarize available evidence on economics of physical activity, identify gaps in knowledge and provide recommendations for economic research agenda on physical activity has not yet been attempted to best of our knowledge.
- Review with such objectives is timely and fill the current research gap on economics of physical activity in LMIC and future research would be informed by the identified gaps in current knowledge from this review.
- The review would provide a better understanding on research conducted on economics of physical activity in LMIC; countries studied, focus, methods applied, target variables and cost effective interventions (if any) to inform policy on physical activity in LMIC and results of this review would draw more attention and interest of researchers and policy makers, on physical activity as an economic case in LMIC.

Limitations

This review would not include publications that are not peer-reviewed such as unpublished theses and conference presentations and it could affect the sensitivity of this review as the possibility of some important studies being missed cannot be excluded (publication bias cannot be excluded).

- Studies published in any language other than English would not be included for this review and possible language bias cannot be prevented.

For peer review only

INTRODUCTION

Rationale

Physical inactivity is a global health challenge, the fourth leading cause of mortality worldwide [1] and the key risk factor for non-communicable diseases (NCD) such as cardiovascular diseases, cancer and diabetes [2]. Almost one third of global health burden comprise of NCD which account for 1.4 billion disability adjusted life years (DALYs) [3]. At present most of the global NCD burden is from LMIC (as defined by World Bank, Classification 2017) [4] and this burden is largely attributable to population levels of physical inactivity [5,6]. Prevalence of physical inactivity in 2010 in upper middle income countries was 25.4% (19.1%-33.7%), followed by 16.8% (11.8%-26.4%) in lower middle income countries and 16.6% (11.8%-26.4%) in low income countries showing that large population in LMIC[7] are affected by physical inactivity and are therefore at risk of developing NCDs. The data available for the year 2004 shows that 3.2 million deaths worldwide were due to physical inactivity of which 2.6 million were reported from LMIC [8]. LMIC has experienced urbanization, shift of occupations from agriculture to industrial practice and modernization including automation, and resulted in increased level of physical inactivity in these populations [9].

Increasing prevalence of physical activity and reducing the burden of NCD is a main goal of public health policies in LMIC [10]. It is a personal, cooperate and government responsibility to reduce the economic burden of unhealthy lifestyles in LMIC [10]. Economic analysis could uncover subtle characteristics of individual decision making on lifestyle choices; estimate the opportunity costs of not doing anything to improve population levels of physical activity; and help evaluate what interventions work efficiently to achieve the goal of addressing the physical inactivity pandemic, particularly in LMIC [11]. Current focus to promote physical activity has shifted from traditional educational approach to environment and policy approaches. Economic perspectives and public health perspectives complement each other in physical activity promotion [12]. Economic perspectives inform the design of effective physical activity interventions (identifying economic

For peer review only

barriers to physical activity) and the efficient allocation of resources – critical to the economies of LMIC [12].

Our scoping exercise did not identify any published reviews that address the economics of physical activity in LMIC. Thus the economic evidence on physical activity in LMIC could be limited or scattered [13]. Therefore, we designed this review to address following research questions; what is the available evidence base and research gaps on economics of physical activity in LMIC? , what is the focus and methods underpinning economic research on physical activity in LMIC? what are the target variables and cost effective interventions in physical activity for policy in LMIC?

This review will add to scientific literature, an overview of economic evidence base of physical activity in LMIC and gaps in available evidence on this regard. Providing an up to date synthesis of the economic evidence base is an efficient way of highlighting current research practices and new findings to inform researchers, formulation of cost effective physical activity programmes and policies.

Objectives

The review will:

- Summarize available evidence on economics of physical activity in LMIC
- Describe the focus and methods underpinning research on economics of physical activity in LMIC
- Identify potential target variables and cost effective interventions for physical activity in LMIC for policy formation
- Identify and report gaps in research on economics of physical activity in LMIC to provide recommendations for economic research agenda in LMIC

For peer review only

METHODS

The methods for this review were informed by previous reviews of economic analyses of physical activity [10, 13-19] and are in line with recommendations on review of economic evidence[20-22]. This will be based on "Preferred Reporting Items for Systematic Review and Meta Analyses Protocols (PRISMA-P)" Statement [23] (supplementary file 1).

Eligibility criteria

Economic studies on physical activity in LMIC will be included. Specifically, the following study types will be included: (a) economic evaluations of physical activity interventions, (b) economic burden of physical inactivity; (c) cost of physical activity participation, (d) demand for physical activity; (e) and economic correlates of physical (in) activity Physical activity is defined as any bodily movement produced by skeletal muscles that require energy expenditure-including activities undertaken while walking , playing, carrying out household chores, travelling and engaging recreational pursuits[24].

Physical activity interventions will not be specifically defined for this review in order to identify any physical activity intervention in LMIC. However, economic analysis should be carried out along with the intervention, to be included for this review. This strategy will enable to consolidate all available evidence on economics of physical activity related to physical activity interventions.

Eligibility criteria are determined by relevant elements of PICOS criteria [25]. Interventions and comparator will be applicable only to intervention studies.

Inclusion criteria

1. Study setting: Any setting of LMIC in accordance with the definition of LMIC by World Bank, Classification 2017[4].

For peer review only

2. Population: Any population in any age group across the life course
3. Intervention: any physical activity intervention in which the economic evaluation of the intervention has been carried out.
4. Comparator: normal routine, no intervention
5. Outcomes: (a) cost effectiveness ratio(CE ratio), QUALY, incremental cost effectiveness ratio (ICER) assessed as the outcomes of physical activity interventions, (b) cost of physical (in) activity in terms of health care cost and/or productivity loss and/or total cost of physical inactivity (c) measures of association of any economic variable with physical activity are defined as the primary outcomes of this review
6. Study design: Observational studies (cohort, case control, cross sectional); experimental studies including randomised controlled trials (RCTs); quasi experimental studies; natural experiments; and economic evaluation studies7. Studies reported only in English language

Exclusion criteria

1. Case reports, case series, letters to the editor, editorials, reviews, qualitative studies unpublished theses, conference abstracts, and any unobtainable texts.
2. Studies published in a language other than English

Search strategy

We will search the following electronic data bases to identify studies: Scopus (covers 100% MEDLINE coverage, 100% of EMBASE coverage and 100% of Compendex coverage [26]), Web of Science; and SPORTDiscus. Websites of WHO, NICE international, World Bank will be searched for relevant studies. The reference lists of included studies will be searched for any relevant articles. Searches will include publications up to December 2017.

For peer review only

The search strategy was informed by a scoping review that covered relevant reviews on economic studies on physical activity [10,13-19],reviews on physical activity[27-29] and reviews on economic evaluations[30-32-]. The draft search strategy was then reviewed by subject experts and a subject liaison librarian to optimize the sensitivity and the specificity of the search. Supplementary file 2 shows a sample search strategy.

Study Selection

A two stage approach involving three independent reviewers will be used to select relevant papers.

At the first stage, two reviewers (PR and NA) will independently screen the titles and abstracts of identified papers. Disagreement will be resolved through discussions with a third reviewer (SP). We will include a study if in doubt about its inclusion. For example, if the country setting of a study is unclear in the title and abstract, we will take it forward to the next stage.

At the second stage, the full text of papers selected from stage 1 will be reviewed independently by two reviewers (PR and NA). Selected and excluded papers with reasons will be discussed by the two reviewers and a third reviewer (SP) at a consensus meeting and disagreement will be resolved by real time consensus. Corresponding authors will be contacted via email for clarification if the need be. Study selection process will be presented in a PRISMA flow chart [33] along with the reasons for exclusion of studies.

Data management and extraction

Endnote X7 software will be used to manage the search results. Data extraction will be performed using standardized pilot-tested data extraction form developed based on relevant data extraction forms from relevant reviews [17,34,35]. The adaptation process has involved matching the review

For peer review only

objectives with the available data extraction forms. Data from the final selected full text articles will be extracted by one reviewer (PR). To ensure quality of data extraction, a second reviewer (NA) will independently extract data from a random selection of the 50% of final articles Disagreements between two reviewers will be discussed and resolved through discussions with the third reviewer (SP). Any unresolved disagreements will be reported in the final report. Supplementary file 3 shows the draft data extraction form. Data will be extracted for the following items: general information, characteristic of the study, characteristics of population/condition/intervention, data sources/data analysis/outcomes, conclusions and way forward suggested by author, challenges, and quality assessment.

Risk of bias and quality assessment

Risk of bias will be assessed by a one reviewer (PR) for all selected articles using standard checklists selected based on the study design. A second reviewer (NA) will also independently assess the risk of bias on randomly selected 50% of included articles.

Checklists are considered as a reliable means of ensuring that all studies included are critically appraised in a standard way [25]. These will assess the information bias which could occur due to methods; study design, data collection data analysis and interpretation and selection bias where relevant. Most appropriate checklist for each expected study design for risk of bias assessment were selected based on relevant literature in this area [15,17,35-39] and by consensus with all three reviewers (supplementary file 4).Risk of bias will be assessed using following tools;

- Economic evaluation studies

Drummond checklist [40] which consists of 35 items will be used. It mainly assess the quality of the study in relation to methods; study design, data collection, data analysis and interpretations.

For peer review only

If an economic evaluation study is based on a decision analytical model, the Phillips [41] check list will be used in addition

- Cost of illness studies,

The Larg & Moss checklist [42] will be used to assess the quality of the study. It would assess the possible biases related to analytical framework: what costs should have been measured, methodology and data: how well were resource use and productivity losses were measured including representativeness of data for study population, analysis and reporting. Both internal validity and external validity will be appraised.

- Studies on correlation or association

Check list recommended by "The National Institute for Health and Care Excellence" (NICE) [43] will be used Risk of bias related to sample selection, method of selection of exposure/comparison group, outcomes and analysis will be assessed. Both internal and external validity will be appraised.

Quality grading will be given to each included study based on overall quality of the study. Grading for internal validity and external validity will be reported separately, where relevant. The grading process will follow the NICE recommendations [36] (Figure 1):

- Good quality (++): almost all check list criteria have been fulfilled, where they have not been fulfilled the conclusions are very unlikely to alter
- Moderate quality (+) : some of the check list criteria are fulfilled, where they have not been fulfilled or not adequately described, conclusions are unlikely to alter
- Low quality (-): few or no checklist criteria are fulfilled, and conclusions are likely or very likely to alter
- The quality of the included studies will be reported and critically appraised for each study. However, studies will not be excluded based on quality, as this review is particularly aimed

For peer review only

to capture the evidence base on economics of physical activity in LMIC and appraise the methods underpinning the evidence base.

Data synthesis and reporting

A descriptive synthesis of data is planned due to expected heterogeneity of the studies. Summaries of studies will be classified according to type of economic analysis, country or country category, characteristics of the study by PICOS criteria (where applicable); population, intervention, comparator, outcome, study design and quality of studies to describe the overview of available economic evidence base of physical activity in LMIC. Where the numbers permit, summaries of the studies will be categorized based on the focus of the study; economic burden of physical inactivity, economic analysis of physical activity interventions, economic correlates of physical activity including association of physical activity and cost (health care cost and/or productivity loss). Where appropriate, we will conduct a meta-analysis to quantify the effect sizes. Such analyses will adjust for between-study heterogeneity using random effects models. The I-squared (I2) statistic will be used to evaluate the heterogeneity related to the findings of papers. Quantitative analysis will be conducted using STATA 13 software.

We will report the number of studies where data extraction items were not applicable, as an indicator of the quality of reporting. Author stated limitations of included studies and recommendations for future research and policy will be presented. This review will be reported in accordance with PRISMA 2009 statement [33]. Study selection process will be illustrated by flow diagram including the reasons for exclusion of studies at each stage. Search strategy, data extraction form and quality assessment tools will be published as supplementary files. This protocol will not be amended and any changes will be described and discussed in final report.

For peer review only

Study status

Scoping review on economics of physical activity in LMIC was carried out in November 2017. Review on economics of physical activity in LMIC was designed and methods developed in December 2017. Search strategy was piloted in January 2018. Protocol developed in February 2018. First submission of protocol for peer review was on 1st March 2018.

Patient and Public Involvement

Patents and public are not directly involved in this study as original data will not be collected

ETHICS AND DISSEMINATION

Ethical clearance is not required as original data will not be collected. Best practice guidelines to conduct systematic reviews [31] will be followed. Results will be disseminated by peer reviewed publications and conference presentations.

Discussion

Population level of physical inactivity in LMIC is likely to increase giving rise to multitude of public health and economic consequences, including rising healthcare costs due to increased NCD related treatments, increased disability-adjusted life years (DALYs) and productivity losses. Economic research on physical activity from the perspective of LMIC should therefore be considered as a priority area. This is because increased physical inactivity lead to uncontrolled healthcare needs in LMICs, where resources (including that for healthcare) are limited and often shrinking in real terms. Pooling of the available research evidence on economics of physical activity in LMIC will thus reveal the current knowledge on economics of physical

For peer review only

activity in LMIC, thereby guiding the future of national, regional and local policies around physical activity.

This will be the first study to review the evidence base on economics of physical activity in the context of low and middle income countries which can be considered as the main strength of this study. This will identify gaps in knowledge to support future studies in this area. However, we will include the studies published as peer reviewed articles and published in English language only. Thus, possibility of publication bias and language bias cannot be excluded. Furthermore, by assessing and reporting the quality of included studies using standard quality assessment check lists, minimize the possibility of reporting bias of this review.

WHO is in the process of drafting “Global action plan on physical activity (GAPPA) to be implemented from 2018-2030” [44]. This is a timely study as the evidence from this research will provide useful information for implementation and prioritize the actions of GAPPA in LMIC. The evidence from this research will be useful to policy makers and stakeholders dealing with physical activity promotion at individual, local, national, regional and global level with special reference to LMIC. Further, this review will be an important base for a research agenda on economics of physical activity in LMIC. This will be a useful guide for researchers to design research on economics and physical activity with sound methodology, based on the research need of LMIC. Furthermore, information gathered from this research will guide funding agencies for effective allocation of resources.

Authors Contributors: SP and NA developed the idea for the review with inputs from PR. PR wrote the first draft. SP and NA revised the protocol. NA will act as guarantor of the review.

For peer review only

Funding: This research received no specific grant from any funding agency in the public, commercial or not-for-profit sectors. PR is a post-doctoral fellow supported by Ministry of Health, Sri Lanka. NA and SP are employees of Brunel University London.

Conflicts of interest: None

Data sharing statement: All data for this manuscript included with the submission

Supplementary files

1. Comparing the study protocol with PRISMA-P 2015 check list and location in the manuscript
2. Proposed search strategy for the review (sample)
3. Data extraction form illustrating the items for which the data will be extracted and check lists for risk of bias assessment
4. Summary of tools used for risk of bias assessment of relevant literature and basis for the plan of risk of bias assessment in this review

For peer review only

References

1. World Health Organization. (2010). *Global Recommendations on Physical Activity for Health.* Geneva: Retrieved from: <https://www.ncbi.nlm.nih.gov/ books/NBK 305057/> [Accessed on 18 December 2017]

2. World Health Organization. (2017). *Physical activity* [Fact Sheet – Media centre]. Retrieved from: <http://www.who.int/mediacentre/factsheets/fs385/en/ > [Accessed on 6 February 2018]

3. Murray, C. J., Barber, R. M., Foreman, K. J., Abbasoglu Ozgoren, A., Abd-Allah, F., Abera, S. F., Vos, T. (2015). Global, regional, and national disability-adjusted life years (DALYs) for 306 diseases and injuries and healthy life expectancy (HALE) for 188 countries, 1990-2013: quantifying the epidemiological transition. [Research Support, Non-U S Gov't]. *Lancet, 386*(10009), 2145-2191.

4. World Bank. (June 2017).*World Bank Country and Lending Groups: Country classification*. Retrieved from <https://datahelpdesk.worldbank.org/knowledgebase/articles/906519-world-bank-country-and-lending-groups> [Accessed on 01 December 2017]

5. Abegunde, D. O., Mathers, C. D., Adam, T., Ortegon, M., & Strong, K. (2007). The burden and costs of chronic diseases in low-income and middle-income countries. [Review]. *Lancet, 370*(9603), 1929-1938.

6. Hallal, P. C., Andersen, L. B., Bull, F. C., Guthold, R., Haskell, W., & Ekelund, U. (2012). Global physical activity levels: surveillance progress, pitfalls, and prospects. *The Lancet, 380*(9838), 247-257. doi: 10.1016/s0140-6736(12)60646-1

7. WHO. (2015). Prevalence of insufficient physical activity among adults, Data by World Bank income groups. Retrieved from <http://apps.who.int/gho/data/view.main.2487?lang=en> [Accessed on 29 January 2018]

For peer review only

8. WHO. (2009).*Global health risks: mortality and burden of disease attributable to selected major risks*. ISBN 978 92 4 156387 1. Retrieved from <http://www.who.int/healthinfo/global_burden_disease/GlobalHealthRisks_report_full.pdf> [Accessed on 25 January 2018]

9. Atkinson, K., Lowe, S., & Moore, S. (2016). Human development, occupational structure and physical inactivity among 47 low and middle income countries. *Preventive Medicine Reports, 3*, 40-45. doi: https://doi.org/10.1016/j.pmedr.2015.11.009

10. Oldridge, N. B. (2008). Economic burden of physical inactivity: healthcare costs associated with cardiovascular disease. *European Journal of Cardiovascular Prevention & Rehabilitation, 15*(2), 130-139. doi: 10.1097/HJR.0b013e3282f19d42

11. Islam, S. M. S., Purnat, T. D., Phuong, N. T. A., Mwingira, U., Schacht, K., & Fröschl, G. (2014). Non-Communicable Diseases (NCDs) in developing countries: a symposium report. *Globalization and Health*, *10*, 81. http://doi.org/10.1186/s12992-014-0081-9

12. Sturm, R. (2005). Economics and physical activity - A research agenda. *American Journal of Preventive Medicine, 28*(2), 141-149. doi: 10.1016/j.amepre.2004.10.021

13. Ding, D., Kolbe-Alexander, T., Nguyen, B., Katzmarzyk, P. T., Pratt, M., & Lawson, K. D. (2017). The economic burden of physical inactivity: A systematic review and critical appraisal. [Review]. *British Journal of Sports Medicine*, 51(19), 1392-1409. doi: 10.1136/bjsports-2016-097385

For peer review only

14. Jeroen C. M. Barte & G. C. Wanda Wendel-Vos (2017) A Systematic Review of Financial Incentives for Physical Activity: The Effects on Physical Activity and Related Outcomes, *Behavioral Medicine*, 43:2, 79-90, DOI: 10.1080/08964289.2015.1074880

15. Abu-Omar, K., Rütten, A., Burlacu, I., Schätzlein, V., Messing, S., & Suhrcke, M. (2017). The cost-effectiveness of physical activity interventions: A systematic review of reviews. *Preventive Medicine Reports*, *8*, 72–78. http://doi.org/10.1016/j.pmedr.2017.08.006

16. Brown, V., Zapata Diomedi, B., Moodie, M., Veerman, J., & Carter, R. (2016). A systematic review of economic analyses of active transport interventions that include physical activity benefits.*BMJ Open*. (Vol. 45). Retrieved from < http://bmjopen.bmj.com/content/6/9/e011617>

17. Pavey, T. G., Taylor, A. H., Fox, K. R., Hillsdon, M., Anokye, N., Campbell, J. L., Taylor, R. S. (2011). Effect of exercise referral schemes in primary care on physical activity and improving health outcomes: systematic review and meta-analysis. *BMJ, 343*. doi: 10.1136/bmj.d6462

18. Campbell, F., Holmes, M., Everson-Hock, E., Davis, S., Woods, H. B., Anokye, N., Kaltenthaler, E. (2015). A systematic review and economic evaluation of exercise referral schemes in primary care: a short report. *Health Technology Assessment, 19*(60), 1-+. doi: 10.3310/hta19600

19. Suhrcke, M., Boluarte, T. A., & Niessen, L. (2012). A systematic review of economic evaluations of interventions to tackle cardiovascular disease in low- and middle-income countries. [journal article]. *BMC Public Health, 12*(1), 2. doi: 10.1186/1471-2458-12-2

For peer review only

20. Kaltenthaler, E., Tappenden, P., & Paisley, S. (2013). Reviewing the evidence to inform the population of cost-effectiveness models within health technology assessments. [Research Support, Non-U S Gov't]. *Value Health, 16*(5), 830-836. Retrieved from < https://www.ncbi.nlm.nih.gov/pubmed/23947977>

21. Zechmeister-Koss, I., Schnell-Inderst, P., & Zauner, G. (2014). Appropriate evidence sources for populating decision analytic models within health technology assessment (HTA): a systematic review of HTA manuals and health economic guidelines. [Review]. *Med Decis Making, 34*(3), 288-299. http://journals.sagepub.com/doi/abs/10.1177/0272989X13509406

22. NICE (2012). *The guideline manual Process and methods* (PMG6) Published date: November 2012. Retrieved from < https://www.nice.org.uk/process/pmg6/resources/the-guidelines-manual-appendices-bi-2549703709/chapter/appendix-b-methodology-checklist-systematic-reviews-and-meta-analyses>

23. Moher, D., Shamseer, L., Clarke, M., Ghersi, D., Liberati, A., Petticrew, M., Stewart, L. A. (2015). Preferred reporting items for systematic review and meta-analysis protocols (PRISMA-P) 2015 statement. [Research Support, Non-U S Gov't Research Support, U S Gov't, P H S]. *Syst Rev, 4*(1), 2046-4053.

24. WHO. (2018). *Physical Activity*.[Fact sheet]. Retrieved from <http://www.who.int/en/news-room/fact-sheets/detail/physical-activity > [Accessed on 15 January 2018]

25. University of, Y. (2009). *Systematic reviews: CRD's guidance for undertaking reviews in health care*. York: CRD, University of York. Retrieved from <https://www.york.ac.uk/media/crd/Systematic_Reviews.pdf> [Accessed on 29 December 2017]

26. Burnham, J. F. (2006). Scopus database: a review. Biomed *Digit Libr*, 3(1), 1742-5581. https://doi.org/10.1186/1742-5581-3-1

27. Milton, K., Macniven, R., & Bauman, A. (2014). Review of the epidemiological evidence for physical activity and health from low- and middle-income countries. [Review]. *Glob Public Health, 9*(4), 369-381.

28. Lachat, C., Otchere, S., Roberfroid, D., Abdulai, A., Seret, F. M., Milesevic, J., Kolsteren, P. (2013). Diet and physical activity for the prevention of non-communicable diseases in low- and middle-income countries: a systematic policy review. [Meta-Analysis Review]. *PLoS Med, 10*(6), 11.

29. Hunter, R. F., Christian, H., Veitch, J., Astell-Burt, T., Hipp, J. A., & Schipperijn, J. (2015). The impact of interventions to promote physical activity in urban green space: a systematic review and recommendations for future research. [Research Support, Non-U S Gov't Review]. *Soc Sci Med, 124*, 246-256.

30. Sutton, L., Karan, A., & Mahal, A. (2014). Evidence for cost-effectiveness of lifestyle primary preventions for cardiovascular disease in the Asia-Pacific Region: a systematic review. *Globalization and Health*, *10*, 79. http://doi.org/10.1186/s12992-014-0079-3

31. Santatiwongchai, B., Chantarastapornchit, V., Wilkinson, T., Thiboonboon, K., Rattanavipapong, W., Walker, D. G., Teerawattananon, Y. (2015). Methodological Variation in Economic Evaluations Conducted in Low- and Middle-Income Countries: Information for Reference Case Development. *PLoS ONE, 10*(5), e0123853. doi: 10.1371/journal.pone.0123853

For peer review only

32. Nosratnejad, S., Rashidian, A., & Dror, D. M. (2016). Systematic Review of Willingness to Pay for Health Insurance in Low and Middle Income Countries. [Review]. *PLoS One, 11*(6).

33. Moher, D., Liberati, A., Tetzlaff, J., & Altman, D. G. (2009). Preferred reporting items for systematic reviews and meta-analyses: the PRISMA statement. [Guideline Research Support, Non-U S Gov't]. *J Clin Epidemiol, 62*(10), 1006-1012.

34. Pokhrel, S., Quigley, M. A., Fox-Rushby, J., McCormick, F., Williams, A., Trueman, P., Renfrew, M. J. (2015). Potential economic impacts from improving breastfeeding rates in the UK. [Research Support, Non-U.S. Gov't]. *Arch Dis Child, 100*(4), 334-340. doi: 10.1136/archdischild-2014-306701

35. Murthy, S., John, D., Godinho, I. P., Godinho, M. A., Guddattu, V., & Nair, N. S. (2017). A protocol for a systematic review of economic evaluation studies conducted on neonatal systemic infections in South Asia. *Syst Rev, 6*(1), 252. doi: 10.1186/s13643-017-0648-7

36. Aminde, L. N., & Veerman, L. (2016). Interventions for the prevention of cardiovascular diseases: a protocol for a systematic review of economic evaluations in low-income and middle-income countries. *BMJ Open, 6*(12), e013668. doi: 10.1136/bmjopen-2016-013668

37. Ruifrok, A. E., Rogozinska, E., van Poppel, M. N., Rayanagoudar, G., Kerry, S., de Groot, C. J., Thangaratinam, S. (2014). Study protocol: differential effects of diet and physical activity based interventions in pregnancy on maternal and fetal outcomes—individual patient data

For peer review only

(IPD) meta-analysis and health economic evaluation. [Journal article]. *Systematic Reviews, 3*(1), 131. doi: 10.1186/2046-4053-3-131

38. GC, V., Wilson, E. C., Suhrcke, M., Hardeman, W., & Sutton, S. (2015). Are brief interventions to increase physical activity cost-effective? A systematic review. *British Journal of Sports Medicine*. doi: 10.1136/bjsports-2015-094655

39. Tonmukayakul, U., Calache, H.,Clark, R., Wasiak, J., Faggion Jr, C.M. (2015). Systematic review and quality appraisal of economic evaluation publications in Dentistry. *Journal of Dental research*,94(10), 1348-1354. https://doi.org/10.1177/0022034515589958

40. Drummond, M. F., & Jefferson, T. O. (1996). Guidelines for authors and peer reviewers of economic submissions to the BMJ. *BMJ, 313*(7052), 275-283. doi: 10.1136/bmj.313.7052.275

41. Philips, Z., Ginnelly, L., Sculpher, M., Claxton, K., Golder, S., Riemsma, R., Glanville, J. (2004). Review of guidelines for good practice in decision-analytic modelling in health technology assessment. [Review]. *Health Technol Assess, 8*(36), 1-158.

42. Larg, A., & Moss, J. R. (2011). Cost-of-illness studies: a guide to critical evaluation. [Research Support, Non-U S Gov't]. *Pharmacoeconomics, 29*(8), 653-671.

43. National Institute for Health and Excellence (NICE). (2012). *Methods for the development of NICE public health guidance* (third edition), Appendix G Quality appraisal checklist- quantitative studies reporting correlations and associations. Retrieved from< https://www.nice.org.uk/process/pmg4/chapter/appendix-g-quality-appraisal-checklist-quantitative-studies-reporting-correlations-and >[Accessed on 02 January 2018)

For peer review only

44. World Health Organization. (2018). *Non communicable diseases and their risk factors, Governance: Development of draft global action plan to promote physical activity*. Retrieved from < http://www.who.int/ncds/governance/physical_activity_plan/en/> [Accessed on 10 January 2018]

For peer review only

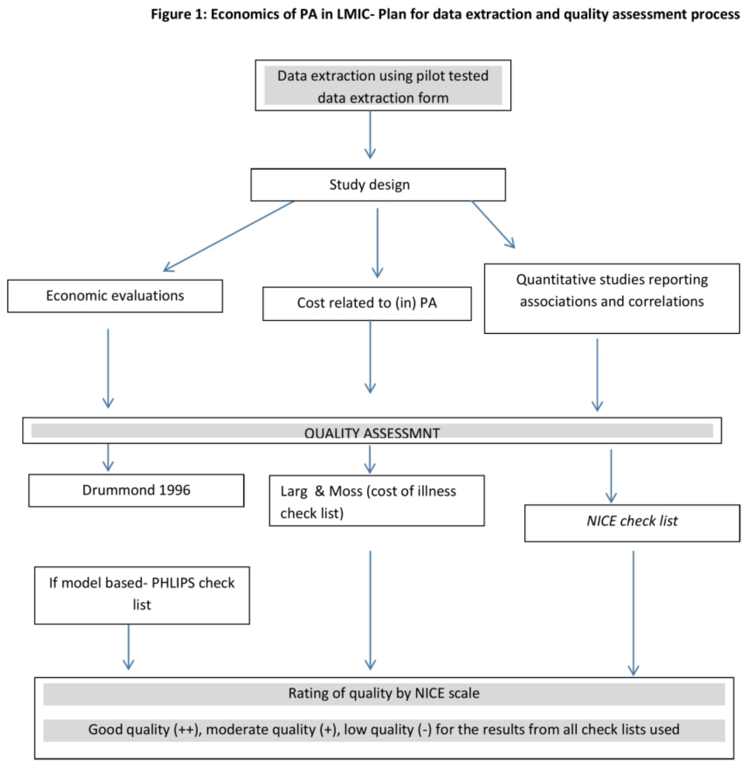

Figure 1: Economics of PA in LMIC- Plan for data extraction and quality assessment process

210x297mm (300 x 300 DPI)

PRISMA-P 2015 Checklist

This checklist has been adapted for use with systematic review protocol submissions to BioMed Central journals from Table 3 in Moher D et al: Preferred reporting items for systematic review and meta-analysis protocols (PRISMA-P) 2015 statement. *Systematic Reviews* 2015 **4**:1

Section/topic	#	Checklist item	Information reported: Yes	Information reported: No	Line number(s)
**ADMINISTRATIVE INFORMATION**					
**Title**					
Identification	1a	Identify the report as a protocol of a systematic review	☒	☐	2
Update	1b	If the protocol is for an update of a previous systematic review, identify as such	☐	☒	
**Registration**	2	If registered, provide the name of the registry (e.g., PROSPERO) and registration number in the Abstract	☒	☐	47
**Aut4h2ors**					
Contact	3a	Provide name, institutional affiliation, and e-mail address of all protocol authors; provide physical mailing address of corresponding author	☒	☐	4-18
Contributions	3b	Describe contributions of protocol authors and identify the guarantor of the review	☒	☐	283-284
**Amendments**	4	If the protocol represents an amendment of a previously completed or published protocol, identify as such and list changes; otherwise, state plan for documenting important protocol amendments	☒	☐	236-237
**Support**					
Sources	5a	Indicate sources of financial or other support for the review	☒	☐	285-287
Sponsor	5b	Provide name for the review funder and/or sponsor	☐	☒	
Role of sponsor/funder	5c	Describe roles of funder(s), sponsor(s), and/or institution(s), if any, in developing the protocol	☐	☒	
**INTRODUCTION**					
**Rationale**	6	Describe the rationale for the review in the context of what is already known	☒	☐	50-86
**Objectives**	7	Provide an explicit statement of the question(s) the review will address with reference to	☒	☐	78-81,88-96

BioMed Central The Open Access Publisher

Section/topic	#	Checklist item	Information reported		Line number(s)
			Yes	No	
		participants, interventions, comparators, and outcomes (PICO)			
**METHODS**					
**Eligibility criteria**	8	Specify the study characteristics (e.g., PICO, study design, setting, time frame) and report characteristics (e.g., years considered, language, publication status) to be used as criteria for eligibility for the review	☒	☐	105-136,142-143
**Information sources**	9	Describe all intended information sources (e.g., electronic databases, contact with study authors, trial registers, or other grey literature sources) with planned dates of coverage	☒	☐	138-148
**Search strategy**	10	Present draft of search strategy to be used for at least one electronic database, including planned limits, such that it could be repeated	☐	☒	147-148 Supplementary file 2
STUDY RECORDS					
Data management	11a	Describe the mechanism(s) that will be used to manage records and data throughout the review	☒	☐	164-176
Selection process	11b	State the process that will be used for selecting studies (e.g., two independent reviewers) through each phase of the review (i.e., screening, eligibility, and inclusion in meta-analysis)1	☒	☐	150-162
Data collection process	11c	Describe planned method of extracting data from reports (e.g., piloting forms, done independently, in duplicate), any processes for obtaining and confirming data from investigators	☒	☐	165-176
**Data items**	12	List and define all variables for which data will be sought (e.g., PICO items, funding sources), any pre-planned data assumptions and simplifications	☒	☐	172-176 Supplementary file 3
**Outcomes and prioritization**	13	List and define all outcomes for which data will be sought, including prioritization of main and additional outcomes, with rationale	☒	☐	125-129 Supplementary file 3
**Risk of bias in individual studies**	14	Describe anticipated methods for assessing risk of bias of individual studies, including whether this will be done at the outcome or study level, or both; state how this information will be used in data synthesis	☒	☐	151-169 Supplementary file 3
DATA					
**Synthesis**	15a	Describe criteria under which study data will be quantitatively synthesized	☐	☒	
	15b	If data are appropriate for quantitative synthesis, describe planned summary measures, methods of handling data, and methods of combining data from studies, including any planned exploration of	☒	☐	178-216

BioMed Central The Open Access Publisher

For peer review only

3

Section/topic	#	Checklist item	Information reported Yes	Information reported No	Line number(s)
		consistency (e.g., I^2, Kendall's tau)			
	15c	Describe any proposed additional analyses (e.g., sensitivity or subgroup analyses, meta-regression)	☒	☐	226-230
	15d	If quantitative synthesis is not appropriate, describe the type of summary planned	☒	☐	219-226
**Meta-bias(es)**	16	Specify any planned assessment of meta-bias(es) (e.g., publication bias across studies, selective reporting within studies)	☐	☒	
**Confidence in cumulative evidence**	17	Describe how the strength of the body of evidence will be assessed (e.g., GRADE)	☒	☐	205-216

For peer review only

BioMed Central The Open Access Publisher

Supplementary file 2: Proposed search strategy for Scopus

	Proposed preliminary search strategy for Scopus
#1	Domain 1: Physical activity<br>*"physical activity"* OR *"physical exercise"* OR *"physical inactivity"* OR *"physical fitness"* OR *"Sedentary"* OR *"active lifestyle"* OR "active *living"* OR "active *leisure"* OR "active *play"* OR "active *recreation"* OR "active *commuting"*
#2	Domain 2: Economics<br>*"economics"* OR *"economic evaluation"* OR *"economic burden"* OR *"health care cost"* OR *"cost analyses"* OR "cost *effectiveness"* OR "cost *benefit"* OR "cost *minimization"* OR *"cost utility"* OR "cost *saving"* OR "cost *outcome"* OR "cost *consequence"*<br>OR *"demand"* OR *"supply"* OR *"market"* OR *"price"* OR *"incentives"* OR *"willingness to pay"* OR *"time use"*
#3	#1 AND #2

For peer review only

Supplementary file 3

DRAFT DATA EXTRACTION FORM FOR REVIEW ON ECONONOMICS AND PYSICAL ACTIVITY IN LOW AND MIDDLE INCOME COUNTRIES (ADAPTED FROM MURTHEY ET AL., 2017, POKHREL ET AL., 2014, PAVEY ET AL., 2011)			
No.	Item category	Items	Notes
1	General information	1.1 Study ID<br>1.2 Review author ID<br>1.3 Date of review<br>1.4 Title, authors(s)<br>1.5 Year of publication<br>1.6 Country studies<br>1.7 Income level of the country	
2	Characteristics of the study	2.1 Aims and objectives/research question<br>2.2. Eligibility decision/Inclusion and exclusion criteria (?)<br>2.3 Study design<br>2.4 Study setting<br>2.5 Total study duration<br>2.6 Sample size<br>2.7 Sample size calculation<br>2.8 Recruitment<br>2.9 Sample selection<br>2.10 Method of allocation<br>2.11 Perspective (i.e. Societal, health care system, service provider etc.)	
3	Characteristics of the population/condition /intervention	**Population/participants**<br>3.1 Type of population<br>(i.e. General population university students, group of employees)<br>3.2 Age<br>3.3 Gender<br>3.4 Ethnicity<br>**Condition**<br>3.5 Definition of physical activity /physical inactivity/sedentary	

		3.6Definition of disease condition<br>**Intervention**<br>3.7 Type of physical activity/programme<br>3.9 Comparator	
4	Data sources/Data analysis/results	**Scope**<br>4.1 Type of analysis<br>(Ie. Costing/economic evaluation/association of economic factors with physical activity/other)<br>4.2 Form of economic evaluation<br>4.3 Time horizon<br>4.4 Study assumptions<br>**Cost**<br>4.5 Reported costs/cost components<br>4.6 Data sources for cost measures<br>4.7 How cost was reported<br>(i.e. marginal cost/ average cost/incremental cost/total cost/other)<br>4.8 Discount rate<br>4.9 Year of costing<br>4.10 Currency unit<br>4.11 Currency conversion rate<br>4.12 Inflation adjustments<br>**Outcomes**<br>4.13 Reported outcomes<br>(i.e. measures of effectiveness/efficacy/measures of effect)<br>4.14 Data sources for the outcome measures<br>4.15 Discount rate<br>**Sensitivity analysis**<br>4.16 Performed/ not<br>4.17 Type of analysis<br>(i.e. deterministic/ stochastic)<br>4.18 Variables used for sensitivity analysis<br>4.19 Results of sensitivity analysis<br>**Main results**	

For peer review only

		4.20 Cost / cost effectiveness<br>4.21 Other	
5	Conclusions and way forward	5.1 Key Conclusion(s)<br>5.2 Suggestions by author for further research	
6	Challenges	6.1 Author stated limitations<br>6.2 Author stated strengths<br>6.3 Miscellaneous comments from author<br>6.4 Funding source<br>6.5 Conflicts of interest	
7.	Quality assessment of studies	7.1 Economic evaluation studies- Drummond checklist<br>7.2 Model based economic evaluations-Phillips check list<br>7.3 Cost of illness/cost of risk factor study/cost estimation- check list by Larg and Moss<br>7.4 Quantitative study reporting correlation or association? NICE check list for quantitative studies reporting correlations and associations	

**Method of rating the quality of studies from each check list (A, B, C, D) in accordance to the quality appraisal by NICE scale (Methods for the development of NICE public health guidance (third edition))**	
• Reviewer is requested to complete in detail the comment section of quality appraisal check lists so that the grade awarded for each study aspect is as transparent as possible<br>• At the end of completion of the check list each study is awarded an overall quality grading for internal validity (IV)and a separate one for external validity (EV)	
All or most of the checklist criteria have been fulfilled, where they have not been fulfilled the conclusions are very unlikely to alter	++
Some of the checklist criteria have been fulfilled, where they have not been fulfilled, or not adequately described, the conclusions are unlikely to alter	+
Few or no checklist criteria have been fulfilled and the conclusions are likely or very likely to alter.	-

For peer review only

CHECKLIST A

QUALITY ASSESSMENT FOR ECONOMIC EVALUATION(CHECKLIST FROM DRUMMOND AND JEFFERSON 1996)	Yes	No	Not clear	Not appropriate
**Study design**				
1) The research question is stated				
2) The economic importance of the research question is stated				
3) The viewpoint(s) of the analysis are clearly stated and justified				
4) The rationale for choosing the alternative programmes or interventions compared is stated				
5) The alternatives being compared are clearly described				
6) The form of economic evaluation used is stated				
7) The choice of form of economic evaluation is justified in relation to the questions addressed				
**Data collection**				
8) The source(s) of effectiveness estimates used are stated				
9) Details of the design and results of effectiveness study are given (if based on a single study)				
10) Details of the method of synthesis or meta-analysis of estimates are given (if based on an overview of a number				

For peer review only

QUALITY ASSESSMENT FOR ECONOMIC EVALUATION(CHECKLIST FROM DRUMMOND AND JEFFERSON 1996)	Yes	No	Not clear	Not appropriate
of effectiveness studies)				
11) The primary outcome measure(s) for the economic evaluation are clearly stated				
12) Methods to value health states and other benefits are stated				
13) Details of the subjects from whom valuations were obtained are given				
14) Productivity changes (if included) are reported separately				
15) The relevance of productivity changes to the study question is discussed				
16) Quantities of resources are reported separately from their unit costs				
17) Methods for the estimation of quantities and unit costs are described				
18) Currency and price data are recorded				
19) Details of currency of price adjustments for inflation or currency conversion are given				
20) Details of any model used are given				
21) The choice of model used and the key parameters on which it is based are justified				
**Analysis and interpretation of results**				
22) Time horizon of costs and benefits is stated				
23) The discount rate(s) is stated				

For peer review only

QUALITY ASSESSMENT FOR ECONOMIC EVALUATION(CHECKLIST FROM DRUMMOND AND JEFFERSON 1996)	Yes	No	Not clear	Not appropriate
24) The choice of rate(s) is justified				
25) An explanation is given if costs or benefits are not discounted				
26) Details of statistical tests and confidence intervals are given for stochastic data				
27) The approach to sensitivity analysis is given				
28) The choice of variables for sensitivity analysis is justified				
29) The ranges over which the variables are varied are stated				
30) Relevant alternatives are compared				
31) Incremental analysis is reported				
32) Major outcomes are presented in a disaggregated as well as aggregated form				
33) The answer to the study question is given				
34) Conclusions follow from the data reported				
35) Conclusions are accompanied by the appropriate caveats				

Did the economic evaluation use a decision-analytic modelling framework?	Response: Yes(✓), No (✗) , Not applicable (NA)<br><br>Instruction: If *Yes*, assess paper using the questions in Check list B, If *No/NA*, skip check list B

For peer review only

CHECKLIST B

QUALITY ASSESSMENT FOR DECISION-ANALYTIC MODELS(CHECKLIST FROM PHILIPS ET AL. 2004)			
**Quality criterion**	**Question(s)**	**Response (✓, ✗, NA)**	**Comments**
S1	Is there a clear statement of the decision problem?		
	Is the objective of the evaluation and model specified and consistent with the stated decision problem?		
	Is the primary decision maker specified?		
S2	Is the perspective of the model stated clearly?		
	Are the model inputs consistent with the stated perspective?		
	Has the scope of the model been stated and justified?		
	Are the outcomes of the model consistent with the perspective, scope and overall objective of the model?		
S3	Is the structure of the model consistent with a coherent theory of the health condition under evaluation?		
	Are the sources of data used to develop the structure of the model specified?		

For peer review only

QUALITY ASSESSMENT FOR DECISION-ANALYTIC MODELS(CHECKLIST FROM PHILIPS ET AL. 2004)			
**Quality criterion**	**Question(s)**	**Response (✓, ×, NA)**	**Comments**
	Are the causal relationships described by the model structure justified appropriately?		
S4	Are the structural assumptions transparent and justified?		
	Are the structural assumptions reasonable given the overall objective, perspective and scope of the model?		
S5	Is there a clear definition of the options under evaluation?		
	Have all feasible and practical options been evaluated?		
	Is there justification for the exclusion for the exclusion of feasible options?		
S6	Is the chosen model type appropriate given the decision problem and specified causal relationships within the model?		
S7	Is the time horizon of the model sufficient to reflect all important differences between options?		
	Are the time horizon of the model, the duration of treatment and the duration of treatment effect described and justified?		
S8	Do the disease states (state transition model) or the pathways (decision tree model) reflect the underlying biological process of the		

For peer review only

QUALITY ASSESSMENT FOR DECISION-ANALYTIC MODELS(CHECKLIST FROM PHILIPS ET AL. 2004)			
**Quality criterion**	**Question(s)**	**Response (✓, ×, NA)**	**Comments**
	disease in question and the impact of interventions?		
S9	Is the cycle length defined and justified in terms of natural history of disease?		
D1	Are the data identification methods transparent and appropriate given the objectives of the model?		
	Where choices have been made between data sources, are these justified appropriately?		
	Has particular attention been paid to identifying data for the important parameters in the model?		
	Has the quality of the data been assessed appropriately?		
	Where expert opinion has been used, are the methods described and justified?		
D2	Is the data modelling methodology based on justifiable statistical and epidemiological techniques?		
D2a	Is the choice of baseline data described and justified?		
	Are transition probabilities calculated appropriately?		

For peer review only

QUALITY ASSESSMENT FOR DECISION-ANALYTIC MODELS(CHECKLIST FROM PHILIPS ET AL. 2004)			
**Quality criterion**	**Question(s)**	**Response (✓, ×, NA)**	**Comments**
	Has a half-cycle correction been applied to both cost and outcome?		
	If not, has this omission been justified?		
D2b	If relative treatment effects have been derived from trail data, have they been synthesised using appropriate techniques?		
	Have the methods and assumptions used to extrapolate short-term results to final outcomes been documented and justified?		
	Have alternative extrapolation assumptions been explored through sensitivity analysis?		
	Have assumptions regarding the continuing effect of treatment once treatment is complete been documented and justified?		
	Have alternative assumptions regarding the continuing effect of treatment been explored through sensitivity analysis?		
D2c	Are the costs incorporated into the model justified?		
	Has the source for all costs been described?		
	Have discount rates been described and justified given the target decision-maker?		

For peer review only

QUALITY ASSESSMENT FOR DECISION-ANALYTIC MODELS(CHECKLIST FROM PHILIPS ET AL. 2004)			
**Quality criterion**	**Question(s)**	**Response (✓, ×, NA)**	**Comments**
D2d	Are the utilities incorporated into the model appropriate?		
	Is the source for the utility weights referenced?		
	Are the methods of derivation for the utility weights justified?		
D3	Have all data incorporated into the model been described and referenced in sufficient detail?		
	Has the use of mutually inconsistent data been justified (i.e. are assumptions and choices appropriate)?		
	Is the process of data incorporation transparent?		
	If data have been incorporated as distributions, has the choice of distribution for each parameter been described and justified?		
	If data have been incorporated as distributions, is it clear that second order uncertainty is reflected?		
D4	Have the four principal types of uncertainty been addressed?		
	If not, has the omission of particular forms of uncertainty been justified?		
D4a	Have methodological uncertainties been addressed by running alternative versions of the model with different methodological		

For peer review only

QUALITY ASSESSMENT FOR DECISION-ANALYTIC MODELS(CHECKLIST FROM PHILIPS ET AL. 2004)			
**Quality criterion**	**Question(s)**	**Response (✓, ×, NA)**	**Comments**
	assumptions?		
D4b	Is there evidence that structural uncertainties have been addressed via sensitivity analysis?		
D4c	Has heterogeneity been dealt with by running the model separately for different subgroups?		
D4d	Are the methods of assessment of parameter uncertainty appropriate?		
	If data are incorporated at point estimates, are the ranges used for sensitivity analysis stated clearly and justified?		
C1	Is there evidence that the mathematical logic of the model has been tested thoroughly before use?		
C2	Are any counterintuitive results from the model explained and justified?		
	If the model has been calibrated against independent data, has any differences been explained and justified?		
	Have the results of the model been compared with those of previous models and any differences in results explained?		

For peer review only

CHECKLIST C

QUALITY ASSESSMNET FOR COST OF ILLNESS STUDIES (ADAPTED FROM LARG AND MOSS, 2011)			
Responses (marked Similar to NICE check list, write the answer where relevant under the comment section)<br><br>++ Indicates that for that particular aspect of study design, the study has been designed or conducted in such a way as to minimise the risk of bias.<br>+ Indicates that either the answer to the checklist question is not clear from the way the study is reported, or that the study may not have addressed all potential sources of bias for that particular aspect of study design.<br>- Should be reserved for those aspects of the study design in which significant sources of bias may persist.<br>NR (Not Reported) - Should be reserved for those aspects in which the study under review fails to report how they have (or might have) been considered.<br>NA (Not Applicable) Should be reserved for those study design aspects that are not applicable given the study design under review (for example, allocation concealment would not be applicable for case–control studies).			
	Question	Response ***++/+/-NR/NA***	Comment
1.	Analytical framework: what costs should have been measured?<br><br>(a) What was the motivation and perspective of the study?<br><br>(b) Was the appropriate epidemiologic approach taken?<br><br>(c) Was the study question well specified?<br>(i) Were all relevant, non-trivial cost components and their stakeholders identified?<br>(ii) Were necessary timeframes specified?<br>(iii) Was a case of disease or risk factor adequately and appropriately defined?<br>(iv) Was the counterfactual population occurrence plausible and meaningful?		
2.	(2) Methodology and data: how well were resource use and productivity losses measured?		

For peer review only

	(a) Was an appropriate method(s) of quantification used, such that;<br>(i) Additional, or excess, costs were measured?<br>(ii) only costs specific to (caused by) the health problem were included (confounders controlled)?<br>(iii) All important effects were captured?<br>(iv) Important differences across subpopulations were accounted for?<br>(v) The required level of detail could be provided?<br><br>(b) Was the resource quantification method(s) well executed?<br>(i) For population-based studies, were cost allocation methods, data and assumptions valid?<br>(ii) For person-based studies, were appropriate statistical tests performed and reported?<br>(iii) Were data representative of the study population?<br>(iv) Were there any other relevant resource quantification issues?<br><br>(c) Were healthcare resources valued appropriately?<br><br>(d) Was the approach for valuing production losses justified, and assumptions valid?<br><br>(e) Was the inclusion of intangible costs appropriate:<br>(i) Was double counting of mortality-related production losses avoided?<br>(ii) Were losses valued appropriately, given the study's perspective?		
3	Analysis and reporting<br>(a) Did the analysis address the study question?<br><br>(b) Was a range of estimates presented?		

For peer review only

	(c) Were the main uncertainties identified?<br><br>(d) Was a sensitivity analysis performed on:<br><br>(i) important (uncertain) parameter estimates?<br>(ii) key assumptions? (including the counterfactual)<br>(iii) point estimates? (based on confidence or credible intervals)<br><br>(e) Was adequate documentation and justification given for cost components, data and sources, assumptions and methods?<br><br>(f) Was uncertainty around the estimates and its implications adequately discussed?<br><br>(g) Were important limitations discussed regarding the cost components, data, assumptions and methods?<br><br>(h) Were the results presented at the appropriate level of detail to answer the study question (cost components; disease subtypes, severity, stage; subpopulation groups, cost bearers)?		

For peer review only

CHECKLIST D

QUALITY ASSESSMENT FOR QUANTITATIVE STUDIES REPORTING CORRELATIONS AND ASSOCIATIONS(NICE,2012 BASED ON 'GRAPHICAL APPRAISAL TOOL FOR EPIDEMIOLOGICAL STUDIES(GATE)` BY JACKSON ET AL. (2006)			
Responses:<br><br>++ Indicates that for that particular aspect of study design, the study has been designed or conducted in such a way as to minimise the risk of bias.<br><br>+ Indicates that either the answer to the checklist question is not clear from the way the study is reported, or that the study may not have addressed all potential sources of bias for that particular aspect of study design.<br><br>- Should be reserved for those aspects of the study design in which significant sources of bias may persist.<br><br>NR (Not Reported) - Should be reserved for those aspects in which the study under review fails to report how they have (or might have) been considered.<br><br>NA (Not Applicable) Should be reserved for those study design aspects that are not applicable given the study design under review (for example, allocation concealment would not be applicable for case–control studies).			
**Section 1: Population**			
**Numb er**	**Question**	**Response**<br><br>**++/+/-/ NR/NA**	**Comments**
1.1	Is the source population or source area well described?<br><br>Was the country (e.g. developed or non-developed, type of health care system), setting (primary schools, community centres etc), location (urban, rural), population demographics etc adequately described?		

1.2	Is the eligible population or area representative of the source population or area?<br><br>Was the recruitment of individuals, clusters or areas well defined (e.g. advertisement, birth register)?<br><br>Was the eligible population representative of the source? Were important groups underrepresented?		
1.3	Do the selected participants or areas represent the eligible population or area?<br><br>Was the method of selection of participants from the eligible population well described?<br><br>What % of selected individuals or clusters agreed to participate? Were there any sources of bias?<br><br>Were the inclusion or exclusion criteria explicit and appropriate?		
**Section 2: Method of selection of exposure (or comparison) group**			
2.1	Selection of exposure (and comparison) group. How was selection bias minimised?<br><br>How was selection bias minimised?		
2.2	Was the selection of explanatory variables based on a sound theoretical basis?<br><br>How sound was the theoretical basis for selecting the explanatory variables?		
2.3	Was the contamination acceptably low?<br><br>Did any in the comparison group receive the exposure?		

For peer review only

	If so, was it sufficient to cause important bias?		
2.4	How well were likely confounding factors identified and controlled?<br><br>Were there likely to be other confounding factors not considered or appropriately adjusted for?<br><br>Was this sufficient to cause important bias?		
**Section 3: Outcomes**			
3.1	Were the outcome measures and procedures reliable?<br><br>Were outcome measures subjective or objective (e.g. biochemically validated nicotine levels ++ vs self-reported smoking −)?<br><br>How reliable were outcome measures (e.g. inter- or intra-rater reliability scores)?<br><br>Was there any indication that measures had been validated (e.g. validated against a gold standard measure or assessed for content validity)?		
3.2	Were the outcome measurements complete?<br><br>Were all or most of the study participants who met the defined study outcome definitions likely to have been identified?		
3.3	Were all important outcomes assessed?<br><br>Were all benefits and harms assessed?<br><br>Was it possible to determine the overall balance of benefits and harms of		

For peer review only

	intervention versus comparisons?		
3.4	Was there a similar follow-up time in exposure and comparison groups?<br><br>If groups are followed for different lengths of time, then more events are likely to occur in the group followed-up for longer distorting the comparison.<br><br>Analyses can be adjusted to allow for differences in length of follow-up (e.g. using person-years).		
3.5	Was follow-up time meaningful?<br><br>Was follow-up long enough to assess long-term benefits and harms?<br><br>Was it too long, e.g. participants lost to follow-up?		
**Section 4: Analyses**			
4.1	Was the study sufficiently powered to detect an intervention effect (if one exists)?<br><br>A power of 0.8 (i.e. it is likely to see an effect of a given size if one exists, 80% of the time) is the conventionally accepted standard.<br><br>Is a power calculation presented? If not, what is the expected effect size? Is the sample size adequate?		
4.2	Were multiple explanatory variables considered in the analyses?<br><br>Were there sufficient explanatory variables considered in the analysis?		

For peer review only

4.3	Were the analytical methods appropriate?<br><br>Were important differences in follow-up time and likely confounders adjusted for?		
4.4	Was the precision of association given or calculable? Is association meaningful?<br><br>Were confidence intervals or p values for effect estimates given or possible to calculate?<br><br>Were CIs wide or were they sufficiently precise to aid decision-making? If precision is lacking, is this because the study is under-powered?		
**Section 5: Summary**			
5.1	Are the study results internally valid (i.e. unbiased)?<br><br>How well did the study minimise sources of bias (i.e. adjusting for potential confounders)?<br><br>Were there significant flaws in the study design?		
5.2	Are the findings generalizable to the source population (i.e. externally valid)?<br><br>Are there sufficient details given about the study to determine if the findings are generalizable to the source population?<br><br>Consider: participants, interventions and comparisons, outcomes, resource and policy implications		

For peer review only

Supplementary file 4

Summary of Quality assessment tools used in systematic reviews from literature

	Quality assessment tools	Cochrane collaboration tool	Consensus health economic criteria (CHEC)	NICE	CHEERS statement	Drummond	PHILIPS	Larg & Moss cost of illness check list	Cost validity scale	National Collaborating Centre for Methods and Tools, 2008. Quality Assessment Tool for Quantitative Studies.
Systematic reviews and the type of economic analysis										
1.Murthey et al, 2017[1]										
• Trial l based economic evaluation(EE) studies		+	+		+	+				
• Model based EE				+	+		+			
• Cost benefit analyses									+	
• Cost of illness studies								+		
2.Aminde et al , 2018										
• EE						+	+			
• Model based EE							+			
3.Pavey et al, 2012										
• EE						+	+			
4.Ruifrok et al, 2014										
• EE		+								
5.Abu-Omar, 2017										
• Cost effectiveness(CE)										+
6.Vijay et al, 2015										
• CE						+				
7.Tonmukayakul et al, 2015										
• EE						+				

For peer review only

Conclusions derived based on summary of standard check lists used to assess the quality of economic studies in health research;

- Drummond 1996 was commonly used as the standard check list to assess the quality of studies on economic evaluations across the years (2011- 2018) and had been identified as the most appropriate on this regard [1,2, 3,6,7]
- Phillips check list has been used as the standard check list to assess the quality of model based economic evaluations[1,2,3]
- Larg & Moss check list for quality assessment in cost of illness studies has been used in review conducted recently in LMIC [1]. This is more relevant and appropriate to assess the quality of cost of illness studies.
- Quality grading has been allocated to Drummond 1996 checklist using NICE scale [2]

References

1. Murthy, S., John, D., Godinho, I. P., Godinho, M. A., Guddattu, V., & Nair, N. S. (2017). A protocol for a systematic review of economic evaluation studies conducted on neonatal systemic infections in South Asia. *Syst Rev, 6*(1), 252. doi: 10.1186/s13643-017-0648-7

2. Aminde, L. N., & Veerman, L. (2016). Interventions for the prevention of cardiovascular diseases: a protocol for a systematic review of economic evaluations in low-income and middle-income countries. *BMJ Open, 6*(12), e013668. doi: 10.1136/bmjopen-2016-013668

3. Pavey, T. G., Taylor, A. H., Fox, K. R., Hillsdon, M., Anokye, N., Campbell, J. L., Taylor, R. S. (2011). Effect of exercise referral schemes in primary care on physical activity and improving health outcomes: systematic review and meta-analysis. *BMJ, 343*. doi: 10.1136/bmj.d6462

4. Ruifrok, A. E., Rogozinska, E., van Poppel, M. N., Rayanagoudar, G., Kerry, S., de Groot, C. J., Thangaratinam, S. (2014). Study protocol: differential effects of diet and physical activity based interventions in pregnancy on maternal and fetal outcomes—individual patient data (IPD) meta-analysis and health economic evaluation. [journal article]. *Systematic Reviews, 3*(1), 131. doi: 10.1186/2046-4053-3-131

5. Abu-Omar, K., Rütten, A., Burlacu, I., Schätzlein, V., Messing, S., & Suhrcke, M. (2017). The cost-effectiveness of physical activity interventions: A systematic review of reviews. *Preventive Medicine Reports*, *8*, 72–78. http://doi.org/10.1016/j.pmedr.2017.08.006

For peer review only

6. GC, Vijay, Wilson, E. C., Suhrcke, M., Hardeman, W., & Sutton, S. (2015). Are brief interventions to increase physical activity cost-effective? A systematic review. *British Journal of Sports Medicine*. doi: 10.1136/bjsports-2015-094655

7. Tonmukayakul, U., Calache, H.,Clark, R., Wasiak, J., Faggion Jr, C.M. (2015). *Systematic review and quality appraisal of economic evaluation publications in Dentistry*. Journal of Dental research,94(10), 1348-1354 https://doi.org/10.1177/0022034515589958

For peer review only

BMJ Open

The economics of physical activity in low and middle income countries: protocol for a systematic review

Journal:	*BMJ Open*
Manuscript ID	bmjopen-2018-022686.R2
Article Type:	Protocol
Date Submitted by the Author:	09-Oct-2018
Complete List of Authors:	Ranasinghe, Priyanga; Ministry of Health, Nutrition and Indigenous Medicine, ; Brunel Univeristy London, Clinical Sciences<br>Pokhrel, Subhash; Brunel University London, Health Economics Research Group<br>Anokye, Nana; Brunel University, Health Economics Research Group
**Primary Subject Heading**:	Health economics
Secondary Subject Heading:	Public health
Keywords:	HEALTH ECONOMICS, physical activity, low and middle income countries

SCHOLARONE™
Manuscripts

Title page

The economics of physical activity in low and middle income countries: protocol for a systematic review

Priyanga Diloshini Ranasinghe[2], Subhash Pokhrel[1], Nana Kwame Anokye[1]

[1] Health Economics Theme, Institute of Environment, Health and Societies, Brunel University, London, United Kingdom

[2] Ministry of Health, Nutrition and Indigenous Medicine, Suwasiripaya, Colombo 10, Sri Lanka /Division of Health Sciences, Department of Clinical Sciences, Collage of Health and Life Sciences, Brunel University, London, United Kingdom

Corresponding author: Priyanga Diloshini Ranasinghe

Postal address: Mary Seacole 2nd floor, Division of Health Sciences, Department of Clinical Sciences, Collage of Health and Life Sciences, Brunel University, London, United Kingdom

Email: Priyanga.Ranasinghe@brunel.ac.uk/ priyangaran@yahoo.com

Telephone number: 0044 7424515136/0094 777552794

Subhash Pokhrel Subhash.Pokhrel@brunel.ac.uk

Nana Kwame Anokye Nana.Anokye@brunel.ac.uk

Word count: 2976

For peer review only

Abstract

Introduction: Evidence on the economic costs of physical inactivity and the cost-effectiveness of physical activity interventions in low- and middle-income countries (LMIC) is sparse, and fragmented where they are available. This is the first review aimed to summarize available evidence on economics of physical activity in LMIC, identify potential target variables for policy, identify and report gaps in current knowledge on economics of physical activity in LMIC.

Methods and analysis: Peer reviewed journal articles of observational, experimental, quasi experimental studies and mixed method studies on economics of physical activity in LMIC will be identified by electronic data base search; Scopus, Web of Science and SPORTDiscus. Websites of WHO, NICE international, World Bank and reference lists of included studies will be searched for relevant studies. Study selection process will be a two stage approach; title and abstract screen for inclusion, followed by review of selected full text articles by two independent reviewers. Disagreements will be resolved by consensus and discussion with a third reviewer. Data will be extracted using standardized piloted data extraction forms. Risk of bias will be critically appraised using standard checklists based on study designs. Descriptive synthesis of data is planned. Where relevant, summaries of studies will be classified according to type of economic analysis, country or country category, population, intervention, comparator, outcome and study design. Meta-analysis will be performed where appropriate. This protocol for systematic review prepared according to Preferred Reporting Items for Systematic review and Meta-analysis for Protocols (PRISMA-P) -2015 statement.

Ethics and dissemination: Ethical approval not obtained as original data will not be collected as part of this review. The completed review will be submitted for publication in a peer-reviewed journal and presentation at conferences.

Systematic review registration: PROSPERO CRD42018099856

For peer review only

Keywords: low and middle income countries; health economics; physical activity

Strengths and limitations of this study:

Strengths

- This is the first synthesis of the literature on the economics of physical activity in LMIC.
- This study provides evidence based recommendations for economic research practice on physical activity.
- This review presents evidence on the business case for physical activity interventions for decision making by policy makers in LMIC settings.

Limitations

- This review could suffer from publication bias as it excludes studies that are not peer- reviewed.
- This review focuses on studies written in English language and could miss out on relevant literature published in other languages.

INTRODUCTION

Rationale

Physical inactivity is a global health challenge, the fourth leading cause of mortality worldwide [1] and the key risk factor for non-communicable diseases (NCD) such as cardiovascular diseases, cancer and diabetes [2]. Almost one third of global health burden comprise of NCD which account for 1.4 billion disability adjusted life years (DALYs) [3]. At present most of the global NCD burden is from LMIC (as defined by World Bank, Classification 2017) [4] and this burden is largely attributable to population levels of physical inactivity [5,6]. Prevalence of physical inactivity in 2010, in upper middle income countries was 25.4% (19.1%-33.7%), followed by 16.8% (11.8%-26.4%) in lower middle

For peer review only

income countries and 16.6% (11.8%-26.4%) in low income countries showing that large population in LMIC[7] are affected by physical inactivity and are therefore at risk of developing NCDs. The data available for the year 2004 shows that 3.2 million deaths worldwide were due to physical inactivity of which 2.6 million were reported from LMIC [8]. LMIC has experienced urbanization, shift of occupations from agriculture to industrial practice and modernization including automation, and resulted in increased level of physical inactivity in these populations [9].

Increasing prevalence of physical activity and reducing the burden of NCD is a main goal of public health policies in LMIC [10]. It is a personal, cooperate and government responsibility to reduce the economic burden of unhealthy lifestyles in LMIC [10]. Economic analysis could uncover subtle characteristics of individual decision making on lifestyle choices; estimate the opportunity costs of not doing anything to improve population levels of physical activity; and help evaluate what interventions work efficiently to achieve the goal of addressing the physical inactivity pandemic, particularly in LMIC [11]. Current focus to promote physical activity has shifted from traditional educational approach to environment and policy approaches. Economic perspectives and public health perspectives complement each other in physical activity promotion [12]. Economic perspectives inform the design of effective physical activity interventions (identifying economic barriers to physical activity) and the efficient allocation of resources – critical to the economies of LMIC [12].

Our scoping exercise did not identify any published reviews that address the economics of physical activity in LMIC. Thus the economic evidence on physical activity in LMIC could be limited or scattered [13]. Therefore, we designed this review to address following research questions; what is the available evidence base and research gaps on economics of physical activity in LMIC? , what is the focus and methods underpinning economic research on physical activity in LMIC? what are the target variables and cost effective interventions in physical activity for policy in LMIC?

For peer review only

This review will add to scientific literature, an overview of economic evidence base of physical activity in LMIC and fill the gaps in available evidence on this regard. Providing an up to date synthesis of the economic evidence base is an efficient way of highlighting current research practices and new findings to inform researchers, formulation of cost effective physical activity programmes and policies.

Objectives

The review will:

- Summarize available evidence on economics of physical activity in LMIC
- Describe the focus and methods underpinning research on economics of physical activity in LMIC
- Identify potential target variables and cost effective interventions for physical activity in LMIC for policy formation
- Identify and report gaps in research on economics of physical activity in LMIC to provide recommendations for economic research agenda in LMIC

METHODS

The methods for this review were informed by previous reviews of economic analyses of physical activity [10, 13-19] and are in line with recommendations on review of economic evidence [20-22]. This will be based on "Preferred Reporting Items for Systematic Review and Meta Analyses Protocols (PRISMA-P)" Statement [23] (supplementary file 1).

For peer review only

Eligibility criteria

Economic studies on physical activity in LMIC will be included. Specifically, the following study types will be included: (a) economic evaluations of physical activity interventions, (b) economic burden of physical inactivity; (c) cost of physical activity participation, (d) demand for physical activity; (e) and economic correlates of physical (in) activity. Physical activity is defined as any bodily movement produced by skeletal muscles that require energy expenditure-including activities undertaken while walking , playing, carrying out household chores, travelling and engaging recreational pursuits[24]. Physical inactivity is considered as lack of physical activity [25]

Physical activity interventions will not be specifically defined for this review in order to identify any physical activity intervention in LMIC. However, economic analysis should be carried out along with the intervention, to be included for this review. This strategy will enable to consolidate all available evidence on economics of physical activity related to physical activity interventions.

Eligibility criteria are determined by relevant elements of PICOS criteria [26]. Interventions and comparator will be applicable only to intervention studies.

Inclusion criteria

1. Study setting: Any setting of LMIC in accordance with the definition of LMIC by World Bank, Classification 2017[4].
2. Population: Any population in any age group across the life course
3. Intervention: any physical activity intervention in which the economic evaluation of the intervention has been carried out.
4. Comparator: normal routine, no intervention
5. Outcomes: (a) cost effectiveness ratio(CE ratio), QUALY, incremental cost effectiveness ratio (ICER) assessed as the outcomes of physical activity interventions, (b) cost of physical (in) activity in terms of health care cost and/or productivity loss and/or total cost of physical

For peer review only

inactivity (c) measures of association of any economic variable with physical activity are defined as the primary outcomes of this review

6. Study design: Observational studies (cohort, case control, cross sectional); correlational studies, experimental studies including randomised controlled trials (RCTs); quasi experimental studies; natural experiments; and economic evaluation studies
7. Studies reported only in English language

Exclusion criteria

1. Case reports, case series, letters to the editor, editorials, reviews, qualitative studies unpublished theses, conference abstracts, and any unobtainable texts.
2. Studies published in a language other than English

Search strategy

The selection of databases were informed by a scoping review of methods of systematic reviews in the field [13,17,18,27,28]. We will search the following electronic data bases to identify studies: Scopus (covers 100% MEDLINE coverage, 100% of EMBASE coverage and 100% of Compendex coverage [29]), Web of Science; and SPORTDiscus. Websites of WHO, NICE international, World Bank will be searched for relevant studies. The reference lists of included studies will be searched for any relevant articles. Searches will include publications up to December 2017.

The search strategy was informed by a scoping review that covered relevant reviews on economic studies on physical activity [10,13-19], reviews on physical activity[27,30,31] and reviews on economic evaluations[28,32,33]. The draft search strategy was then reviewed by subject experts and a subject liaison librarian to optimize the sensitivity and the specificity of the search. Supplementary file 2, shows a sample search strategy.

For peer review only

Study Selection

A two stage approach involving three independent reviewers will be used to select relevant papers.

At the first stage, two reviewers (PR and NA) will independently screen the titles and abstracts of identified papers. Disagreement will be resolved through discussions with a third reviewer (SP). We will include a study if in doubt about its inclusion. For example, if the country setting of a study is unclear in the title and abstract, we will take it forward to the next stage.

At the second stage, the full text of papers selected from stage 1, will be reviewed independently by two reviewers (PR and NA). Selected and excluded papers with reasons will be discussed by the two reviewers and a third reviewer (SP) at a consensus meeting and disagreement will be resolved by real time consensus. Corresponding authors will be contacted via email for clarification if the need be. Study selection process will be presented in a PRISMA flow chart [34] along with the reasons for exclusion of studies.

Data management and extraction

Endnote X7 software will be used to manage the search results. Data extraction will be performed using standardized pilot-tested data extraction form developed based on relevant data extraction forms from relevant reviews [17,35,36]. The adaptation process has involved matching the review objectives with the available data extraction forms. Data from the final selected full text articles will be extracted by one reviewer (PR). To ensure quality of data extraction, a second reviewer (NA) will independently extract data from a random selection of the 50% of final articles. Disagreements between two reviewers will be discussed and resolved through discussions with the third reviewer (SP). Any unresolved disagreements will be reported in the final report. Supplementary file 3, shows the draft data extraction form. Data will be extracted for the following items: general information, characteristic of the study, characteristics of population/condition/intervention, data sources/data

For peer review only

analysis/outcomes, conclusions and way forward suggested by author, challenges, and quality assessment.

Risk of bias and quality assessment

Risk of bias will be assessed by a one reviewer (PR) for all selected articles using standard checklists selected based on the study design. A second reviewer (NA) will also independently assess the risk of bias on randomly selected 50% of included articles.

Checklists are considered as a reliable means of ensuring that all studies included are critically appraised in a standard way [26]. These will assess the information bias which could occur due to methods; study design, data collection, data analysis and interpretation and selection bias where relevant. Most appropriate checklist for each expected study design for risk of bias assessment were selected based on relevant literature in this area [15,17,36-40] and by consensus with all three reviewers (supplementary file 4). Risk of bias will be assessed using following tools;

- Economic evaluation studies

Drummond checklist [41] which consists of 35 items will be used. It mainly assess the quality of the study in relation to methods; study design, data collection, data analysis and interpretations. If an economic evaluation study is based on a decision analytical model, the Phillips [42] check list will be used in addition.

- Cost of illness studies,

The Larg & Moss checklist [43] will be used to assess the quality of the study. It would assess the possible biases related to analytical framework: what costs should have been measured, methodology and data: how well were resource use and productivity losses were measured

For peer review only

including representativeness of data for study population, analysis and reporting. Both internal validity and external validity will be appraised.

- Studies on correlation or association

Check list recommended by “The National Institute for Health and Care Excellence” (NICE) [44] will be used. Risk of bias related to sample selection, method of selection of exposure/comparison group, outcomes and analysis will be assessed. Both internal and external validity will be appraised.

Quality grading will be given to each included study based on overall quality of the study. Grading for internal validity and external validity will be reported separately, where relevant. The grading process will follow the NICE recommendations [37] (Figure 1):

- Good quality (++): almost all check list criteria have been fulfilled, where they have not been fulfilled the conclusions are very unlikely to alter
- Moderate quality (+) : some of the check list criteria are fulfilled, where they have not been fulfilled or not adequately described, conclusions are unlikely to alter
- Low quality (-): few or no checklist criteria are fulfilled, and conclusions are likely or very likely to alter
- The quality of the included studies will be reported and critically appraised for each study. However, studies will not be excluded based on quality, as this review is particularly aimed to capture the evidence base on economics of physical activity in LMIC and appraise the methods underpinning the evidence base.

Data synthesis and reporting

A descriptive synthesis of data is planned due to expected heterogeneity of the studies. Summaries of studies will be classified according to type of economic analysis, country or country category,

For peer review only

characteristics of the study by PICOS criteria (where applicable); population, intervention, comparator, outcome, study design and quality of studies to describe the overview of available economic evidence base of physical activity in LMIC. Where the numbers permit, summaries of the studies will be categorized based on the focus of the study; economic burden of physical inactivity, economic analysis of physical activity interventions, economic correlates of physical activity including association of physical activity and cost (health care cost and/or productivity loss). Where appropriate, we will conduct a meta-analysis to quantify the effect sizes. Such analyses will adjust for between-study heterogeneity using random effects models. The I-squared (I^2) statistic will be used to evaluate the heterogeneity related to the findings of papers. Quantitative analysis will be conducted using STATA 13 software.

We will report the number of studies where data extraction items were not applicable, as an indicator of the quality of reporting. Author stated limitations of included studies and recommendations for future research and policy will be presented. This review will be reported in accordance with PRISMA 2009 statement [34]. Study selection process will be illustrated by flow diagram including the reasons for exclusion of studies at each stage. Search strategy, data extraction form and quality assessment tools will be published as supplementary files. This protocol will not be amended and any changes will be described and discussed in final report.

Study status

Scoping review on economics of physical activity in LMIC was carried out in November 2017, to inform the methods of the current review. It identified systematic reviews conducted on economics of physical activity and the gaps in knowledge. It has not been submitted for publication. Review on economics of physical activity in LMIC was designed and methods developed in December 2017. Search strategy was piloted in January 2018. Protocol developed in February 2018. First submission

For peer review only

of protocol for peer review was on 1st March 2018. The data collection will be commenced upon approval of protocol for publication and will be completed in 2 months.

Patient and Public Involvement

Patients and public are not directly involved in this study as original data will not be collected.

ETHICS AND DISSEMINATION

Ethical clearance is not required as original data will not be collected. Best practice guidelines to conduct systematic reviews [33] will be followed. Results will be disseminated by peer reviewed publications and conference presentations.

DISCUSSION

Population level of physical inactivity in LMIC is likely to increase giving rise to multitude of public health and economic consequences, including rising healthcare costs due to increased NCD related treatments, increased disability-adjusted life years (DALYs) and productivity losses. Economic research on physical activity from the perspective of LMIC, should therefore be considered as a priority area. This is because increased physical inactivity leads to uncontrolled healthcare needs in LMICs, where resources (including that for healthcare) are limited and often shrinking in real terms. Pooling of the available research evidence on economics of physical activity in LMIC will thus reveal the current knowledge on economics of physical activity in LMIC, thereby guiding the future of national, regional and local policies around physical activity.

For peer review only

This will be the first study to review the evidence base on economics of physical activity in the context of low and middle income countries which can be considered as the main strength of this study. This will identify gaps in knowledge to support future studies in this area. However, we will include the studies published as peer reviewed articles and published in English language only. Thus, possibility of publication bias and language bias cannot be excluded. Furthermore, by assessing and reporting the quality of included studies using standard quality assessment check lists, minimize the possibility of reporting bias of this review.

WHO is in the process of drafting "Global action plan on physical activity (GAPPA) to be implemented from 2018-2030" [45]. This is a timely study as the evidence from this research will provide useful information for implementation and prioritize the actions of GAPPA in LMIC. The evidence from this research will be useful to policy makers and stakeholders dealing with physical activity promotion at individual, local, national, regional and global level with special reference to LMIC. Further, this review will be an important base for a research agenda on economics of physical activity in LMIC. This will be a useful guide for researchers to design research on economics and physical activity with sound methodology, based on the research need of LMIC. Furthermore, information gathered from this research will guide funding agencies for effective allocation of resources.

Authors Contributors: SP and NA developed the idea for the review with inputs from PR. PR wrote the first draft. SP and NA revised the protocol. NA will act as guarantor of the review.

Funding: This research received no specific grant from any funding agency in the public, commercial or not-for-profit sectors. PR is a post-doctoral fellow supported by Ministry of Health, Sri Lanka. NA and SP are employees of Brunel University London.

For peer review only

Conflicts of interest: None

Data sharing statement: All data for this manuscript included with the submission

Supplementary files

1. Comparing the study protocol with PRISMA-P 2015 check list and location in the manuscript
2. Proposed search strategy for the review (sample)
3. Data extraction form illustrating the items for which the data will be extracted and check lists for risk of bias assessment
4. Summary of tools used for risk of bias assessment of relevant literature and basis for the plan of risk of bias assessment in this review

References

1. World Health Organization. (2010). *Global Recommendations on Physical Activity for Health.* Geneva: Retrieved from: <https://www.ncbi.nlm.nih.gov/ books/NBK 305057/> [Accessed on 18 December 2017]

2. World Health Organization. (2017). *Physical activity* [Fact Sheet – *Media centre].* Retrieved from: <http://www.who.int/mediacentre/factsheets/fs385/en/ > *[Accessed on 6 February 2018]*

3. Murray, C. J., Barber, R. M., Foreman, K. J., Abbasoglu Ozgoren, A., Abd-Allah, F., Abera, S. F., Vos, T. (2015). Global, regional, and national disability-adjusted life years (DALYs) for 306

For peer review only

diseases and injuries and healthy life expectancy (HALE) for 188 countries, 1990-2013: quantifying the epidemiological transition. [Research Support, Non-U S Gov't]. *Lancet, 386*(10009), 2145-2191.

4. World Bank. (June 2017).*World Bank Country and Lending Groups: Country classification*. Retrieved from <https://datahelpdesk.worldbank.org/knowledgebase/articles/906519-world-bank-country-and-lending-groups> [Accessed on 01 December 2017]

5. Abegunde, D. O., Mathers, C. D., Adam, T., Ortegon, M., & Strong, K. (2007). The burden and costs of chronic diseases in low-income and middle-income countries. [Review]. *Lancet, 370*(9603), 1929-1938.

6. Hallal, P. C., Andersen, L. B., Bull, F. C., Guthold, R., Haskell, W., & Ekelund, U. (2012). Global physical activity levels: surveillance progress, pitfalls, and prospects. *The Lancet, 380*(9838), 247-257. doi: 10.1016/s0140-6736(12)60646-1

7. WHO. (2015). Prevalence of insufficient physical activity among adults, Data by World Bank income groups. Retrieved from <http://apps.who.int/gho/data/view.main.2487?lang=en> [Accessed on 29 January 2018]

8. WHO. (2009).*Global health risks: mortality and burden of disease attributable to selected major risks*. ISBN 978 92 4 156387 1. Retrieved from <http://www.who.int/healthinfo/global_burden_disease/GlobalHealthRisks_report_full.pdf> [Accessed on 25 January 2018]

9. Atkinson, K., Lowe, S., & Moore, S. (2016). Human development, occupational structure and physical inactivity among 47 low and middle income countries. *Preventive Medicine Reports, 3*, 40-45. doi: https://doi.org/10.1016/j.pmedr.2015.11.009

For peer review only

10. Oldridge, N. B. (2008). Economic burden of physical inactivity: healthcare costs associated with cardiovascular disease. *European Journal of Cardiovascular Prevention & Rehabilitation, 15*(2), 130-139. doi: 10.1097/HJR.0b013e3282f19d42

11. Islam, S. M. S., Purnat, T. D., Phuong, N. T. A., Mwingira, U., Schacht, K., & Fröschl, G. (2014). Non-Communicable Diseases (NCDs) in developing countries: a symposium report. *Globalization and Health*, *10*, 81. http://doi.org/10.1186/s12992-014-0081-9

12. Sturm, R. (2005). Economics and physical activity - A research agenda. *American Journal of Preventive Medicine, 28*(2), 141-149. doi: 10.1016/j.amepre.2004.10.021

13. Ding, D., Kolbe-Alexander, T., Nguyen, B., Katzmarzyk, P. T., Pratt, M., & Lawson, K. D. (2017). The economic burden of physical inactivity: A systematic review and critical appraisal. [Review]. *British Journal of Sports Medicine*, 51(19), 1392-1409. doi: 10.1136/bjsports-2016-097385

14. Jeroen C. M. Barte & G. C. Wanda Wendel-Vos (2017) A Systematic Review of Financial Incentives for Physical Activity: The Effects on Physical Activity and Related Outcomes, *Behavioral Medicine*, 43:2, 79-90, DOI: 10.1080/08964289.2015.1074880

15. Abu-Omar, K., Rütten, A., Burlacu, I., Schätzlein, V., Messing, S., & Suhrcke, M. (2017). The cost-effectiveness of physical activity interventions: A systematic review of reviews. *Preventive Medicine Reports*, *8*, 72–78. http://doi.org/10.1016/j.pmedr.2017.08.006

16. Brown, V., Zapata Diomedi, B., Moodie, M., Veerman, J., & Carter, R. (2016). A systematic review of economic analyses of active transport interventions that include physical activity benefits.*BMJ Open*. (Vol. 45). Retrieved from < http://bmjopen.bmj.com/content/6/9/e011617>

17. Pavey, T. G., Taylor, A. H., Fox, K. R., Hillsdon, M., Anokye, N., Campbell, J. L., Taylor, R. S. (2011). Effect of exercise referral schemes in primary care on physical activity and improving health outcomes: systematic review and meta-analysis. *BMJ, 343*. doi: 10.1136/bmj.d6462

18. Campbell, F., Holmes, M., Everson-Hock, E., Davis, S., Woods, H. B., Anokye, N., Kaltenthaler, E. (2015). A systematic review and economic evaluation of exercise referral schemes in primary care: a short report. *Health Technology Assessment, 19*(60), 1-+. doi: 10.3310/hta19600

19. Suhrcke, M., Boluarte, T. A., & Niessen, L. (2012). A systematic review of economic evaluations of interventions to tackle cardiovascular disease in low- and middle-income countries. [journal article]. *BMC Public Health, 12*(1), 2. doi: 10.1186/1471-2458-12-2

20. Kaltenthaler, E., Tappenden, P., & Paisley, S. (2013). Reviewing the evidence to inform the population of cost-effectiveness models within health technology assessments. [Research Support, Non-U S Gov't]. *Value Health, 16*(5), 830-836. Retrieved from < https://www.ncbi.nlm.nih.gov/pubmed/23947977>

21. Zechmeister-Koss, I., Schnell-Inderst, P., & Zauner, G. (2014). Appropriate evidence sources for populating decision analytic models within health technology assessment (HTA): a

For peer review only

systematic review of HTA manuals and health economic guidelines. [Review]. *Med Decis Making, 34*(3), 288-299. http://journals.sagepub.com/doi/abs/10.1177/0272989X13509406

22. NICE (2012). *The guideline manual Process and methods* (PMG6) Published date: November 2012. Retrieved from < https://www.nice.org.uk/process/pmg6/resources/the-guidelines-manual-appendices-bi-2549703709/chapter/appendix-b-methodology-checklist-systematic-reviews-and-meta-analyses>

23. Moher, D., Shamseer, L., Clarke, M., Ghersi, D., Liberati, A., Petticrew, M., Stewart, L. A. (2015). Preferred reporting items for systematic review and meta-analysis protocols (PRISMA-P) 2015 statement. [Research Support, Non-U S Gov't Research Support, U S Gov't, P H S]. *Syst Rev, 4*(1), 2046-4053.

24. WHO. (2018). *Physical Activity*. [Fact sheet]. Retrieved from <http://www.who.int/en/news-room/fact-sheets/detail/physical-activity > [Accessed on 15 January 2018]

25. WHO, (2018). *Global Strategy on Diet, Physical activity and Health*. Retrieved from < https://www.who.int/dietphysicalactivity/pa/en/> [Accessed on 6th October 2018]

26. University of, Y. (2009). *Systematic reviews: CRD's guidance for undertaking reviews in health care*. York: CRD, University of York. Retrieved from <https://www.york.ac.uk/media/crd/Systematic_Reviews.pdf> [Accessed on 29 December 2017]

27. Milton, K., Macniven, R., & Bauman, A. (2014). Review of the epidemiological evidence for physical activity and health from low- and middle-income countries. [Review]. *Glob Public Health, 9*(4), 369-381.

For peer review only

28. Nosratnejad, S., Rashidian, A., & Dror, D. M. (2016). Systematic Review of Willingness to Pay for Health Insurance in Low and Middle Income Countries. [Review]. *PLoS One, 11*(6).

29. Burnham, J. F. (2006). Scopus database: a review. Biomed *Digit Libr*, 3(1), 1742-5581. https://doi.org/10.1186/1742-5581-3-1

30. Lachat, C., Otchere, S., Roberfroid, D., Abdulai, A., Seret, F. M., Milesevic, J., Kolsteren, P. (2013). Diet and physical activity for the prevention of non-communicable diseases in low- and middle-income countries: a systematic policy review. [Meta-Analysis Review]. *PLoS Med, 10*(6), 11.

31. Hunter, R. F., Christian, H., Veitch, J., Astell-Burt, T., Hipp, J. A., & Schipperijn, J. (2015). The impact of interventions to promote physical activity in urban green space: a systematic review and recommendations for future research. [Research Support, Non-U S Gov't Review]. *Soc Sci Med, 124*, 246-256.

32. Sutton, L., Karan, A., & Mahal, A. (2014). Evidence for cost-effectiveness of lifestyle primary preventions for cardiovascular disease in the Asia-Pacific Region: a systematic review. *Globalization and Health*, *10*, 79. http://doi.org/10.1186/s12992-014-0079-3

33. Santatiwongchai, B., Chantarastapornchit, V., Wilkinson, T., Thiboonboon, K., Rattanavipapong, W., Walker, D. G., Teerawattananon, Y. (2015). Methodological Variation in Economic Evaluations Conducted in Low- and Middle-Income Countries: Information for Reference Case Development. *PLoS ONE, 10*(5), e0123853. doi: 10.1371/journal.pone.0123853

For peer review only

34. Moher, D., Liberati, A., Tetzlaff, J., & Altman, D. G. (2009). Preferred reporting items for systematic reviews and meta-analyses: the PRISMA statement. [Guideline Research Support, Non-U S Gov't]. *J Clin Epidemiol, 62*(10), 1006-1012.

35. Pokhrel, S., Quigley, M. A., Fox-Rushby, J., McCormick, F., Williams, A., Trueman, P., Renfrew, M. J. (2015). Potential economic impacts from improving breastfeeding rates in the UK. [Research Support, Non-U.S. Gov't]. *Arch Dis Child, 100*(4), 334-340. doi: 10.1136/archdischild-2014-306701

36. Murthy, S., John, D., Godinho, I. P., Godinho, M. A., Guddattu, V., & Nair, N. S. (2017). A protocol for a systematic review of economic evaluation studies conducted on neonatal systemic infections in South Asia. *Syst Rev, 6*(1), 252. doi: 10.1186/s13643-017-0648-7

37. Aminde, L. N., & Veerman, L. (2016). Interventions for the prevention of cardiovascular diseases: a protocol for a systematic review of economic evaluations in low-income and middle-income countries. *BMJ Open, 6*(12), e013668. doi: 10.1136/bmjopen-2016-013668

38. Ruifrok, A. E., Rogozinska, E., van Poppel, M. N., Rayanagoudar, G., Kerry, S., de Groot, C. J., Thangaratinam, S. (2014). Study protocol: differential effects of diet and physical activity based interventions in pregnancy on maternal and fetal outcomes—individual patient data (IPD) meta-analysis and health economic evaluation. [Journal article]. *Systematic Reviews, 3*(1), 131. doi: 10.1186/2046-4053-3-131

39. GC, V., Wilson, E. C., Suhrcke, M., Hardeman, W., & Sutton, S. (2015). Are brief interventions to increase physical activity cost-effective? A systematic review. *British Journal of Sports Medicine*. doi: 10.1136/bjsports-2015-094655

For peer review only

40. Tonmukayakul, U., Calache, H.,Clark, R., Wasiak, J., Faggion Jr, C.M. (2015). Systematic review and quality appraisal of economic evaluation publications in Dentistry. *Journal of Dental research*,94(10), 1348-1354. https://doi.org/10.1177/0022034515589958

41. Drummond, M. F., & Jefferson, T. O. (1996). Guidelines for authors and peer reviewers of economic submissions to the BMJ. *BMJ, 313*(7052), 275-283. doi: 10.1136/bmj.313.7052.275

42. Philips, Z., Ginnelly, L., Sculpher, M., Claxton, K., Golder, S., Riemsma, R., Glanville, J. (2004). Review of guidelines for good practice in decision-analytic modelling in health technology assessment. [Review]. *Health Technol Assess, 8*(36), 1-158.

43. Larg, A., & Moss, J. R. (2011). Cost-of-illness studies: a guide to critical evaluation. [Research Support, Non-U S Gov't]. *Pharmacoeconomics, 29*(8), 653-671.

44. National Institute for Health and Excellence (NICE). (2012). *Methods for the development of NICE public health guidance* (third edition), Appendix G Quality appraisal checklist- quantitative studies reporting correlations and associations. Retrieved from< https://www.nice.org.uk/process/pmg4/chapter/appendix-g-quality-appraisal-checklist-quantitative-studies-reporting-correlations-and >[Accessed on 02 January 2018)

45. World Health Organization. (2018). *Non communicable diseases and their risk factors, Governance: Development of draft global action plan to promote physical activity*. Retrieved from < http://www.who.int/ncds/governance/physical_activity_plan/en/> [Accessed on 10 January 2018]

For peer review only

For peer review only - http://bmjopen.bmj.com/site/about/guidelines.xhtml

For peer review only

Title page

The economics of physical activity in low and middle income countries: protocol for a systematic review

Priyanga Diloshini Ranasinghe[2], Subhash Pokhrel[1], Nana Kwame Anokye[1]

[1] Health Economics Theme, Institute of Environment, Health and Societies, Brunel University, London, United Kingdom

[2] Ministry of Health, Nutrition and Indigenous Medicine, Suwasiripaya, Colombo 10, Sri Lanka /Division of Health Sciences, Department of Clinical Sciences, Collage of Health and Life Sciences, Brunel University, London, United Kingdom

Corresponding author: Priyanga Diloshini Ranasinghe

Postal address: Mary Seacole 2nd floor, Division of Health Sciences, Department of Clinical Sciences, Collage of Health and Life Sciences, Brunel University, London, United Kingdom

Email: Priyanga.Ranasinghe@brunel.ac.uk/ priyangaran@yahoo.com

Telephone number: 0044 7424515136/0094 777552794

Subhash Pokhrel Subhash.Pokhrel@brunel.ac.uk

Nana Kwame Anokye Nana.Anokye@brunel.ac.uk

Word count: 2976

For peer review only

Abstract

Introduction: Evidence on the economic costs of physical inactivity and the cost-effectiveness of physical activity interventions in low- and middle-income countries (LMIC) is sparse, and fragmented where they are available. This is the first review aimed to summarize available evidence on economics of physical activity in LMIC, identify potential target variables for policy, identify and report gaps in current knowledge on economics of physical activity in LMIC.

Methods and analysis: Peer reviewed journal articles of observational, experimental, quasi experimental studies and mixed method studies on economics of physical activity in LMIC will be identified by electronic data base search; Scopus, Web of Science and SPORTDiscus. Websites of WHO, NICE international, World Bank and reference lists of included studies will be searched for relevant studies. Study selection process will be a two stage approach; title and abstract screen for inclusion, followed by review of selected full text articles by two independent reviewers. Disagreements will be resolved by consensus and discussion with a third reviewer. Data will be extracted using standardized piloted data extraction forms. Risk of bias will be critically appraised using standard checklists based on study designs. Descriptive synthesis of data is planned. Where relevant, summaries of studies will be classified according to type of economic analysis, country or country category, population, intervention, comparator, outcome and study design. Meta-analysis will be performed where appropriate. This protocol for systematic review prepared according to Preferred Reporting Items for Systematic review and Meta-analysis for Protocols (PRISMA-P) -2015 statement.

Ethics and dissemination: Ethical approval not obtained as original data will not be collected as part of this review. The completed review will be submitted for publication in a peer-reviewed journal and presentation at conferences.

Systematic review registration: PROSPERO CRD42018099856

For peer review only

Keywords: low and middle income countries; health economics; physical activity

Strengths and limitations of this study:

Strengths

- This is the first synthesis of the literature on the economics of physical activity in LMIC.
- This study provides evidence based recommendations for economic research practice on physical activity.
- This review presents evidence on the business case for physical activity interventions for decision making by policy makers in LMIC settings.

Limitations

- This review could suffer from publication bias as it excludes studies that are not peer- reviewed.
- This review focuses on studies written in English language and could miss out on relevant literature published in other languages.

INTRODUCTION

Rationale

Physical inactivity is a global health challenge, the fourth leading cause of mortality worldwide [1] and the key risk factor for non-communicable diseases (NCD) such as cardiovascular diseases, cancer and diabetes [2]. Almost one third of global health burden comprise of NCD which account for 1.4 billion disability adjusted life years (DALYs) [3]. At present most of the global NCD burden is from LMIC (as defined by World Bank, Classification 2017) [4] and this burden is largely attributable to population levels of physical inactivity [5,6]. Prevalence of physical inactivity in 2010, in upper middle income countries was 25.4% (19.1%-33.7%), followed by 16.8% (11.8%-26.4%) in lower middle

For peer review only

income countries and 16.6% (11.8%-26.4%) in low income countries showing that large population in LMIC[7] are affected by physical inactivity and are therefore at risk of developing NCDs. The data available for the year 2004 shows that 3.2 million deaths worldwide were due to physical inactivity of which 2.6 million were reported from LMIC [8]. LMIC has experienced urbanization, shift of occupations from agriculture to industrial practice and modernization including automation, and resulted in increased level of physical inactivity in these populations [9].

Increasing prevalence of physical activity and reducing the burden of NCD is a main goal of public health policies in LMIC [10]. It is a personal, cooperate and government responsibility to reduce the economic burden of unhealthy lifestyles in LMIC [10]. Economic analysis could uncover subtle characteristics of individual decision making on lifestyle choices; estimate the opportunity costs of not doing anything to improve population levels of physical activity; and help evaluate what interventions work efficiently to achieve the goal of addressing the physical inactivity pandemic, particularly in LMIC [11]. Current focus to promote physical activity has shifted from traditional educational approach to environment and policy approaches. Economic perspectives and public health perspectives complement each other in physical activity promotion [12]. Economic perspectives inform the design of effective physical activity interventions (identifying economic barriers to physical activity) and the efficient allocation of resources – critical to the economies of LMIC [12].

Our scoping exercise did not identify any published reviews that address the economics of physical activity in LMIC. Thus the economic evidence on physical activity in LMIC could be limited or scattered [13]. Therefore, we designed this review to address following research questions; what is the available evidence base and research gaps on economics of physical activity in LMIC? , what is the focus and methods underpinning economic research on physical activity in LMIC? what are the target variables and cost effective interventions in physical activity for policy in LMIC?

For peer review only

This review will add to scientific literature, an overview of economic evidence base of physical activity in LMIC and fill the gaps in available evidence on this regard. Providing an up to date synthesis of the economic evidence base is an efficient way of highlighting current research practices and new findings to inform researchers, formulation of cost effective physical activity programmes and policies.

Objectives

The review will:

- Summarize available evidence on economics of physical activity in LMIC
- Describe the focus and methods underpinning research on economics of physical activity in LMIC
- Identify potential target variables and cost effective interventions for physical activity in LMIC for policy formation
- Identify and report gaps in research on economics of physical activity in LMIC to provide recommendations for economic research agenda in LMIC

METHODS

The methods for this review were informed by previous reviews of economic analyses of physical activity [10, 13-19] and are in line with recommendations on review of economic evidence [20-22]. This will be based on "Preferred Reporting Items for Systematic Review and Meta Analyses Protocols (PRISMA-P)" Statement [23] (supplementary file 1).

For peer review only

Eligibility criteria

Economic studies on physical activity in LMIC will be included. Specifically, the following study types will be included: (a) economic evaluations of physical activity interventions, (b) economic burden of physical inactivity; (c) cost of physical activity participation, (d) demand for physical activity; (e) and economic correlates of physical (in) activity. Physical activity is defined as any bodily movement produced by skeletal muscles that require energy expenditure-including activities undertaken while walking , playing, carrying out household chores, travelling and engaging recreational pursuits[24]. Physical inactivity is considered as lack of physical activity [25]

Physical activity interventions will not be specifically defined for this review in order to identify any physical activity intervention in LMIC. However, economic analysis should be carried out along with the intervention, to be included for this review. This strategy will enable to consolidate all available evidence on economics of physical activity related to physical activity interventions.

Eligibility criteria are determined by relevant elements of PICOS criteria [26]. Interventions and comparator will be applicable only to intervention studies.

Inclusion criteria

1. Study setting: Any setting of LMIC in accordance with the definition of LMIC by World Bank, Classification 2017[4].
2. Population: Any population in any age group across the life course
3. Intervention: any physical activity intervention in which the economic evaluation of the intervention has been carried out.
4. Comparator: normal routine, no intervention
5. Outcomes: (a) cost effectiveness ratio(CE ratio), QUALY, incremental cost effectiveness ratio (ICER) assessed as the outcomes of physical activity interventions, (b) cost of physical (in) activity in terms of health care cost and/or productivity loss and/or total cost of physical

For peer review only

inactivity (c) measures of association of any economic variable with physical activity are defined as the primary outcomes of this review

6. Study design: Observational studies (cohort, case control, cross sectional); correlational studies, experimental studies including randomised controlled trials (RCTs); quasi experimental studies; natural experiments; and economic evaluation studies
7. Studies reported only in English language

Exclusion criteria

1. Case reports, case series, letters to the editor, editorials, reviews, qualitative studies unpublished theses, conference abstracts, and any unobtainable texts.
2. Studies published in a language other than English

Search strategy

The selection of databases were informed by a scoping review of methods of systematic reviews in the field [13,17,18,27,28]. We will search the following electronic data bases to identify studies: Scopus (covers 100% MEDLINE coverage, 100% of EMBASE coverage and 100% of Compendex coverage [29]), Web of Science; and SPORTDiscus. Websites of WHO, NICE international, World Bank will be searched for relevant studies. The reference lists of included studies will be searched for any relevant articles. Searches will include publications up to December 2017.

The search strategy was informed by a scoping review that covered relevant reviews on economic studies on physical activity [10,13-19], reviews on physical activity[27,30,31] and reviews on economic evaluations[28,32,33]. The draft search strategy was then reviewed by subject experts and a subject liaison librarian to optimize the sensitivity and the specificity of the search. Supplementary file 2, shows a sample search strategy.

For peer review only

Study Selection

A two stage approach involving three independent reviewers will be used to select relevant papers.

At the first stage, two reviewers (PR and NA) will independently screen the titles and abstracts of identified papers. Disagreement will be resolved through discussions with a third reviewer (SP). We will include a study if in doubt about its inclusion. For example, if the country setting of a study is unclear in the title and abstract, we will take it forward to the next stage.

At the second stage, the full text of papers selected from stage 1, will be reviewed independently by two reviewers (PR and NA). Selected and excluded papers with reasons will be discussed by the two reviewers and a third reviewer (SP) at a consensus meeting and disagreement will be resolved by real time consensus. Corresponding authors will be contacted via email for clarification if the need be. Study selection process will be presented in a PRISMA flow chart [34] along with the reasons for exclusion of studies.

Data management and extraction

Endnote X7 software will be used to manage the search results. Data extraction will be performed using standardized pilot-tested data extraction form developed based on relevant data extraction forms from relevant reviews [17,35,36]. The adaptation process has involved matching the review objectives with the available data extraction forms. Data from the final selected full text articles will be extracted by one reviewer (PR). To ensure quality of data extraction, a second reviewer (NA) will independently extract data from a random selection of the 50% of final articles. Disagreements between two reviewers will be discussed and resolved through discussions with the third reviewer (SP). Any unresolved disagreements will be reported in the final report. Supplementary file 3, shows the draft data extraction form. Data will be extracted for the following items: general information, characteristic of the study, characteristics of population/condition/intervention, data sources/data

For peer review only

analysis/outcomes, conclusions and way forward suggested by author, challenges, and quality assessment.

Risk of bias and quality assessment

Risk of bias will be assessed by a one reviewer (PR) for all selected articles using standard checklists selected based on the study design. A second reviewer (NA) will also independently assess the risk of bias on randomly selected 50% of included articles.

Checklists are considered as a reliable means of ensuring that all studies included are critically appraised in a standard way [26]. These will assess the information bias which could occur due to methods; study design, data collection, data analysis and interpretation and selection bias where relevant. Most appropriate checklist for each expected study design for risk of bias assessment were selected based on relevant literature in this area [15,17,36-40] and by consensus with all three reviewers (supplementary file 4). Risk of bias will be assessed using following tools;

- Economic evaluation studies

Drummond checklist [41] which consists of 35 items will be used. It mainly assess the quality of the study in relation to methods; study design, data collection, data analysis and interpretations. If an economic evaluation study is based on a decision analytical model, the Phillips [42] check list will be used in addition.

- Cost of illness studies,

The Larg & Moss checklist [43] will be used to assess the quality of the study. It would assess the possible biases related to analytical framework: what costs should have been measured, methodology and data: how well were resource use and productivity losses were measured

For peer review only

including representativeness of data for study population, analysis and reporting. Both internal validity and external validity will be appraised.

- Studies on correlation or association

Check list recommended by "The National Institute for Health and Care Excellence" (NICE) [44] will be used. Risk of bias related to sample selection, method of selection of exposure/comparison group, outcomes and analysis will be assessed. Both internal and external validity will be appraised.

Quality grading will be given to each included study based on overall quality of the study. Grading for internal validity and external validity will be reported separately, where relevant. The grading process will follow the NICE recommendations [37] (Figure 1):

- Good quality (++): almost all check list criteria have been fulfilled, where they have not been fulfilled the conclusions are very unlikely to alter
- Moderate quality (+) : some of the check list criteria are fulfilled, where they have not been fulfilled or not adequately described, conclusions are unlikely to alter
- Low quality (-): few or no checklist criteria are fulfilled, and conclusions are likely or very likely to alter
- The quality of the included studies will be reported and critically appraised for each study. However, studies will not be excluded based on quality, as this review is particularly aimed to capture the evidence base on economics of physical activity in LMIC and appraise the methods underpinning the evidence base.

Data synthesis and reporting

A descriptive synthesis of data is planned due to expected heterogeneity of the studies. Summaries of studies will be classified according to type of economic analysis, country or country category,

For peer review only

characteristics of the study by PICOS criteria (where applicable); population, intervention, comparator, outcome, study design and quality of studies to describe the overview of available economic evidence base of physical activity in LMIC. Where the numbers permit, summaries of the studies will be categorized based on the focus of the study; economic burden of physical inactivity, economic analysis of physical activity interventions, economic correlates of physical activity including association of physical activity and cost (health care cost and/or productivity loss). Where appropriate, we will conduct a meta-analysis to quantify the effect sizes. Such analyses will adjust for between-study heterogeneity using random effects models. The I-squared (I^2) statistic will be used to evaluate the heterogeneity related to the findings of papers. Quantitative analysis will be conducted using STATA 13 software.

We will report the number of studies where data extraction items were not applicable, as an indicator of the quality of reporting. Author stated limitations of included studies and recommendations for future research and policy will be presented. This review will be reported in accordance with PRISMA 2009 statement [34]. Study selection process will be illustrated by flow diagram including the reasons for exclusion of studies at each stage. Search strategy, data extraction form and quality assessment tools will be published as supplementary files. This protocol will not be amended and any changes will be described and discussed in final report.

Study status

Scoping review on economics of physical activity in LMIC was carried out in November 2017, to inform the methods of the current review. It identified systematic reviews conducted on economics of physical activity and the gaps in knowledge. It has not been submitted for publication. Review on economics of physical activity in LMIC was designed and methods developed in December 2017. Search strategy was piloted in January 2018. Protocol developed in February 2018. First submission

For peer review only

of protocol for peer review was on 1st March 2018. The data collection will be commenced upon approval of protocol for publication and will be completed in 2 months.

Patient and Public Involvement

Patients and public are not directly involved in this study as original data will not be collected.

ETHICS AND DISSEMINATION

Ethical clearance is not required as original data will not be collected. Best practice guidelines to conduct systematic reviews [33] will be followed. Results will be disseminated by peer reviewed publications and conference presentations.

DISCUSSION

Population level of physical inactivity in LMIC is likely to increase giving rise to multitude of public health and economic consequences, including rising healthcare costs due to increased NCD related treatments, increased disability-adjusted life years (DALYs) and productivity losses. Economic research on physical activity from the perspective of LMIC, should therefore be considered as a priority area. This is because increased physical inactivity leads to uncontrolled healthcare needs in LMICs, where resources (including that for healthcare) are limited and often shrinking in real terms. Pooling of the available research evidence on economics of physical activity in LMIC will thus reveal the current knowledge on economics of physical activity in LMIC, thereby guiding the future of national, regional and local policies around physical activity.

For peer review only

This will be the first study to review the evidence base on economics of physical activity in the context of low and middle income countries which can be considered as the main strength of this study. This will identify gaps in knowledge to support future studies in this area. However, we will include the studies published as peer reviewed articles and published in English language only. Thus, possibility of publication bias and language bias cannot be excluded. Furthermore, by assessing and reporting the quality of included studies using standard quality assessment check lists, minimize the possibility of reporting bias of this review.

WHO is in the process of drafting "Global action plan on physical activity (GAPPA) to be implemented from 2018-2030" [45]. This is a timely study as the evidence from this research will provide useful information for implementation and prioritize the actions of GAPPA in LMIC. The evidence from this research will be useful to policy makers and stakeholders dealing with physical activity promotion at individual, local, national, regional and global level with special reference to LMIC. Further, this review will be an important base for a research agenda on economics of physical activity in LMIC. This will be a useful guide for researchers to design research on economics and physical activity with sound methodology, based on the research need of LMIC. Furthermore, information gathered from this research will guide funding agencies for effective allocation of resources.

Authors Contributors: SP and NA developed the idea for the review with inputs from PR. PR wrote the first draft. SP and NA revised the protocol. NA will act as guarantor of the review.

Funding: This research received no specific grant from any funding agency in the public, commercial or not-for-profit sectors. PR is a post-doctoral fellow supported by Ministry of Health, Sri Lanka. NA and SP are employees of Brunel University London.

For peer review only

Conflicts of interest: None

Data sharing statement: All data for this manuscript included with the submission

Supplementary files

1. Comparing the study protocol with PRISMA-P 2015 check list and location in the manuscript
2. Proposed search strategy for the review (sample)
3. Data extraction form illustrating the items for which the data will be extracted and check lists for risk of bias assessment
4. Summary of tools used for risk of bias assessment of relevant literature and basis for the plan of risk of bias assessment in this review

Figure 1: Economics of PA in LMIC- Plan for data extraction and quality assessment process

References

1. World Health Organization. (2010). *Global Recommendations on Physical Activity for Health.* Geneva: Retrieved from: <https://www.ncbi.nlm.nih.gov/ books/NBK 305057/> [Accessed on 18 December 2017]

2. World Health Organization. (2017). *Physical activity* [Fact Sheet – *Media centre].* Retrieved from: <http://www.who.int/mediacentre/factsheets/fs385/en/ > *[Accessed on 6 February 2018]*

3. Murray, C. J., Barber, R. M., Foreman, K. J., Abbasoglu Ozgoren, A., Abd-Allah, F., Abera, S. F., Vos, T. (2015). Global, regional, and national disability-adjusted life years (DALYs) for 306

For peer review only

diseases and injuries and healthy life expectancy (HALE) for 188 countries, 1990-2013: quantifying the epidemiological transition. [Research Support, Non-U S Gov't]. *Lancet, 386*(10009), 2145-2191.

4. World Bank. (June 2017).*World Bank Country and Lending Groups: Country classification*. Retrieved from <https://datahelpdesk.worldbank.org/knowledgebase/articles/906519-world-bank-country-and-lending-groups> [Accessed on 01 December 2017]

5. Abegunde, D. O., Mathers, C. D., Adam, T., Ortegon, M., & Strong, K. (2007). The burden and costs of chronic diseases in low-income and middle-income countries. [Review]. *Lancet, 370*(9603), 1929-1938.

6. Hallal, P. C., Andersen, L. B., Bull, F. C., Guthold, R., Haskell, W., & Ekelund, U. (2012). Global physical activity levels: surveillance progress, pitfalls, and prospects. *The Lancet, 380*(9838), 247-257. doi: 10.1016/s0140-6736(12)60646-1

7. WHO. (2015). Prevalence of insufficient physical activity among adults, Data by World Bank income groups. Retrieved from <http://apps.who.int/gho/data/view.main.2487?lang=en> [Accessed on 29 January 2018]

8. WHO. (2009).*Global health risks: mortality and burden of disease attributable to selected major risks*. ISBN 978 92 4 156387 1. Retrieved from <http://www.who.int/healthinfo/global_burden_disease/GlobalHealthRisks_report_full.pdf> [Accessed on 25 January 2018]

9. Atkinson, K., Lowe, S., & Moore, S. (2016). Human development, occupational structure and physical inactivity among 47 low and middle income countries. *Preventive Medicine Reports, 3*, 40-45. doi: https://doi.org/10.1016/j.pmedr.2015.11.009

For peer review only

10. Oldridge, N. B. (2008). Economic burden of physical inactivity: healthcare costs associated with cardiovascular disease. *European Journal of Cardiovascular Prevention & Rehabilitation, 15*(2), 130-139. doi: 10.1097/HJR.0b013e3282f19d42

11. Islam, S. M. S., Purnat, T. D., Phuong, N. T. A., Mwingira, U., Schacht, K., & Fröschl, G. (2014). Non-Communicable Diseases (NCDs) in developing countries: a symposium report. *Globalization and Health*, *10*, 81. http://doi.org/10.1186/s12992-014-0081-9

12. Sturm, R. (2005). Economics and physical activity - A research agenda. *American Journal of Preventive Medicine, 28*(2), 141-149. doi: 10.1016/j.amepre.2004.10.021

13. Ding, D., Kolbe-Alexander, T., Nguyen, B., Katzmarzyk, P. T., Pratt, M., & Lawson, K. D. (2017). The economic burden of physical inactivity: A systematic review and critical appraisal. [Review]. *British Journal of Sports Medicine*, 51(19), 1392-1409. doi: 10.1136/bjsports-2016-097385

14. Jeroen C. M. Barte & G. C. Wanda Wendel-Vos (2017) A Systematic Review of Financial Incentives for Physical Activity: The Effects on Physical Activity and Related Outcomes, *Behavioral Medicine*, 43:2, 79-90, DOI: 10.1080/08964289.2015.1074880

15. Abu-Omar, K., Rütten, A., Burlacu, I., Schätzlein, V., Messing, S., & Suhrcke, M. (2017). The cost-effectiveness of physical activity interventions: A systematic review of reviews. *Preventive Medicine Reports*, *8*, 72–78. http://doi.org/10.1016/j.pmedr.2017.08.006

For peer review only

16. Brown, V., Zapata Diomedi, B., Moodie, M., Veerman, J., & Carter, R. (2016). A systematic review of economic analyses of active transport interventions that include physical activity benefits.*BMJ Open*. (Vol. 45). Retrieved from < http://bmjopen.bmj.com/content/6/9/e011617>

17. Pavey, T. G., Taylor, A. H., Fox, K. R., Hillsdon, M., Anokye, N., Campbell, J. L., Taylor, R. S. (2011). Effect of exercise referral schemes in primary care on physical activity and improving health outcomes: systematic review and meta-analysis. *BMJ, 343*. doi: 10.1136/bmj.d6462

18. Campbell, F., Holmes, M., Everson-Hock, E., Davis, S., Woods, H. B., Anokye, N., Kaltenthaler, E. (2015). A systematic review and economic evaluation of exercise referral schemes in primary care: a short report. *Health Technology Assessment, 19*(60), 1-+. doi: 10.3310/hta19600

19. Suhrcke, M., Boluarte, T. A., & Niessen, L. (2012). A systematic review of economic evaluations of interventions to tackle cardiovascular disease in low- and middle-income countries. [journal article]. *BMC Public Health, 12*(1), 2. doi: 10.1186/1471-2458-12-2

20. Kaltenthaler, E., Tappenden, P., & Paisley, S. (2013). Reviewing the evidence to inform the population of cost-effectiveness models within health technology assessments. [Research Support, Non-U S Gov't]. *Value Health, 16*(5), 830-836. Retrieved from < https://www.ncbi.nlm.nih.gov/pubmed/23947977>

21. Zechmeister-Koss, I., Schnell-Inderst, P., & Zauner, G. (2014). Appropriate evidence sources for populating decision analytic models within health technology assessment (HTA): a

For peer review only

systematic review of HTA manuals and health economic guidelines. [Review]. *Med Decis Making, 34*(3), 288-299. http://journals.sagepub.com/doi/abs/10.1177/0272989X13509406

22. NICE (2012). *The guideline manual Process and methods* (PMG6) Published date: November 2012. Retrieved from < https://www.nice.org.uk/process/pmg6/resources/the-guidelines-manual-appendices-bi-2549703709/chapter/appendix-b-methodology-checklist-systematic-reviews-and-meta-analyses>

23. Moher, D., Shamseer, L., Clarke, M., Ghersi, D., Liberati, A., Petticrew, M., Stewart, L. A. (2015). Preferred reporting items for systematic review and meta-analysis protocols (PRISMA-P) 2015 statement. [Research Support, Non-U S Gov't Research Support, U S Gov't, P H S]. *Syst Rev, 4*(1), 2046-4053.

24. WHO. (2018). *Physical Activity*. [Fact sheet]. Retrieved from <http://www.who.int/en/news-room/fact-sheets/detail/physical-activity > [Accessed on 15 January 2018]

25. WHO, (2018). *Global Strategy on Diet, Physical activity and Health*. Retrieved from < https://www.who.int/dietphysicalactivity/pa/en/> [Accessed on 6th October 2018]

26. University of, Y. (2009). *Systematic reviews: CRD's guidance for undertaking reviews in health care*. York: CRD, University of York. Retrieved from <https://www.york.ac.uk/media/crd/Systematic_Reviews.pdf> [Accessed on 29 December 2017]

27. Milton, K., Macniven, R., & Bauman, A. (2014). Review of the epidemiological evidence for physical activity and health from low- and middle-income countries. [Review]. *Glob Public Health, 9*(4), 369-381.

For peer review only

28. Nosratnejad, S., Rashidian, A., & Dror, D. M. (2016). Systematic Review of Willingness to Pay for Health Insurance in Low and Middle Income Countries. [Review]. *PLoS One, 11*(6).

29. Burnham, J. F. (2006). Scopus database: a review. Biomed *Digit Libr*, 3(1), 1742-5581. https://doi.org/10.1186/1742-5581-3-1

30. Lachat, C., Otchere, S., Roberfroid, D., Abdulai, A., Seret, F. M., Milesevic, J., Kolsteren, P. (2013). Diet and physical activity for the prevention of non-communicable diseases in low- and middle-income countries: a systematic policy review. [Meta-Analysis Review]. *PLoS Med, 10*(6), 11.

31. Hunter, R. F., Christian, H., Veitch, J., Astell-Burt, T., Hipp, J. A., & Schipperijn, J. (2015). The impact of interventions to promote physical activity in urban green space: a systematic review and recommendations for future research. [Research Support, Non-U S Gov't Review]. *Soc Sci Med, 124*, 246-256.

32. Sutton, L., Karan, A., & Mahal, A. (2014). Evidence for cost-effectiveness of lifestyle primary preventions for cardiovascular disease in the Asia-Pacific Region: a systematic review. *Globalization and Health*, *10*, 79. http://doi.org/10.1186/s12992-014-0079-3

33. Santatiwongchai, B., Chantarastapornchit, V., Wilkinson, T., Thiboonboon, K., Rattanavipapong, W., Walker, D. G., Teerawattananon, Y. (2015). Methodological Variation in Economic Evaluations Conducted in Low- and Middle-Income Countries: Information for Reference Case Development. *PLoS ONE, 10*(5), e0123853. doi: 10.1371/journal.pone.0123853

For peer review only

34. Moher, D., Liberati, A., Tetzlaff, J., & Altman, D. G. (2009). Preferred reporting items for systematic reviews and meta-analyses: the PRISMA statement. [Guideline Research Support, Non-U S Gov't]. *J Clin Epidemiol, 62*(10), 1006-1012.

35. Pokhrel, S., Quigley, M. A., Fox-Rushby, J., McCormick, F., Williams, A., Trueman, P., Renfrew, M. J. (2015). Potential economic impacts from improving breastfeeding rates in the UK. [Research Support, Non-U.S. Gov't]. *Arch Dis Child, 100*(4), 334-340. doi: 10.1136/archdischild-2014-306701

36. Murthy, S., John, D., Godinho, I. P., Godinho, M. A., Guddattu, V., & Nair, N. S. (2017). A protocol for a systematic review of economic evaluation studies conducted on neonatal systemic infections in South Asia. *Syst Rev, 6*(1), 252. doi: 10.1186/s13643-017-0648-7

37. Aminde, L. N., & Veerman, L. (2016). Interventions for the prevention of cardiovascular diseases: a protocol for a systematic review of economic evaluations in low-income and middle-income countries. *BMJ Open, 6*(12), e013668. doi: 10.1136/bmjopen-2016-013668

38. Ruifrok, A. E., Rogozinska, E., van Poppel, M. N., Rayanagoudar, G., Kerry, S., de Groot, C. J., Thangaratinam, S. (2014). Study protocol: differential effects of diet and physical activity based interventions in pregnancy on maternal and fetal outcomes—individual patient data (IPD) meta-analysis and health economic evaluation. [Journal article]. *Systematic Reviews, 3*(1), 131. doi: 10.1186/2046-4053-3-131

39. GC, V., Wilson, E. C., Suhrcke, M., Hardeman, W., & Sutton, S. (2015). Are brief interventions to increase physical activity cost-effective? A systematic review. *British Journal of Sports Medicine*. doi: 10.1136/bjsports-2015-094655

For peer review only

40. Tonmukayakul, U., Calache, H.,Clark, R., Wasiak, J., Faggion Jr, C.M. (2015). Systematic review and quality appraisal of economic evaluation publications in Dentistry. *Journal of Dental research*,94(10), 1348-1354. https://doi.org/10.1177/0022034515589958

41. Drummond, M. F., & Jefferson, T. O. (1996). Guidelines for authors and peer reviewers of economic submissions to the BMJ. *BMJ, 313*(7052), 275-283. doi: 10.1136/bmj.313.7052.275

42. Philips, Z., Ginnelly, L., Sculpher, M., Claxton, K., Golder, S., Riemsma, R., Glanville, J. (2004). Review of guidelines for good practice in decision-analytic modelling in health technology assessment. [Review]. *Health Technol Assess, 8*(36), 1-158.

43. Larg, A., & Moss, J. R. (2011). Cost-of-illness studies: a guide to critical evaluation. [Research Support, Non-U S Gov't]. *Pharmacoeconomics, 29*(8), 653-671.

44. National Institute for Health and Excellence (NICE). (2012). *Methods for the development of NICE public health guidance* (third edition), Appendix G Quality appraisal checklist-quantitative studies reporting correlations and associations. Retrieved from< https://www.nice.org.uk/process/pmg4/chapter/appendix-g-quality-appraisal-checklist-quantitative-studies-reporting-correlations-and >[Accessed on 02 January 2018)

45. World Health Organization. (2018). *Non communicable diseases and their risk factors, Governance: Development of draft global action plan to promote physical activity*. Retrieved from < http://www.who.int/ncds/governance/physical_activity_plan/en/> [Accessed on 10 January 2018]

For peer review only

For peer review only

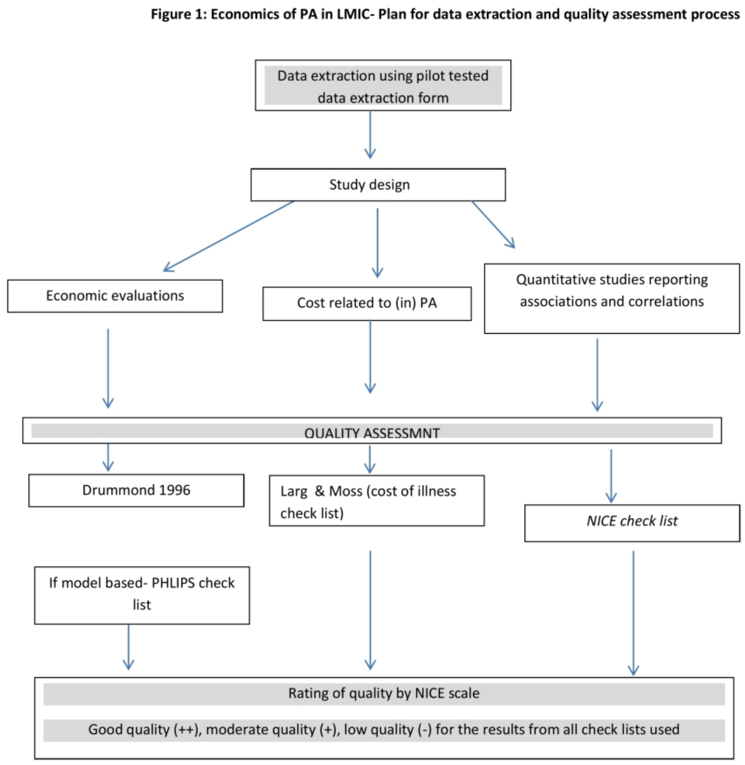

Figure 1: Economics of PA in LMIC- Plan for data extraction and quality assessment process

210x297mm (300 x 300 DPI)

PRISMA-P 2015 Checklist

This checklist has been adapted for use with systematic review protocol submissions to BioMed Central journals from Table 3 in Moher D et al: Preferred reporting items for systematic review and meta-analysis protocols (PRISMA-P) 2015 statement. *Systematic Reviews* 2015 **4**:1

Section/topic	#	Checklist item	Information reported		Line number(s)
			Yes	No	
**ADMINISTRATIVE INFORMATION**					
**Title**					
Identification	1a	Identify the report as a protocol of a systematic review	☒	☐	2
Update	1b	If the protocol is for an update of a previous systematic review, identify as such	☐	☒	
**Registration**	2	If registered, provide the name of the registry (e.g., PROSPERO) and registration number in the Abstract	☒	☐	41
**Aut4h2ors**					
Contact	3a	Provide name, institutional affiliation, and e-mail address of all protocol authors; provide physical mailing address of corresponding author	☒	☐	4-17
Contributions	3b	Describe contributions of protocol authors and identify the guarantor of the review	☒	☐	281-282
**Amendments**	4	If the protocol represents an amendment of a previously completed or published protocol, identify as such and list changes; otherwise, state plan for documenting important protocol amendments	☒	☐	236-237
**Support**					
Sources	5a	Indicate sources of financial or other support for the review	☒	☐	283-285
Sponsor	5b	Provide name for the review funder and/or sponsor	☐	☒	
Role of sponsor/funder	5c	Describe roles of funder(s), sponsor(s), and/or institution(s), if any, in developing the protocol	☐	☒	
**INTRODUCTION**					
**Rationale**	6	Describe the rationale for the review in the context of what is already known	☒	☐	57-93
**Objectives**	7	Provide an explicit statement of the question(s) the review will address with reference to participants, interventions, comparators, and outcomes (PICO)	☒	☐	86-88,94-102

For peer review only

For peer review only - http://bmjopen.bmj.com/site/about/guidelines.xhtml

BioMed Central The Open Access Publisher

Section/topic	#	Checklist item	Information reported		Line number(s)
			Yes	No	
**METHODS**					
**Eligibility criteria**	8	Specify the study characteristics (e.g., PICO, study design, setting, time frame) and report characteristics (e.g., years considered, language, publication status) to be used as criteria for eligibility for the review	☒	☐	108-141
**Information sources**	9	Describe all intended information sources (e.g., electronic databases, contact with study authors, trial registers, or other grey literature sources) with planned dates of coverage	☒	☐	142-148
**Search strategy**	10	Present draft of search strategy to be used for at least one electronic database, including planned limits, such that it could be repeated	☐	☒	152-153 Supplementary file 2
STUDY RECORDS					
Data management	11a	Describe the mechanism(s) that will be used to manage records and data throughout the review	☒	☐	166-170
Selection process	11b	State the process that will be used for selecting studies (e.g., two independent reviewers) through each phase of the review (i.e., screening, eligibility, and inclusion in meta-analysis)1	☒	☐	154-165
Data collection process	11c	Describe planned method of extracting data from reports (e.g., piloting forms, done independently, in duplicate), any processes for obtaining and confirming data from investigators	☒	☐	170-175
**Data items**	12	List and define all variables for which data will be sought (e.g., PICO items, funding sources), any pre-planned data assumptions and simplifications	☒	☐	175=178 Supplementary file 3
**Outcomes and prioritization**	13	List and define all outcomes for which data will be sought, including prioritization of main and additional outcomes, with rationale	☒	☐	129-133 Supplementary file 3
**Risk of bias in individual studies**	14	Describe anticipated methods for assessing risk of bias of individual studies, including whether this will be done at the outcome or study level, or both; state how this information will be used in data synthesis	☒	☐	179-217 Supplementary file 3
DATA					
**Synthesis**	15a	Describe criteria under which study data will be quantitatively synthesized	☐	☒	
	15b	If data are appropriate for quantitative synthesis, describe planned summary measures, methods of handling data, and methods of combining data from studies, including any planned exploration of consistency (e.g., I^2, Kendall's tau)	☒	☐	218-235
	15c	Describe any proposed additional analyses (e.g., sensitivity or subgroup analyses, meta-regression)	☐	☒	

BioMed Central The Open Access Publisher

Section/topic	#	Checklist item	Information reported		Line number(s)
			Yes	No	
	15d	If quantitative synthesis is not appropriate, describe the type of summary planned	☒	☐	218-226
**Meta-bias(es)**	16	Specify any planned assessment of meta-bias(es) (e.g., publication bias across studies, selective reporting within studies)	☐	☒	
**Confidence in cumulative evidence**	17	Describe how the strength of the body of evidence will be assessed (e.g., GRADE)	☒	☐	205-217

For peer review only

BioMed Central
The Open Access Publisher

Supplementary file 2: Proposed search strategy for Scopus

	Proposed preliminary search strategy for Scopus
#1	Domain 1: Physical activity<br><br>*"physical activity"* OR *"physical exercise"* OR *"physical inactivity"* OR *"physical fitness"* OR *"Sedentary"* OR *"active lifestyle"* OR "active *living"* OR "active *leisure"* OR "active *play"* OR "active *recreation"* OR "active *commuting"*
#2	Domain 2: Economics<br><br>*"economics"* OR *"economic evaluation"* OR *"economic burden"* OR *"health care cost"* OR *"cost analyses"* OR "cost *effectiveness"* OR "cost *benefit"* OR "cost *minimization"* OR "cost *utility"* OR "cost *saving"* OR "cost *outcome"* OR "cost *consequence"*<br><br>OR *"demand"* OR *"supply"* OR *"market"* OR *"price"* OR *"incentives"* OR *"willingness to pay"* OR *"time use"*
#3	#1 AND #2

For peer review only

Supplementary file 3

DRAFT DATA EXTRACTION FORM FOR REVIEW ON ECONONOMICS AND PYSICAL ACTIVITY IN LOW AND MIDDLE INCOME COUNTRIES (ADAPTED FROM MURTHEY ET AL., 2017, POKHREL ET AL., 2014, PAVEY ET AL., 2011)			
No.	Item category	Items	Notes
1	General information	1.1 Study ID<br>1.2 Review author ID<br>1.3 Date of review<br>1.4 Title, authors(s)<br>1.5 Year of publication<br>1.6 Country studies<br>1.7 Income level of the country	
2	Characteristics of the study	2.1 Aims and objectives/research question<br>2.2. Eligibility decision/Inclusion and exclusion criteria (?)<br>2.3 Study design<br>2.4 Study setting<br>2.5 Total study duration<br>2.6 Sample size<br>2.7 Sample size calculation<br>2.8 Recruitment<br>2.9 Sample selection<br>2.10 Method of allocation<br>2.11 Perspective (i.e. Societal, health care system, service provider etc.)	
3	Characteristics of the population/condition /intervention	**Population/participants**<br>3.1 Type of population<br>(i.e. General population university students, group of employees)<br>3.2 Age<br>3.3 Gender<br>3.4 Ethnicity<br>**Condition**<br>3.5 Definition of physical activity /physical inactivity/sedentary	

For peer review only

		3.6Definition of disease condition<br>**Intervention**<br>3.7 Type of physical activity/programme<br>3.9 Comparator	
4	Data sources/Data analysis/results	**Scope**<br>4.1 Type of analysis<br>(Ie. Costing/economic evaluation/association of economic factors with physical activity/other)<br>4.2 Form of economic evaluation<br>4.3 Time horizon<br>4.4 Study assumptions<br>**Cost**<br>4.5 Reported costs/cost components<br>4.6 Data sources for cost measures<br>4.7 How cost was reported<br>(i.e. marginal cost/ average cost/incremental cost/total cost/other)<br>4.8 Discount rate<br>4.9 Year of costing<br>4.10 Currency unit<br>4.11 Currency conversion rate<br>4.12 Inflation adjustments<br>**Outcomes**<br>4.13 Reported outcomes<br>(i.e. measures of effectiveness/efficacy/measures of effect)<br>4.14 Data sources for the outcome measures<br>4.15 Discount rate<br>**Sensitivity analysis**<br>4.16 Performed/ not<br>4.17 Type of analysis<br>(i.e. deterministic/ stochastic)<br>4.18 Variables used for sensitivity analysis<br>4.19 Results of sensitivity analysis<br>**Main results**	

For peer review only

		4.20 Cost / cost effectiveness<br>4.21 Other	
5	Conclusions and way forward	5.1 Key Conclusion(s)<br>5.2 Suggestions by author for further research	
6	Challenges	6.1 Author stated limitations<br>6.2 Author stated strengths<br>6.3 Miscellaneous comments from author<br>6.4 Funding source<br>6.5 Conflicts of interest	
7.	Quality assessment of studies	7.1 Economic evaluation studies- Drummond checklist<br>7.2 Model based economic evaluations-Phillips check list<br>7.3 Cost of illness/cost of risk factor study/cost estimation- check list by Larg and Moss<br>7.4 Quantitative study reporting correlation or association? NICE check list for quantitative studies reporting correlations and associations	

**Method of rating the quality of studies from each check list (A, B, C, D) in accordance to the quality appraisal by NICE scale (Methods for the development of NICE public health guidance (third edition))**	
• Reviewer is requested to complete in detail the comment section of quality appraisal check lists so that the grade awarded for each study aspect is as transparent as possible<br>• At the end of completion of the check list each study is awarded an overall quality grading for internal validity (IV)and a separate one for external validity (EV)	
All or most of the checklist criteria have been fulfilled, where they have not been fulfilled the conclusions are very unlikely to alter	++
Some of the checklist criteria have been fulfilled, where they have not been fulfilled, or not adequately described, the conclusions are unlikely to alter	+
Few or no checklist criteria have been fulfilled and the conclusions are likely or very likely to alter.	-

For peer review only

CHECKLIST A

QUALITY ASSESSMENT FOR ECONOMIC EVALUATION(CHECKLIST FROM DRUMMOND AND JEFFERSON 1996)	Yes	No	Not clear	Not appropriate
**Study design**				
1) The research question is stated				
2) The economic importance of the research question is stated				
3) The viewpoint(s) of the analysis are clearly stated and justified				
4) The rationale for choosing the alternative programmes or interventions compared is stated				
5) The alternatives being compared are clearly described				
6) The form of economic evaluation used is stated				
7) The choice of form of economic evaluation is justified in relation to the questions addressed				
**Data collection**				
8) The source(s) of effectiveness estimates used are stated				
9) Details of the design and results of effectiveness study are given (if based on a single study)				
10) Details of the method of synthesis or meta-analysis of estimates are given (if based on an overview of a number				

For peer review only

QUALITY ASSESSMENT FOR ECONOMIC EVALUATION(CHECKLIST FROM DRUMMOND AND JEFFERSON 1996)	Yes	No	Not clear	Not appropriate
of effectiveness studies)				
11) The primary outcome measure(s) for the economic evaluation are clearly stated				
12) Methods to value health states and other benefits are stated				
13) Details of the subjects from whom valuations were obtained are given				
14) Productivity changes (if included) are reported separately				
15) The relevance of productivity changes to the study question is discussed				
16) Quantities of resources are reported separately from their unit costs				
17) Methods for the estimation of quantities and unit costs are described				
18) Currency and price data are recorded				
19) Details of currency of price adjustments for inflation or currency conversion are given				
20) Details of any model used are given				
21) The choice of model used and the key parameters on which it is based are justified				
**Analysis and interpretation of results**				
22) Time horizon of costs and benefits is stated				
23) The discount rate(s) is stated				

For peer review only

QUALITY ASSESSMENT FOR ECONOMIC EVALUATION(CHECKLIST FROM DRUMMOND AND JEFFERSON 1996)	Yes	No	Not clear	Not appropriate
24) The choice of rate(s) is justified				
25) An explanation is given if costs or benefits are not discounted				
26) Details of statistical tests and confidence intervals are given for stochastic data				
27) The approach to sensitivity analysis is given				
28) The choice of variables for sensitivity analysis is justified				
29) The ranges over which the variables are varied are stated				
30) Relevant alternatives are compared				
31) Incremental analysis is reported				
32) Major outcomes are presented in a disaggregated as well as aggregated form				
33) The answer to the study question is given				
34) Conclusions follow from the data reported				
35) Conclusions are accompanied by the appropriate caveats				

Did the economic evaluation use a decision-analytic modelling framework?	Response: Yes(✓), No (×) , Not applicable (NA)<br>Instruction: If *Yes*, assess paper using the questions in Check list B, If *No/NA*, skip check list B

For peer review only

CHECKLIST B

QUALITY ASSESSMENT FOR DECISION-ANALYTIC MODELS(CHECKLIST FROM PHILIPS ET AL. 2004)			
**Quality criterion**	**Question(s)**	**Response (✓, ✗, NA)**	**Comments**
S1	Is there a clear statement of the decision problem?		
	Is the objective of the evaluation and model specified and consistent with the stated decision problem?		
	Is the primary decision maker specified?		
S2	Is the perspective of the model stated clearly?		
	Are the model inputs consistent with the stated perspective?		
	Has the scope of the model been stated and justified?		
	Are the outcomes of the model consistent with the perspective, scope and overall objective of the model?		
S3	Is the structure of the model consistent with a coherent theory of the health condition under evaluation?		
	Are the sources of data used to develop the structure of the model specified?		

For peer review only

QUALITY ASSESSMENT FOR DECISION-ANALYTIC MODELS(CHECKLIST FROM PHILIPS ET AL. 2004)			
**Quality criterion**	**Question(s)**	**Response (✓, ×, NA)**	**Comments**
	Are the causal relationships described by the model structure justified appropriately?		
S4	Are the structural assumptions transparent and justified?		
	Are the structural assumptions reasonable given the overall objective, perspective and scope of the model?		
S5	Is there a clear definition of the options under evaluation?		
	Have all feasible and practical options been evaluated?		
	Is there justification for the exclusion for the exclusion of feasible options?		
S6	Is the chosen model type appropriate given the decision problem and specified causal relationships within the model?		
S7	Is the time horizon of the model sufficient to reflect all important differences between options?		
	Are the time horizon of the model, the duration of treatment and the duration of treatment effect described and justified?		
S8	Do the disease states (state transition model) or the pathways (decision tree model) reflect the underlying biological process of the		

QUALITY ASSESSMENT FOR DECISION-ANALYTIC MODELS(CHECKLIST FROM PHILIPS ET AL. 2004)			
**Quality criterion**	**Question(s)**	**Response (✓, ✗, NA)**	**Comments**
	disease in question and the impact of interventions?		
S9	Is the cycle length defined and justified in terms of natural history of disease?		
D1	Are the data identification methods transparent and appropriate given the objectives of the model?		
	Where choices have been made between data sources, are these justified appropriately?		
	Has particular attention been paid to identifying data for the important parameters in the model?		
	Has the quality of the data been assessed appropriately?		
	Where expert opinion has been used, are the methods described and justified?		
D2	Is the data modelling methodology based on justifiable statistical and epidemiological techniques?		
D2a	Is the choice of baseline data described and justified?		
	Are transition probabilities calculated appropriately?		

For peer review only

QUALITY ASSESSMENT FOR DECISION-ANALYTIC MODELS(CHECKLIST FROM PHILIPS ET AL. 2004)			
**Quality criterion**	**Question(s)**	**Response (✓, ✗, NA)**	**Comments**
	Has a half-cycle correction been applied to both cost and outcome?		
	If not, has this omission been justified?		
D2b	If relative treatment effects have been derived from trail data, have they been synthesised using appropriate techniques?		
	Have the methods and assumptions used to extrapolate short-term results to final outcomes been documented and justified?		
	Have alternative extrapolation assumptions been explored through sensitivity analysis?		
	Have assumptions regarding the continuing effect of treatment once treatment is complete been documented and justified?		
	Have alternative assumptions regarding the continuing effect of treatment been explored through sensitivity analysis?		
D2c	Are the costs incorporated into the model justified?		
	Has the source for all costs been described?		
	Have discount rates been described and justified given the target decision-maker?		

For peer review only

QUALITY ASSESSMENT FOR DECISION-ANALYTIC MODELS(CHECKLIST FROM PHILIPS ET AL. 2004)			
**Quality criterion**	**Question(s)**	**Response (✓, ✗, NA)**	**Comments**
D2d	Are the utilities incorporated into the model appropriate?		
	Is the source for the utility weights referenced?		
	Are the methods of derivation for the utility weights justified?		
D3	Have all data incorporated into the model been described and referenced in sufficient detail?		
	Has the use of mutually inconsistent data been justified (i.e. are assumptions and choices appropriate)?		
	Is the process of data incorporation transparent?		
	If data have been incorporated as distributions, has the choice of distribution for each parameter been described and justified?		
	If data have been incorporated as distributions, is it clear that second order uncertainty is reflected?		
D4	Have the four principal types of uncertainty been addressed?		
	If not, has the omission of particular forms of uncertainty been justified?		
D4a	Have methodological uncertainties been addressed by running alternative versions of the model with different methodological		

For peer review only

QUALITY ASSESSMENT FOR DECISION-ANALYTIC MODELS(CHECKLIST FROM PHILIPS ET AL. 2004)			
**Quality criterion**	**Question(s)**	**Response (✓, ✗, NA)**	**Comments**
	assumptions?		
D4b	Is there evidence that structural uncertainties have been addressed via sensitivity analysis?		
D4c	Has heterogeneity been dealt with by running the model separately for different subgroups?		
D4d	Are the methods of assessment of parameter uncertainty appropriate?		
	If data are incorporated at point estimates, are the ranges used for sensitivity analysis stated clearly and justified?		
C1	Is there evidence that the mathematical logic of the model has been tested thoroughly before use?		
C2	Are any counterintuitive results from the model explained and justified?		
	If the model has been calibrated against independent data, has any differences been explained and justified?		
	Have the results of the model been compared with those of previous models and any differences in results explained?		

For peer review only

CHECKLIST C

QUALITY ASSESSMNET FOR COST OF ILLNESS STUDIES (ADAPTED FROM LARG AND MOSS, 2011)			
Responses (marked Similar to NICE check list, write the answer where relevant under the comment section)<br><br>++ Indicates that for that particular aspect of study design, the study has been designed or conducted in such a way as to minimise the risk of bias.<br>+ Indicates that either the answer to the checklist question is not clear from the way the study is reported, or that the study may not have addressed all potential sources of bias for that particular aspect of study design.<br>- Should be reserved for those aspects of the study design in which significant sources of bias may persist.<br>NR (Not Reported) - Should be reserved for those aspects in which the study under review fails to report how they have (or might have) been considered.<br>NA (Not Applicable) Should be reserved for those study design aspects that are not applicable given the study design under review (for example, allocation concealment would not be applicable for case–control studies).			
	Question	Response ***++/+/-NR/NA***	Comment
1.	Analytical framework: what costs should have been measured?<br><br>(a) What was the motivation and perspective of the study?<br><br>(b) Was the appropriate epidemiologic approach taken?<br><br>(c) Was the study question well specified?<br>(i) Were all relevant, non-trivial cost components and their stakeholders identified?<br>(ii) Were necessary timeframes specified?<br>(iii) Was a case of disease or risk factor adequately and appropriately defined?<br>(iv) Was the counterfactual population occurrence plausible and meaningful?		
2.	(2) Methodology and data: how well were resource use and productivity losses measured?		

For peer review only

	(a) Was an appropriate method(s) of quantification used, such that;<br>(i) Additional, or excess, costs were measured?<br>(ii) only costs specific to (caused by) the health problem were included (confounders controlled)?<br>(iii) All important effects were captured?<br>(iv) Important differences across subpopulations were accounted for?<br>(v) The required level of detail could be provided?<br><br>(b) Was the resource quantification method(s) well executed?<br>(i) For population-based studies, were cost allocation methods, data and assumptions valid?<br>(ii) For person-based studies, were appropriate statistical tests performed and reported?<br>(iii) Were data representative of the study population?<br>(iv) Were there any other relevant resource quantification issues?<br><br>(c) Were healthcare resources valued appropriately?<br><br>(d) Was the approach for valuing production losses justified, and assumptions valid?<br><br>(e) Was the inclusion of intangible costs appropriate:<br>(i) Was double counting of mortality-related production losses avoided?<br>(ii) Were losses valued appropriately, given the study's perspective?		
3	Analysis and reporting<br>(a) Did the analysis address the study question?<br><br>(b) Was a range of estimates presented?		

For peer review only

	(c) Were the main uncertainties identified?<br><br>(d) Was a sensitivity analysis performed on:<br><br>(i) important (uncertain) parameter estimates?<br>(ii) key assumptions? (including the counterfactual)<br>(iii) point estimates? (based on confidence or credible intervals)<br><br>(e) Was adequate documentation and justification given for cost components, data and sources, assumptions and methods?<br><br>(f) Was uncertainty around the estimates and its implications adequately discussed?<br><br>(g) Were important limitations discussed regarding the cost components, data, assumptions and methods?<br><br>(h) Were the results presented at the appropriate level of detail to answer the study question (cost components; disease subtypes, severity, stage; subpopulation groups, cost bearers)?		

For peer review only

For peer review only - http://bmjopen.bmj.com/site/about/guidelines.xhtml

CHECKLIST D

QUALITY ASSESSMENT FOR QUANTITATIVE STUDIES REPORTING CORRELATIONS AND ASSOCIATIONS(NICE,2012 BASED ON 'GRAPHICAL APPRAISAL TOOL FOR EPIDEMIOLOGICAL STUDIES(GATE)` BY JACKSON ET AL. (2006)			
Responses:<br><br>++ Indicates that for that particular aspect of study design, the study has been designed or conducted in such a way as to minimise the risk of bias.<br><br>+ Indicates that either the answer to the checklist question is not clear from the way the study is reported, or that the study may not have addressed all potential sources of bias for that particular aspect of study design.<br><br>- Should be reserved for those aspects of the study design in which significant sources of bias may persist.<br><br>NR (Not Reported) - Should be reserved for those aspects in which the study under review fails to report how they have (or might have) been considered.<br><br>NA (Not Applicable) Should be reserved for those study design aspects that are not applicable given the study design under review (for example, allocation concealment would not be applicable for case–control studies).			
**Section 1: Population**			
**Number**	**Question**	**Response**<br><br>**++/+/-/ NR/NA**	**Comments**
1.1	Is the source population or source area well described?<br><br>Was the country (e.g. developed or non-developed, type of health care system), setting (primary schools, community centres etc), location (urban, rural), population demographics etc adequately described?		

For peer review only

1.2	Is the eligible population or area representative of the source population or area?<br><br>Was the recruitment of individuals, clusters or areas well defined (e.g. advertisement, birth register)?<br><br>Was the eligible population representative of the source? Were important groups underrepresented?		
1.3	Do the selected participants or areas represent the eligible population or area?<br><br>Was the method of selection of participants from the eligible population well described?<br><br>What % of selected individuals or clusters agreed to participate? Were there any sources of bias?<br><br>Were the inclusion or exclusion criteria explicit and appropriate?		
**Section 2: Method of selection of exposure (or comparison) group**			
2.1	Selection of exposure (and comparison) group. How was selection bias minimised?<br><br>How was selection bias minimised?		
2.2	Was the selection of explanatory variables based on a sound theoretical basis?<br><br>How sound was the theoretical basis for selecting the explanatory variables?		
2.3	Was the contamination acceptably low?<br><br>Did any in the comparison group receive the exposure?		

For peer review only

	If so, was it sufficient to cause important bias?		
2.4	How well were likely confounding factors identified and controlled?<br><br>Were there likely to be other confounding factors not considered or appropriately adjusted for?<br><br>Was this sufficient to cause important bias?		
**Section 3: Outcomes**			
3.1	Were the outcome measures and procedures reliable?<br><br>Were outcome measures subjective or objective (e.g. biochemically validated nicotine levels ++ vs self-reported smoking −)?<br><br>How reliable were outcome measures (e.g. inter- or intra-rater reliability scores)?<br><br>Was there any indication that measures had been validated (e.g. validated against a gold standard measure or assessed for content validity)?		
3.2	Were the outcome measurements complete?<br><br>Were all or most of the study participants who met the defined study outcome definitions likely to have been identified?		
3.3	Were all important outcomes assessed?<br><br>Were all benefits and harms assessed?<br><br>Was it possible to determine the overall balance of benefits and harms of		

For peer review only

	intervention versus comparisons?		
3.4	Was there a similar follow-up time in exposure and comparison groups?<br><br>If groups are followed for different lengths of time, then more events are likely to occur in the group followed-up for longer distorting the comparison.<br><br>Analyses can be adjusted to allow for differences in length of follow-up (e.g. using person-years).		
3.5	Was follow-up time meaningful?<br><br>Was follow-up long enough to assess long-term benefits and harms?<br><br>Was it too long, e.g. participants lost to follow-up?		
**Section 4: Analyses**			
4.1	Was the study sufficiently powered to detect an intervention effect (if one exists)?<br><br>A power of 0.8 (i.e. it is likely to see an effect of a given size if one exists, 80% of the time) is the conventionally accepted standard.<br><br>Is a power calculation presented? If not, what is the expected effect size? Is the sample size adequate?		
4.2	Were multiple explanatory variables considered in the analyses?<br><br>Were there sufficient explanatory variables considered in the analysis?		

For peer review only

4.3	Were the analytical methods appropriate?<br><br>Were important differences in follow-up time and likely confounders adjusted for?		
4.4	Was the precision of association given or calculable? Is association meaningful?<br><br>Were confidence intervals or p values for effect estimates given or possible to calculate?<br><br>Were CIs wide or were they sufficiently precise to aid decision-making? If precision is lacking, is this because the study is under-powered?		
**Section 5: Summary**			
5.1	Are the study results internally valid (i.e. unbiased)?<br><br>How well did the study minimise sources of bias (i.e. adjusting for potential confounders)?<br><br>Were there significant flaws in the study design?		
5.2	Are the findings generalizable to the source population (i.e. externally valid)?<br><br>Are there sufficient details given about the study to determine if the findings are generalizable to the source population?<br><br>Consider: participants, interventions and comparisons, outcomes, resource and policy implications		

For peer review only

Supplementary file 4

Summary of Quality assessment tools used in systematic reviews from literature

	Quality assessment tools	Cochrane collaboration tool	Consensus health economic criteria (CHEC)	NICE	CHEERS statement	Drummond	PHILIPS	Larg & Moss cost of illness check list	Cost validity scale	National Collaborating Centre for Methods and Tools, 2008. Quality Assessment Tool for Quantitative Studies.
Systematic reviews and the type of economic analysis										
1.Murthey et al, 2017[1]										
• Trial l based economic evaluation(EE) studies		+	+		+	+				
• Model based EE				+	+		+			
• Cost benefit analyses									+	
• Cost of illness studies								+		
2.Aminde et al , 2018										
• EE						+	+			
• Model based EE							+			
3.Pavey et al, 2012										
• EE						+	+			
4.Ruifrok et al, 2014										
• EE		+								
5.Abu-Omar, 2017										
• Cost effectiveness(CE)										+
6.Vijay et al, 2015										
• CE						+				
7.Tonmukayakul et al, 2015										
• EE						+				

For peer review only

Conclusions derived based on summary of standard check lists used to assess the quality of economic studies in health research;

- Drummond 1996 was commonly used as the standard check list to assess the quality of studies on economic evaluations across the years (2011- 2018) and had been identified as the most appropriate on this regard [1,2, 3,6,7]
- Phillips check list has been used as the standard check list to assess the quality of model based economic evaluations[1,2,3]
- Larg & Moss check list for quality assessment in cost of illness studies has been used in review conducted recently in LMIC [1]. This is more relevant and appropriate to assess the quality of cost of illness studies.
- Quality grading has been allocated to Drummond 1996 checklist using NICE scale [2]

References

1. Murthy, S., John, D., Godinho, I. P., Godinho, M. A., Guddattu, V., & Nair, N. S. (2017). A protocol for a systematic review of economic evaluation studies conducted on neonatal systemic infections in South Asia. *Syst Rev, 6*(1), 252. doi: 10.1186/s13643-017-0648-7

2. Aminde, L. N., & Veerman, L. (2016). Interventions for the prevention of cardiovascular diseases: a protocol for a systematic review of economic evaluations in low-income and middle-income countries. *BMJ Open, 6*(12), e013668. doi: 10.1136/bmjopen-2016-013668

3. Pavey, T. G., Taylor, A. H., Fox, K. R., Hillsdon, M., Anokye, N., Campbell, J. L., Taylor, R. S. (2011). Effect of exercise referral schemes in primary care on physical activity and improving health outcomes: systematic review and meta-analysis. *BMJ, 343*. doi: 10.1136/bmj.d6462

4. Ruifrok, A. E., Rogozinska, E., van Poppel, M. N., Rayanagoudar, G., Kerry, S., de Groot, C. J., Thangaratinam, S. (2014). Study protocol: differential effects of diet and physical activity based interventions in pregnancy on maternal and fetal outcomes—individual patient data (IPD) meta-analysis and health economic evaluation. [journal article]. *Systematic Reviews, 3*(1), 131. doi: 10.1186/2046-4053-3-131

5. Abu-Omar, K., Rütten, A., Burlacu, I., Schätzlein, V., Messing, S., & Suhrcke, M. (2017). The cost-effectiveness of physical activity interventions: A systematic review of reviews. *Preventive Medicine Reports*, *8*, 72–78. http://doi.org/10.1016/j.pmedr.2017.08.006

For peer review only

6. GC, Vijay, Wilson, E. C., Suhrcke, M., Hardeman, W., & Sutton, S. (2015). Are brief interventions to increase physical activity cost-effective? A systematic review. *British Journal of Sports Medicine*. doi: 10.1136/bjsports-2015-094655

7. Tonmukayakul, U., Calache, H.,Clark, R., Wasiak, J., Faggion Jr, C.M. (2015). *Systematic review and quality appraisal of economic evaluation publications in Dentistry.* Journal of Dental research,94(10), 1348-1354 https://doi.org/10.1177/0022034515589958

For peer review only
